# Validation of machine learning models to detect amyloid pathologies across institutions

**DOI:** 10.1186/s40478-020-00927-4

**Published:** 2020-04-28

**Authors:** Juan C. Vizcarra, Marla Gearing, Michael J. Keiser, Jonathan D. Glass, Brittany N. Dugger, David A. Gutman

**Affiliations:** 1 The Wallace H. Coulter Department of Biomedical Engineering, Georgia Institute of Technology and Emory University, 313 Ferst Dr NW, Atlanta, GA 30332 USA; 2 Department of Neurology, Emory University School of Medicine, 12 Executive Park Dr NE, Atlanta, GA 30322 USA; 3 Department of Pathology and Laboratory Medicine, Emory University School of Medicine, 1364 Clifton Rd, Atlanta, GA 30322 USA; 4 Department of Pharmaceutical Chemistry, Department of Bioengineering and Therapeutic Sciences, Institute for Neurodegenerative Diseases, Kavli Institute for Fundamental Neuroscience, and Bakar Computational Health Sciences Institute, University of California, 675 Nelson Rising Ln, Box 0518, San Francisco, CA 94143 USA; 5 Center for Neurodegenerative Disease, Emory University School of Medicine, Whitehead Biomedical Research Building, 615 Michael Street, 5th Floor, Suite 500, Atlanta, GA 30322 USA; 6 Department of Pathology and Laboratory Medicine, University of California-Davis School of Medicine, 3400A Research Building III Sacramento, Davis, CA 95817 USA

**Keywords:** Neuropathology, Deep learning, Amyloid beta, Alzheimer’s disease, Concomitant diagnosis, Whole-slide imaging

## Abstract

Semi-quantitative scoring schemes like the Consortium to Establish a Registry for Alzheimer’s Disease (CERAD) are the most commonly used method in Alzheimer’s disease (AD) neuropathology practice. Computational approaches based on machine learning have recently generated quantitative scores for whole slide images (WSIs) that are highly correlated with human derived semi-quantitative scores, such as those of CERAD, for Alzheimer’s disease pathology. However, the robustness of such models have yet to be tested in different cohorts. To validate previously published machine learning algorithms using convolutional neural networks (CNNs) and determine if pathological heterogeneity may alter algorithm derived measures, 40 cases from the Goizueta Emory Alzheimer’s Disease Center brain bank displaying an array of pathological diagnoses (including AD with and without Lewy body disease (LBD), and / or TDP-43-positive inclusions) and levels of Aβ pathologies were evaluated. Furthermore, to provide deeper phenotyping, amyloid burden in gray matter vs whole tissue were compared, and quantitative CNN scores for both correlated significantly to CERAD-like scores. Quantitative scores also show clear stratification based on AD pathologies with or without additional diagnoses (including LBD and TDP-43 inclusions) vs cases with no significant neurodegeneration (control cases) as well as NIA Reagan scoring criteria. Specifically, the concomitant diagnosis group of AD + TDP-43 showed significantly greater CNN-score for cored plaques than the AD group. Finally, we report that whole tissue computational scores correlate better with CERAD-like categories than focusing on computational scores from a field of view with densest pathology, which is the standard of practice in neuropathological assessment per CERAD guidelines. Together these findings validate and expand CNN models to be robust to cohort variations and provide additional proof-of-concept for future studies to incorporate machine learning algorithms into neuropathological practice.

## Introduction

The aging population around the world is increasing; a 2017 study reported the population of people aged 60 or older was over 962 million and expected to double by 2050 [1]. Given the risk for developing a neurodegenerative disease increases with age, there is a critical need to better understand the underlying pathobiology of such disorders [2]. Neurodegenerative diseases are a heterogeneous group of conditions that manifest clinically in various functional deficits, specifically movement and cognitive deficits. Examples include: Alzheimer’s Disease (AD), dementia with Lewy bodies (DLB), and frontotemporal degeneration which manifest neuropathologically as beta-amyloid plaques and tau-immunoreactive neurofibrillary tangles (AD), alpha-synuclein-positive Lewy bodies (DLB, pathologically Lewy body disease - LBD), and inclusions positive for TDP-43, tau, or other entities (frontotemporal lobar degeneration of various subtypes) [3].

The “gold” standard diagnosis for neurodegenerative diseases, such as AD, is provided upon visual inspection of carefully prepared autopsied brain tissue on glass slides. Tissue regions are carefully selected and typically immunohistochemically stained for known pathological hallmarks of neurodegenerative diseases. In AD, the main hallmarks are amyloid plaques and tau neurofibrillary tangles [4, 5]. Semi-quantitative scoring strategies are used to determine if there is sufficient pathological burden to diagnose AD [6]. The Consortium to Establish a Registry for Alzheimer’s Disease (CERAD) assesses neuritic plaques (often on silver stains) in the highest density region of the neocortex [7, 8] while Thal amyloid phasing instead focuses on the distribution of β-amyloid-immunoreactive deposits across the brain for disease staging [9].

Whole-slide imaging (WSI) is an increasingly popular imaging modality used in pathology research that allows users the ability to pan and zoom around tissue directly from their computer [10]. WSI opens up opportunities to use computational approaches to quantify pathology in tissue slides for scoring purposes, which could reduce the time-consuming workflow common in most histological studies. Inter- and intra-rater variability between observers for these semi-quantitative measures can be often quite high, further calling into question the validity of a gold-standard [11]. Studies have shown this variability arises from a combination of factors that include a lack of stringent tissue preparation standards used across labs and simple human subjectivity [12]. Any proposed computational approach implemented to tackle these challenges would need to be robust, impartial, consistent, and scalable in order to be successful.

The use of machine learning in the field of histopathology [13–16] has shown great promise, and may in part help standardize quantitative assessment in neurodegenerative disorders. Convolutional neural networks (CNN), a class of machine learning models, are excellent for working with imaging data and have recently been shown to be capable of quantifying AD pathology comparable to an expert neuropathologist [17]. In other work, CNNs identified a diverse group of tau morphologies in WSI with good comparison to expert annotations [18]. While these examples are promising for the field, they are not yet part of the standard practice in scoring tissue slides. One of the biggest remaining challenges is the robustness of these methods across larger and more diverse cohorts. Most current and previous works using machine learning in histopathology focus on well-defined imaging cohorts from one source (i.e. institution). For machine learning approaches to gain traction, they need to display success across sources with minimal cohort adjustment. In histopathology, this is of critical concern as variations in cohorts can arise from selection bias in addition to various non-biological factors: techniques used throughout the staining process, skill of the scientist, post-mortem interval, variations in tissue processing, etc. [12, 19]. Since CNN’s require well-defined training data, it is not uncommon for a “well trained” model to perform poorly when applied to new datasets. In this work, we evaluated how a model trained exclusively on images from a single University [17] would perform on a completely independent cohort. We further add depth to this pipeline to improve our understanding of its potential benefits for neurological disease research.

The pipeline in question is from the work published in Tang et al. 2019 [17]. This pipeline uses a multiclass CNN model to classify images for three AD pathologies (cored plaques, diffuse plaques, and cerebral amyloid angiopathy (CAA)). The output of the pipeline generates confidence heatmaps of the entire WSI for each of the three pathologies. Each of these heatmaps are converted to a quantitative score representing the percentage of each amyloid pathology present in the WSI (three CNN scores per WSI), which are subsequently compared to semi-quantitative scores of the pathologies. The published pipeline showed good comparison results between the CNN-scores and CERAD-like scores (we utilize the term CERAD-like, to distinguish from the original CERAD criteria which was utilized for neuritic plaques and we adapted this for Aβ-immunostained sections using a similar semi-quantitative scale [7]) for each pathology on a 30 WSI dataset. We show in this work that this pipeline is robust and performs well on a separate institutional cohort (40 WSI dataset) without any re-training (i.e. “as is”). Modifications to the pipeline to further investigate various aspects and measure its potential utility in practice were also evaluated.

The current study aimed to accomplish the following: 1) determine the robustness of the CNN pipeline on a new cohort, 2) determine the effects of pathologic heterogeneity on CNN scores, 3) determine the effects of anatomic area segmentations (gray matter vs whole tissue) analysis on CNN scores and 4) compare CNN-scores at the whole slide level vs highest density regions. The reasoning for point 1 is explained above. Point 2 is of interest in investigating the predictive power of CNN scores as it pertains to other categorization criteria. Neurodegenerative pathological presentation is heterogeneous which often makes it difficult to cleanly categorize cases. AD for example can be seen alongside other pathologies, such as alpha synuclein deposits (Lewy bodies) and / or TDP-43 inclusions [6, 20–23]. Of interest is whether scores based solely on Aβ burden are affected by presentation of secondary conditions, and whether these cases are still clearly differentiable from control or healthy brains. Comparing CNN scores to the NIA-Reagan criteria, which rank the probability that clinical dementia is due to AD, will provide further information regarding the utility of this CNN score [24, 25]. Point 3 is of interest because the method applied in Tang et al. focused on the entire tissue section while many pathologies are most prominent in the gray matter. By limiting our analysis to the gray matter we (a) hypothesized that reduction in noise may arise from an imbalance of white / gray matter ratio between images and (b) assessed whether amyloid deposits obtained outside the gray matter hold significant importance in comparison with pathological diagnosis [5, 26, 27]. Lastly, we investigate point 4 to address the concern of introducing a single score, based on the entire region of interest (tissue / gray matter) as opposed to the CERAD approach of looking at the highest density region.

## Materials and methods

### Data

**Fig. 1:**
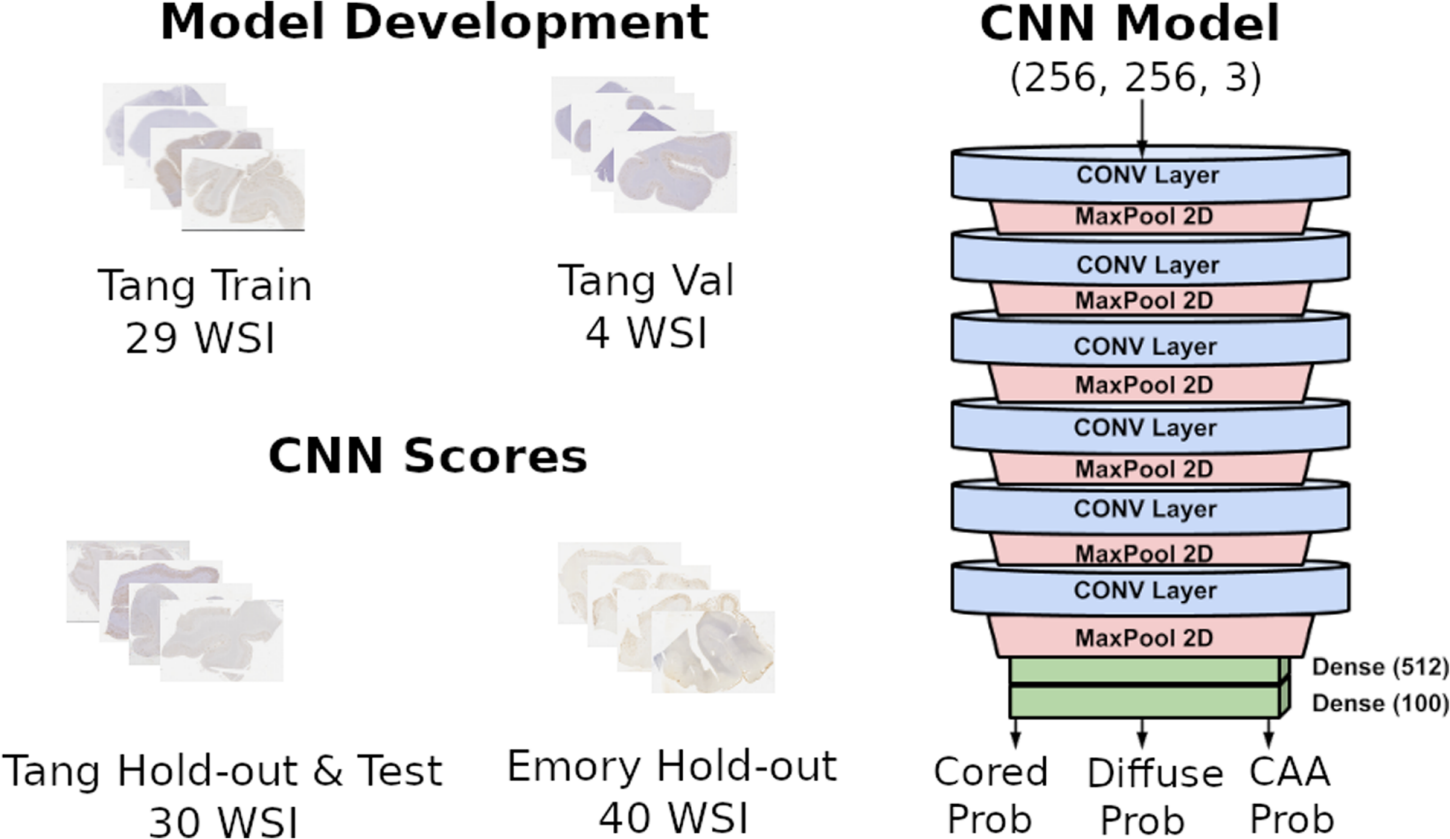
The Tang dataset was broken down into four unique datasets. The Train (29 WSI) and Val (4 WSI) datasets were used for training a CNN model. The Test (10 WSI) was used to test the performance of the trained model (i.e. generated ROC and PRC curves). These three datasets went through the process of tiling, which extracts small images (256 by 256 color images) and labels them for their inclusions of amyloid deposits. The Test dataset with the addition of the Tang Hold-out (20 WSI) dataset where used to generate confidence heatmaps and CNN scores for each of the three amyloid morphologies (for each WSI). The Emory dataset (40 WSI) was used to also generate confidence heatmaps and CNN scores. The CNN model architecture is shown on the right. The architecture includes six convolutional layers with max pooling layers and with two dense layers (512 and 100 nodes respectively) at end. The CNN model inputs are red-green-blue 256 by 256 images and it outputs three class probabilities (one for each amyloid pathology)

Data used in Tang et al. is available at 10.5281/zenodo.1470797. The data comprises 63 subjects, with one temporal gyri WSI for each subject. The subjects were selected to contain a wide breadth of pathological burden for each of the three AD pathologies of interest: cored and diffuse plaques, in addition to cerebral amyloid angiopathy (CAA). The WSIs were all immunohistochemically stained using an amyloid Beta (Aβ) antibody (4G8 from Biolegend, San Diego, CA) with diaminobenzidine for color development, and counterstained with hematoxylin for nuclei visualization. The dataset contains cases spanning the spectrum of AD pathology burden, including cases lacking cognitive impairment as well as cases lacking AD pathology. Also provided are the 70,000 previous images (tiles of size of 256 × 256 pixels) that were selected and labeled for plaque type content, that can be used to train a new model (Additional file 2: Figure S1). This image dataset was extracted from a 29 WSI training dataset (61,370 images) and a 4 WSI validation dataset (8630 images). Another 10,873 labeled images were provided to test the trained model, obtained from the 10 WSI testing dataset. The remaining 20 WSI are provided for generating the confidence heatmaps. The Emory dataset comprises 40 WSI selected by an expert neuropathologist (MG) containing a range of pathology burden for each of the AD pathologies of interest (Table 1). The Emory cases contained 5 cases with no significant neuropathological changes and no cognitive impairment (healthy/control brain), 27 with a primary neuropathological diagnosis of AD, 4 with a primary neuropathological diagnosis of Lewy body disease (of the LBD cases, 2 had a secondary diagnosis of AD or probable AD). Of cases with a primary diagnosis of AD, 8 also had TDP-43-positive inclusions, 5 had limbic or neocortical LBD, and 6 had amygdala-predominant Lewy bodies; among these there was some overlap with 1 case having TDP-43-positive inclusions and LBD, and 3 cases having TDP-positive inclusions and amygdala-predominant Lewy bodies (Additional file 1). Two cases had a clinical diagnosis of control (also listed as the primary neuropathologic diagnosis) and a (secondary) neuropathologic diagnosis of possible AD. These are cases who showed normal cognition clinically but were found to have AD pathology at autopsy. All whole slide images consisted of glass slides of 8 μm formalin fixed paraffin embedded sections of the temporal gyri immunohistochemically labeled with an antibody to Aβ (4G8; Biolegend, San Diego, CA), utilizing 3,3′-diaminobenzidine (DAB) for color development with hematoxylin counterstain, similar to the previous cohort. Glass slides with tissue were cleaned with 70% ethanol solution prior to scanning with an Aperio AT2 DX system at 20x magnification. All WSI were uploaded to a local server and accessed for viewing using a local instance of the Digital Slide Archive platform [28]. Figure 1 shows a visual representation of the breakdown of datasets used in this project.

**Table 1:** Number of cases for each category of CERAD-like score for each Aβ pathology assessed in the Emory dataset

	Cored Plaques	Diffuse Plaques	CAA
None	10	6	19
Sparse	13	3	5
Moderate	14	3	6
Frequent	3	28	10

Each WSI / case was given three CERAD-like scores (for cored plaque, diffuse plaque, and CAA). The CERAD-like scores are semi-quantitative with four possible categories: none, sparse, moderate and frequent. We utilized the term CERAD-like to not confuse these data with CERAD scores - CERAD was initially meant for semi-quantitative analysis of neuritic plaques in multiple brain regions, and data analyzed here is for Aβ deposits only within the temporal cortex l. All scores were provided by a single neuropathologist (BD) viewing the slides using the Digital Slide Archive platform. This table provides information about the number of cases in each CERAD-like category for each Aβ morphology / score. Further detail of all cases are given in Additional file 1

### Model training recreation

CNN model weights were provided by *Tang* et al. and were used to load the pre-trained model and generate confidence heatmaps. A new model was also trained from the *Tang* et al. labeled image dataset by using the provided code (https://github.com/keiserlab/plaquebox-paper) and training data (10.5281/zenodo.1470797). The new model performance was assessed with receiver operating curves and precision recall curves, which showed good performance on both the validation and testing set (Additional file 2: Figure S1). All CNN code is implemented using Python’s open source PyTorch package [29]. A Docker container was used to run all the code to allow easy replication of our results using the same OS & Python environment [30]. For a detailed description on how the training, validation, and testing dataset was obtained from the WSI, see [17]. Figure 1 shows a representation of the CNN model architecture for reference.

### WSI preprocessing

Reinhard color normalization was applied to all images prior to analysis, using the same reference image for all images [31]. The PyVips library was used to apply the color normalization and subsequently tile the WSI into small images in a structured format. This tiling was later used to create the confidence heatmaps using the trained model.

### Confidence Heatmap & CNN scores

The detailed methods for CNN and heatmap generation have been previously reported [17]. Briefly, the trained CNN model was used in a sliding window approach to create WSI confidence heatmaps [32]. A stride of 16 pixels was used to generate the confidence heatmaps. For each WSI, a confidence heatmap was generated for each pathology (cored plaques, diffuse plaques, CAA), with high probabilities signaling the intensity of the pathology present. The sliding window approach results in confidence heatmaps at a fraction of the resolution of the original WSI. The smaller size makes it possible to run the computational analysis on modern standard computers equipped with GPU(s) for easy reproducibility without excessive loss of information.

Confidence heatmaps were used to generate CNN scores by a process of thresholding, binary operations, and object labeling. For each WSI three CNN scores were generated for the percentage of cored plaques, diffuse plaques, and CAA present in the WSI. The OpenCV Python package was used to perform the cleaning and blob labeling [33]. Using specific thresholds for each plaque type, confidence heatmaps are converted to binary masks. Probabilities below the threshold are zeroed and considered noise. Binary operations, opening and closing, follow to clean up the image and a blob labeling approach is used to group nearby pixels together. The foreground tissue area is segmented by application of lightness-chroma-hue (LCH) color space thresholding. Minimal LCH parameter tuning is required for each image to segment the foreground accurately to account for slide staining variations. The CNN score provided is then calculated as the number of unique blob labels divided by pixels in tissue area.

### CNN score comparisons

Emulating traditional CERAD style scoring, an expert neuropathologist (BD) scored each WSI on a semi-quantitative scale (none, sparse, moderate, and frequent) for each AD pathology (cored, diffuse and CAA). Unlike CERAD, the scores were assessed on a single slide (temporal region only rather than multiple regions of neocortex) immunohistochemically labeled with anti-Aβ (rather than histochemically stained with silver or thioflavin stains) and given by the overall density (rather than the region with densest pathology as is common practice by the CERAD criteria) [7, 8, 34]. These so-called CERAD-like scores are used as our ground-truth comparison for the CNN scores, to get an interpretable measure of how well the model and heatmap pipeline can detect specific AB morphologies and how well it performs in comparison with human semi-quantitative scoring. For statistical analysis, we grouped the WSI into their respective CERAD-like groups and performed an ANOVA with post-hoc analysis using Tukey’s test between each group to test significant differences between the groups. All statistics were implemented using Python’s open-source libraries: statsmodels and scipy. This was repeated for each of the three pathologies of interest. ANOVA F-statistic and *p*-value is reported as well as the Tukey’s test *p*-values between adjacent groups (none vs sparse, sparse vs moderate, moderate vs frequent) with values less than 0.05 considered statistically significant. This process was run on the Emory dataset (*n* = 40), Tang et al*’*s dataset (test + hold-out *n* = 30), and the combined dataset (*n* = 70).

CNN score comparisons were also analyzed for the Emory data exclusively in two alternative ways. 1) The pathological diagnosis for the Emory dataset was used to group the cases: control (no significant pathology) vs pure AD (only AD diagnosis), control vs all cases with AD pathology, and pure AD vs cases with AD and TDP-43 inclusions or LBD pathology. For our analysis, diagnoses of probable AD and possible AD were considered as AD diagnoses. The two cases that showed AD pathology during autopsy but no cognitive impairment clinically, were excluded from this analysis (Additional file 1, cases 5 & 8) but the analysis with their inclusion can be found in Additional file 2. Furthermore, cases with amygdala-predominant Lewy body disease were not included in the LBD group. The pathological diagnoses are reported as primary, secondary, and tertiary, and for the purposes of analysis, only presence or absence was considered for grouping (Additional file 1). 2) CNN scores were grouped by NIA-Reagan scores for each case (provided by MG) that indicate the likelihood of a diagnosis of AD as no, low, intermediate, or high likelihood [24, 35].

### Gray matter annotations

The HistomicsTK package, part of the Digital Slide Archive, was used to manually annotate and segment the gray matter regions of the WSI for the Emory dataset [28]. Corresponding Bielschowsky silver stain images were scanned from the same brain region to facilitate visual recognition of gray matter / white matter regions. Only areas that were clearly gray matter were annotated and artifacts and tissue abnormalities (tears etc.) were purposely avoided / excluded in the annotations. The manual annotation process was verified by an expert neuropathologist for correctness (MG). The confidence heatmap analysis was modified by the addition of a masking step prior to blob detection. We applied the binary mask of the gray matter on the confidence heatmap to remove amyloid deposits outside this region. New CNN scores were generated taking the subset of plaques occurring exclusively in the gray matter regions and the pixel area of the gray matter only. All comparisons with CERAD-like scores, pathological diagnosis, and NIA-Reagan scores were repeated using these new CNN scores.

### CERAD-like CNN score comparisons

CNN scores were calculated for small regions in the heatmaps of area approximately equal to 4 mm^2^ (251 by 251 pixels) on the original WSI. This area is similar to the 10x magnification field of view used when scoring slides based on CERAD criteria [7]. A field-of-view CNN score was provided for each of these small regions with a stride of 16 pixels between regions to identify the field-of-view with the highest CNN-score. Correlation between the *n*-highest field-of-views and the whole tissue CNN scores were provided to identify the number of field-of-views needed for convergence. The highest field-of-view CNN score was also compared in the same way that the whole tissue CNN-score was.

## Results

### CNN scores grouped by CERAD-like categories

**Fig. 2:**
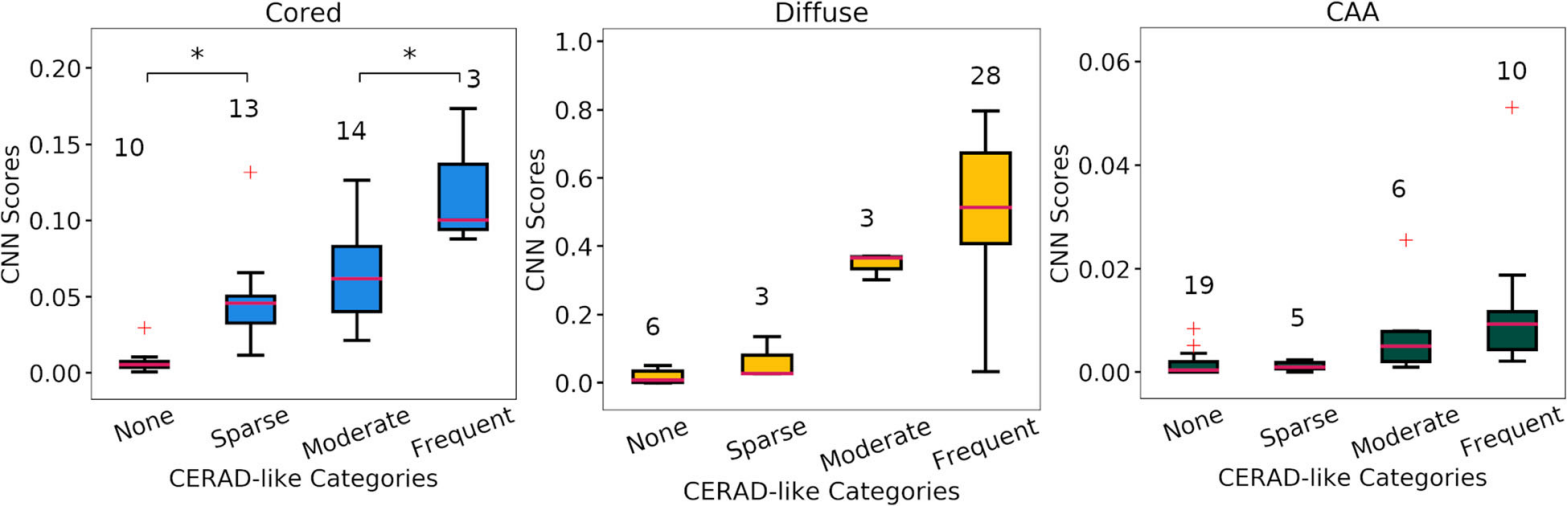
CNN scores for the Emory data generated from confidence heatmaps grouped by CERAD-like categories. Left boxplot shows comparison for cored, middle for diffuse, and right for CAA pathologies. An ANOVA with post-hoc analysis using Tukey’s test was used to assess significance with an alpha value of 0.05, significance is shown between groups with * for *p*-value less than 0.05. Whiskers show the interquartile range of +/− 1.5*IQR. Outliers are shown as red + and medians are shown as horizontal red lines in the boxplots

The model trained in this work shows similar results to those published with the trained model in Tang et al. (Additional file 2: Figure S1). For all following comparisons, the trained model in Tang et al. was used to generate confidence heatmaps for the Emory data. CNN scores grouped by their CERAD-like categories for the Emory data are shown in Fig. 2. Cored plaques show an increasing trend in CNN score with increasing severity of CERAD-like score (none, sparse, moderate, and frequent) (F-statistic: 14.1, *p*-value: 3E-6). Post-hoc analysis using Tukey’s test show statistical significance between none and sparse groups and between moderate and frequent groups. Sparse and moderate groups were not significantly different, but there was a trend of increasing CNN score. Diffuse plaques show no statistical significance between adjacent groups (none, vs sparse, sparse vs moderate, moderate vs frequent) but are significantly different in non-adjacent groups (F-statistic: 17.4, *p*-value: 3E-7). CAA scores show very small values for all groups with multiple high score outliers in the none and one on the moderate and frequent groups each (F-statistic: 4.8, *p*-value: 0.007). All statistical significance is provided by an ANOVA with post-hoc analysis using Tukey’s test with an alpha value of 0.05. A Krusal-Wallis one-way analysis of variance was also performed for CAA due to various outliers and also showed significance (*p*-value: 0.00016).

We also compared CNN scores grouped by CERAD-like categories for the Emory dataset vs. the Tang 30 WSI dataset, showing similar scores in each category (Additional file 2: Figure S2). Combining the datasets and performing the same comparison as above showed more pronounced differences among the groups (Additional file 2: Figure S3). Cored plaques displayed more pronounced differences across all CERAD-like groups, likely due to the increased sample size (F-value: 29.8, p-value: 2E-12). Diffuse plaques showed significance between sparse and moderate groups and moderate and frequent groups, which were not previously observed in the original smaller cohort (F-value: 29.2, *p*-value: 3E-12). CAA plaques still showed very little variation between the groups, with a few cases in the “moderate” and “frequent” groups containing high levels of CAA (F-value: 13.6, p-value: 5E-7).

### CNN scores grouped by Diagnosis & Reagan Criteria

The pathological diagnoses for each case can be found in Additional file 1. Of interest is whether CNN scores clearly differentiate cases with a clinicopathological diagnosis of AD (as a primary or secondary diagnosis) from those without, otherwise referred to as control cases (no significant AD present). Three groups were identified: control group (healthy brain, *n* = 5), pure AD (no secondary diagnosis, *n* = 14), and all AD (pure AD cases plus cases with AD and secondary diagnosis of LBD and/or TDP-43 inclusions, *n* = 30). The AD groups can be further broken down into pure AD group (*n* = 14), AD+LBD group (*n* = 7), and the AD+TDP group (*n* = 8). The two cases with clinically cognitive normal diagnosis but with AD pathology were excluded from this analysis (the analysis was also run with these cases included as pure AD cases and shown in Additional File 2: Figure S4, but no significant difference was observed).

**Fig. 3:**
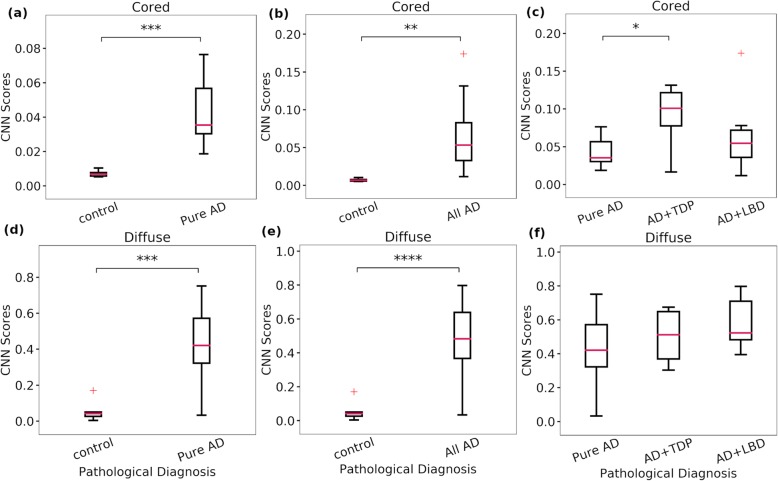
Whole Tissue CNN scores grouped by pathological diagnosis (Emory cohort). CNN scores generated from confidence heatmap processing are grouped into three distinct groups: control cases, pure AD (no secondary diagnosis), and all AD (pure AD + cases with secondary diagnosis of TDP and / or LBD). The all AD group is further divided into pure AD, AD+LBD, and AD+TDP ((c) and (f)). **a-c** Comparison for the cored CNN scores. **d-f** Comparisons for the diffuse CNN scores. AD: Alzhiemer’s disease, TDP: TDP-43 inclusions, LBD: Lewy body disease. For (**a**), (**b**), (**d**) and (**e**) a student’s 2-sided independent sample t-test was used to assess significance. For (**c**) and (**f**) an ANOVA with post-hoc analysis using Tukey’s test for multiple comparisons was used to assess significance. Alpha value of 0.05, significance is shown between groups with * for p-value less than 0.05, ** less than 0.01, *** less than 0.001, and **** less than 0.0001. Whiskers show the interquartile range of +/− 1.5*IQR. Outliers are shown as red + and medians are shown as horizontal red lines in the boxplots. Control group (*n* = 5), pure AD (*n* = 14), all AD (*n* = 30), AD+TDP (*n* = 8), and AD+LBD (*n* = 7)

Out of the 40 Emory cases, 14 cases contained pure AD and 5 were cases with no significant clinicopathological defined neurodegenerative pathology (control). Comparison between those two groups for cored and diffuse CNN scores are shown in Fig. 3. Significance was seen between the pure AD and control group and the all AD group and control, assessed with a two-sided student’s t-test. For the comparison among the pure AD, AD+TDP, and AD+LBD an ANOVA showed significance among groups for cored plaques (F-statistic: 4.8, *p*-value: 0.016) but not for diffuse plaques. Post-hoc analysis using Tukey’s test for multiple comparisons showed statistical significance between pure AD and AD+TDP groups, with AD+TDP having a higher CNN score for cored plaques than pure AD. CAA CNN scores showed no significance in any of these comparisons (Additional file 2: Figure S5).

**Fig. 4:**
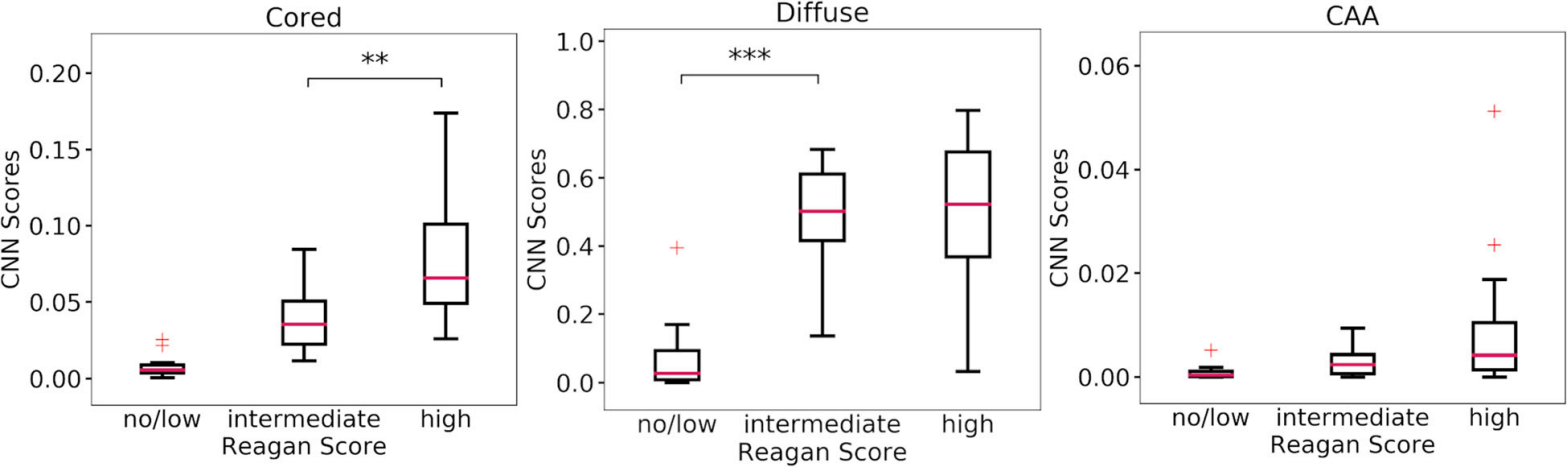
Whole tissue CNN scores for the Emory dataset are grouped together by their Reagan score, combining the no and low groups into one. Groups are compared using an ANOVA with post-hoc analysis with Tukey’s test for multiple comparisons. Outliers are shown with red + and significance between groups is shown (** 0.01, *** 0.001). no/low (*n* = 11), intermediate (*n* = 10), and high (*n* = 19)

An alternative approach is to group AD cases using the NIA Reagan criteria, which provide four levels of likelihood of Alzheimer’s disease presence: no (*n* = 7), low (*n* = 4), intermediate (*n* = 10), and high (*n* = 19) groups. For comparison of CNN scores, we grouped together the no and low groups due to the low number of samples in these groups. Significance was seen in cored plaque CNN scores (F-statistic: 19.5, p-value: 2E-6) and diffuse plaque CNN scores (F-statistic: 23.8, *p*-value: 2E-7) but not in CAA CNN scores (F-statistic: 3.0, *p*-value: 0.06). Tukey’s post-hoc test showed significance between the intermediate and high groups but not between the no/low and intermediate group for cored CNN scores. Diffuse plaques CNN scores showed significance between the no/low and intermediate group but not between the intermediate and high groups (Fig. 4).

### Gray matter analysis

**Fig. 5:**
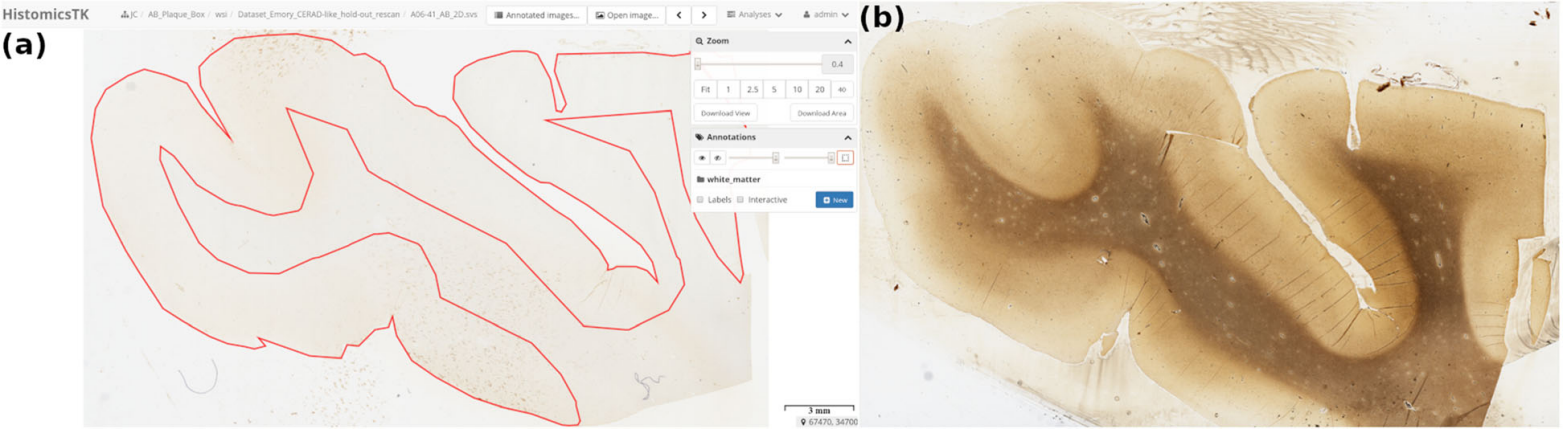
**a** The Digital Slide Archive application grants access to the HistomicsTK graphical user interface for annotation of WSI, red is the annotated region on the sample Aβ stained slide. **b** Gray matter regions can be difficult to delineate using immunohistochemical stains, in such cases we can use adjacent sliced tissue stained for Bielschowsky silver to guide the annotation. The annotations are saved in SVG format as metadata to the item containing the WSI in the Digital Slide Archive datastore

**Fig. 6:**
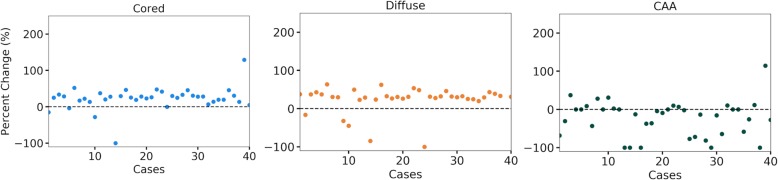
Gray matter vs Whole Tissue CNN scores for Emory dataset. Percent change is shown for the CNN scores in each AD pathology (cored, diffuse, and CAA). Cored and diffuse plaques show positive percent changes (increases) for gray matter when compared to whole tissue while CAA generally shows negative percent changes (decreases). Dashed black line represents zero-percent change

The Digital Slide Archive web application was used to manually annotate gray matter of the Emory dataset using web-based image markup tools for drawing. In cases where the gray matter was not easily discernible in the Aβ WSI, a corresponding Bielschowsky silver stain WSI was used to guide the annotation (Fig. 5). Annotations, converted to binary masks, are applied to confidence heatmaps prior to blob detection and new CNN scores are calculated. As expected the cored and diffuse CNN scores increased for most of the comparisons, due to most of the pathologies being observed in the gray matter (Fig. 6). CAA CNN scores in contrast showed a percentage decrease as more CAA occurred in both gray and white matter. All previous CNN score comparisons were recreated using these new scores (Additional file 2: Figure S6-S8). In all but one comparison, the results were similar when using whole tissue CNN scores as opposed to gray matter only CNN scores, with no new significant differences being observed. Additional file 2: Figures S8 showed new statistical significance using gray matter scores between the no/low and intermediate groups for cored CNN scores. Spearman’s rank-order correlations are shown in Table 2 and show similar results when using whole tissue vs gray matter only CNN scores.

**Table 2:** Whole Tissue vs Gray Matter CNN Score Correlation Comparisons

CNN Scores	CERAD-like (Cored)	Reagan (Cored)	CERAD-like (Diffuse)	Reagan (Diffuse)	CERAD-like (CAA)	Reagan (CAA)
Whole Tissue	0.77	0.81	0.74	0.63	0.68	0.43
Gray Matter	0.75	0.83	0.74	0.65	0.66	0.38

Spearman rank-order correlation coefficients between CNN scores and CERAD-like categories and Reagan scores for each Aβ pathology. Correlation coefficients are shown for comparison between whole tissue CNN scores and gray matter CNN scores

### CERAD-style CNN scores

**Fig. 7:**
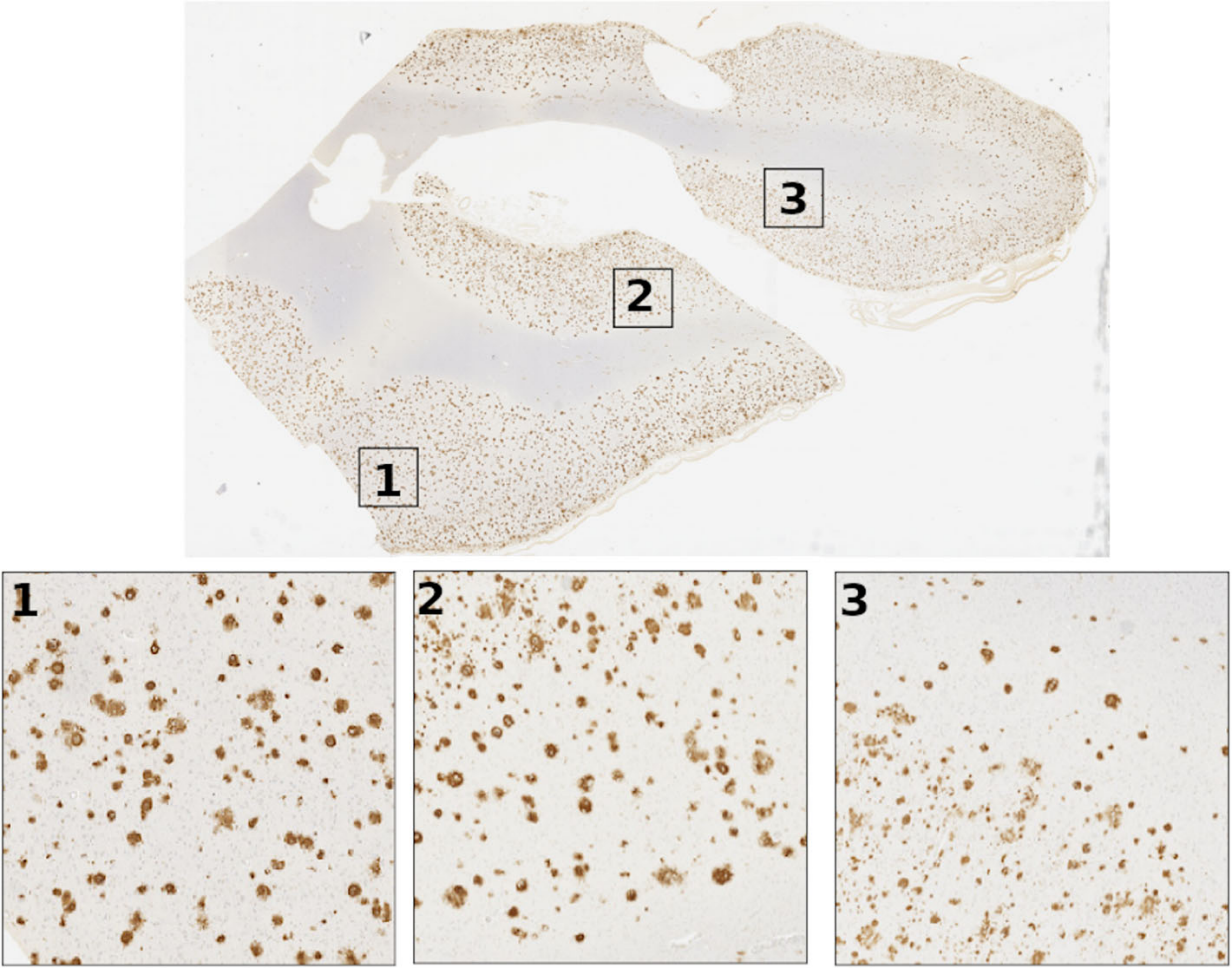
CNN scores analyzed in field-of-view (FOV) style. This example image highlights the three highest FOV CNN scores for cored plaques (numbered boxes with 1 being the highest FOV region). Inserts show the full resolution view of the FOV boxes shown. The size of the boxes represent a similar FOV (10x magnification) used by pathologists in practice when evaluating samples. The FOV boxes were chosen as the three top regions with the highest CNN score that did not overlap

Emory cohort confidence heatmaps were analyzed using a field-of-view (FOV) approach to match the standard practice used by pathologists, which score the slides by the densest pathology region on the tissue (Fig. 7). Following the original paper for CERAD analysis we used an area FOV of 4.0 mm^2^ corresponding to a traditional 10x objective lens used [7]. CNN scores were calculated for all FOV that had at least one pathology present. We identified the highest score FOV and correlated it to the whole tissue score and found it to be highly correlated for all pathologies (Spearman correlation coefficient for cored plaques (0.937), diffuse plaques (0.780), and CAA (0.922)) (Additional file 2: Figure S9).

Grouping the FOV CNN-scores for the single densest region (analogous to traditional CERAD) by their CERAD-like score showed similar differences between the groups as seen when using whole tissue scores (Additional file 2: Figure S9). Spearman correlation coefficients between the CERAD-like categories and the whole tissue CNN scores were good for the Emory data (cored: 0.769, diffuse: 0.735, caa: 0.684). In contrast, using just the highest density FOV for each image resulted in a weaker correlation for cored plaques (0.70) and CAA (0.628), but similar for diffuse plaques (0.746). Cored plaque correlation improved when averaging the top 15 non-overlapping FOV but never quite reached the performance of whole tissue CNN scores. CAA CNN scores saw a decrease in correlation with increasing FOV regions in comparison when using the single highest scoring region (Table 3). Similar results were seen, but with less effect, when comparing to the Reagan NIA criteria scores using the highest FOV vs whole tissue (Table 4).

**Table 3:** FOV Spearman Rank-order Correlation for CERAD-like categories vs CNN scores

num FOV regions	1	3	5	7	9	11	13	15	WT
Cored	0.70	0.72	0.73	0.74	0.74	0.75	0.75	0.75	0.77
Diffuse	0.75	0.74	0.74	0.75	0.75	0.75	0.75	0.75	0.74
CAA	0.63	0.59	0.58	0.60	0.60	0.61	0.60	0.62	0.68

Spearman rank-order correlation coefficients between CNN scores and CERAD-like categories with increasing number of field-of-view regions. *WT* Whole tissue scores

**Table 4:** FOV Spearman Rank-order Correlation for Reagan NIA categories vs CNN scores

num FOV regions	1	3	5	7	9	11	13	15	WT
Cored	0.78	0.79	0.80	0.80	0.80	0.81	0.81	0.81	0.81
Diffuse	0.51	0.50	0.55	0.58	0.59	0.59	0.60	0.60	0.63
CAA	0.38	0.37	0.36	0.37	0.36	0.36	0.35	0.36	0.43

Spearman rank-order correlation coefficients between CNN scores and Regan NIA categories with increasing number of field-of-view regions. *WT* Whole tissue scores

## Discussion

The use of semi-quantitative approaches has been the standard of practice in neuropathology for decades. The introduction of methods such as CERAD, almost 30 years ago, provided a much needed consensus criteria when assessing pathological samples for diagnosis [7, 8, 34]. Since then, the limitations and downsides of these methods have been widely discussed in the literature and many have pursued more robust methods to enhance and improve the current standard [11, 12, 36]. The advent of digital slide scanning technologies and advances in computer vision, driven by improvements in machine learning, can potentially help to overcome the limitations of current scoring systems.

Computational approaches based on machine learning are powerful due to their ability to provide highly accurate results on complicated imaging tasks; the availability of large, well-annotated imaging data sets has been essential to this work. However, the application of these technologies in the medical imaging domain is hampered by the small pool of people qualified to provide expert labels for training data. Unlike famous imaging datasets such as ImageNet [37], which incorporate classes of images such as cats and dogs, the generation of large pathologically-annotated datasets can limit our use of machine learning in the field. The work of Tang et al. was notable because of their creation of a large annotated dataset to classify pathologies at high resolution in WSI.

A well-trained neuropathologist can automatically adjust for differences in brain region, staining intensity, the presence of artifacts (tears, shearing), and aging or fading of slides during their evaluation process. While it is theoretically possible to “teach” a machine learning model to adjust for such variation, if such variation is not present in the training data used for model generation, such factors can cause machine learning models to produce erroneous results. These variations are exacerbated when comparing images across institutions that might not use identical protocols for tissue preparation and staining. The rise of online databanks containing WSIs is still in its infancy but will alleviate some of the variation seen in pathology imaging data, as slides can be digitized proximal to staining and thus artifacts occurring due to slide age will be minimized [10, 28, 38]. Other variations amongst cohorts will remain a challenge, such as stain color variations, cohort inclusion / exclusion criteria, as well as disease heterogeneity. If the aim is to develop computational pipelines to replace or support current methods, they must be clearly shown to be robust to these variations.

In this work we validated a previously published CNN pipeline and were able to not only reproduce the original results on the original data set, but also directly apply the model to a new cohort [17]; and without retraining the model, produce quantitative scores in the Emory data set that strongly correlated with independent CERAD-like scores. Even though the two cohorts showed differences upon high level visual inspection (Additional file 2: Figure S10-S14), the pipeline tested in this work retained its previously published performance when applied to the new cohort. Indeed, performance between the two cohorts was comparable for all three pathologies of interest (Fig. 2). Surprisingly this was true even though the model used to generate the quantitative scores had been solely trained on annotated data from another institution. We noticed that Emory cohort slides showed considerable fading as they had been stained years before. Considering machine learning models perform poorly when the training data poorly represent the population data, it is evident this model is robust enough to account for common pathology slide variations [39]. Of interest in future work would be to train a new model independently on newly stained and annotated Emory cohort images and compare its performance to the original model, as well as extend this work to other cohorts at different institutions, other anatomic areas, and have images annotated by multiple experts.

We were also interested in dissecting this pipeline beyond the original investigation using an Emory cohort selected to contain additional variance. When selecting the Emory cohort we focused on two factors: (1) cases showing a wide range of the three Aβ pathologies of cored and diffuse plaques, and CAA; and (2) cases displaying varied pathological diagnoses (that including concomitant diagnoses). Various neuropathologies often occur together and it is still poorly understood how some of these markers of pathology may interact, and whether there is a clear cause and effect between them [2, 22, 40]. Most of these neuropathological diagnoses have clear criteria, at least within the same institutions, and are often defined by pathologies within select neuroanatomic locations. For example, AD is clearly identified by Aβ and tau pathologies present in the immuno-stained tissue, TDP-43 inclusions are identified on TDP-43 immunohistochemistry and may be localized to limbic areas and / or cortical regions, and Lewy body disease is characterized by the presence and distribution of Lewy bodies identified on alpha-synuclein immunostained tissues and can be located in brainstem, limbic, and/or cortical regions [3, 6, 9, 20, 21, 41, 42]. Our new cohort contained cases that included various categories of concomitant diagnosis (AD + TDP-43, AD + LBD, AD + LBD+ TDP-43) but also cases that showed only AD pathologies and normal control subjects. We want to reiterate that we *only* evaluated temporal lobe staining for Aβ. LBD and TDP-43 pathology are defined by the presence of different pathologies (Lewy bodies and TDP inclusions); while these inclusions may be present in the temporal lobe in some cases, they are best assessed using staining protocols other than Aβ immunohistochemistry. When we grouped AD with concomitant pathologies separately to assess differences between concomitant groups and the control group, these were clearly distinguishable from each other (Fig. 3). Surprisingly the concomitant diagnosis group of AD + TDP-43 showed significantly greater CNN-score for cored plaques than the AD group. Recent studies have demonstrated associations with AD pathologies and TDP-43 deposition and more research is needed to further determine this significance [43].

Another aspect we investigated in this work was comparing pathologies within gray matter compared to the entire tissue section. Most Aβ deposits are located in the neuronal rich gray matter with little seen in the white matter [26]. This notion was borne out in the confidence heatmaps in Tang et al. [17]. Because of this distribution, one might anticipate that variations in white matter-to-gray matter ratio between the images would introduce inherent noise on the CNN scores. Upon restriction of the analysis to gray matter regions, CNN scores remained correlated with CERAD-like categories, Reagan scores, and pathological diagnosis and did not alter statistical comparison amongst disease groups (Table 2 and Additional file 2: Figure S6-S8). Cored and diffuse plaque CNN scores increased when focusing on the gray matter only, with average percent change seen at 23% for cored and 29.3% for diffuse. CAA pathology showed an average decrease in CNN scores in contrast, seen as an average 22.9% *decrease* in score. We hypothesize this is mostly due to similar ratios of white-to-gray matter in the imaging cohorts, but also to the low amount of pathologies that do occur in the white matter.

In human scoring schemes the use of a small field of views, usually the highest density region for the CERAD criteria, can improve human consistency and reliability [7, 44]. Computationally, we could also take a similar approach and only score the images by their highest density regions. However, we find using a larger area to calculate the scores results in better comparisons with human semi-quantitative scores. This is promising as a benefit of using computational approaches is the ability to reliably analyze large regions of images that are simply not scalable for humans. The real potential strength of this capability, however, is not displayed by this simple analysis, as ultimately it must still correlate to categories defined by only one observer. Additional works with multiple annotators are warranted. Analysis focusing strictly on whole-tissue distributions of pathologies, not just a single score per image, might shed new light into pathologically unique groups.

Together the work presented here shows strong evidence of a neuropathology imaging machine learning pipeline robust to cohort variations, however, some limitations exist. Although the model displayed great performance on the new cohort, significant variations were seen in select variables. Specifically for diffuse plaques, the most abundant pathology, we saw large standard deviations between the cohorts and even within cohorts (Fig. 2). Upon close inspection, the CNN algorithm was grouping very dense regions of pathologies together and counting them as one. This is the inherent nature of diffuse plaques. This was unexpected since we used the same trained model as the previous published work. Further investigation revealed color preprocessing had created variations between our re-creation and the original published work due to differing computer package versions, including Python language and operating system versioning. Since the pipeline involves some user-defined parameters, variations in preprocessing can result in unforeseen differences. For better reproducibility, we developed and have made available a Docker container that bundles the specific versions of Python and system packages used in this work (https://hub.docker.com/repository/docker/jvizcar/ab_plaque_box). Future work leveraging this contained environment could expand on the methods used in this pipeline. Of interest would be individual models that can focus on different pathologies of interest, such as TDP43 and LBD. This would allow a deeper phenotyping for cases by analyzing multiple stains (as only 4G8 was used) and uniquely stratifying concomitant pathologies. As stated previously additional studies examining other brain regions, staining modalities, and having datasets from multiple experts are warranted.

Another route to improving the CNN scoring might be by switching the analysis to a segmentation problem, which would result in a tighter delineation of the pathologies. The biggest hurdle for this would be in generating sufficient training data to achieve high accuracy in a segmentation machine learning model. However, the benefits of this would be vast as it would allow an even deeper phenotyping of pathology from simple burden scores to distribution populations and morphology subtypes within the pathologies. Ultimately, it would allow machine learning models such as the one used in this work to provide not just re-creation of neuropathology assessment but also a means to investigate complex patterns not feasible in purely human-based analysis. We encourage the use of this pipeline and all the provided tools (Docker container, Emory cohort, and code used in this project is made fully available, see Data Availability) to further investigate the benefits that it could have in common neuropathology practice. Furthermore, we hope this work inspires other research groups to establish collaborations with other institutions to validate machine learning models in pathology in diverse and larger cohorts.

## Conclusions

We demonstrate a previously published machine learning model used to generate quantitative scores for Alzheimer’s disease Aβ burden performs well on a new and varied cohort. We show minimal modifications are required to achieve similar results and the model is more robust than previously explored, as it also stratifies clearly between NIA Reagan criteria scores and pathological diagnosis. Further investigation showed gray matter segmentation was not needed to achieve equivalent results and single FOV scores did not perform as well as using whole tissue. This work supports the idea that machine learning can be successful in multi-institutional pathological datasets and is a critical step to show that machine learning can be used to support pathological practice.

## Supplementary information

**Additional file 1.** Emory cohort case information, demographics, CERAD scores, pathology diagnosis, Reagan scores, post-mortem interval, CERAD score, Braak stage, Thal stage, ABC score.

**Additional file 2:** Supplementary figures. **Figure S1.** Recreation of Receiver operating curves and precision recall curves for validation dataset and test dataset. **Figure S2.** comparison between Emory and Tang dataset grouped by CERAD-like scores. **Figure S3.** combined results of Tang & Emory datasets grouped by CERAD-like scores. **Figure S4.** pathological diagnosis CNN scores comparison when including 2 cases with cognitive normal diagnosis but AD pathology. **Figure S5.** CAA CNN score grouped by pathological diagnosis. **Figure S6.** Gray matter CNN scores grouped by CERAD-like scores for Emory dataset. **Figure S7.** Gray Matter CNN scores grouped by pathological diagnosis (Emory cohort). **Figure S8.** Gray Matter CNN score grouped by Reagan criteria score for Emory data. **Figure S9.** Correlations between whole tissue CNN scores vs highest density FOV score. **Figure S10.** Tinctorial differences between slides between the two institutions. **Figure S11.** Low-res images of the Tang train dataset. **Figure S12.** Low-res images of the Tang hold-out dataset. **Figure S13.** Low-res images of the Emory dataset. **Figure S14.** High resolution sample images for the Emory dataset, Tang train and holdout datasets.

## Data Availability

The datasets analysed during the current study are freely available for download in the Digital Slide Archive instance located at http://computablebrain.emory.edu:8080/#collection/5d607ae8d1dbc700dde750a7/folder/5e29ef629f68993bf1676f78. The code used to run the analysis and generate the publication figures can be found at https://github.com/gutmanlab/Emory_Plaquebox_Paper.

## Abbreviations

AD: Alzheimer’s Disease (AD)
LBD: Lewy body disease
TDP: TDP-43 positive inclusions
CERAD: The Consortium to Establish a Registry for Alzheimer’s Disease
WSI: Whole-slide imaging
CNN: Convolutional neural networks
CAA: Cerebral amyloid angiopathy
Aβ: Amyloid beta
FOV: Field of view
