# Supplementary material for: Validation of machine learning models to detect amyloid pathologies across institutions
Source: Acta Neuropathol Commun. 2020 Apr 28;8:59. doi: 10.1186/s40478-020-00927-4 (PMC7189549; doi:10.1186/s40478-020-00927-4)
Supplement: Supplementary file 2 — Additional file 2: Supplementary figures. Figure S1. Recreation of Receiver operating curves and precision recall curves for validation dataset and test dataset. Figure S2. comparison between Emory and Tang dataset grouped by CERAD-like scores. Figure S3. combined results of Tang & Emory datasets grouped by CERAD-like scores. Figure S4. pathological diagnosis CNN scores comparison when including 2 cases with cognitive normal diagnosis but AD pathology. Figure S5. CAA CNN score grouped by pathological diagnosis. Figure S6. Gray matter CNN scores grouped by CERAD-like scores for Emory dataset. Figure S7. Gray Matter CNN scores grouped by pathological diagnosis (Emory cohort). Figure S8. Gray Matter CNN score grouped by Reagan criteria score for Emory data. Figure S9. Correlations between whole tissue CNN scores vs highest density FOV score. Figure S10. Tinctorial differences between slides between the two institutions. Figure S11. Low-res images of the Tang train dataset. Figure S12. Low-res images of the Tang hold-out dataset. Figure S13. Low-res images of the Emory dataset. Figure S14. High resolution sample images for the Emory dataset, Tang train and holdout datasets. [file 40478_2020_927_MOESM2_ESM.docx]

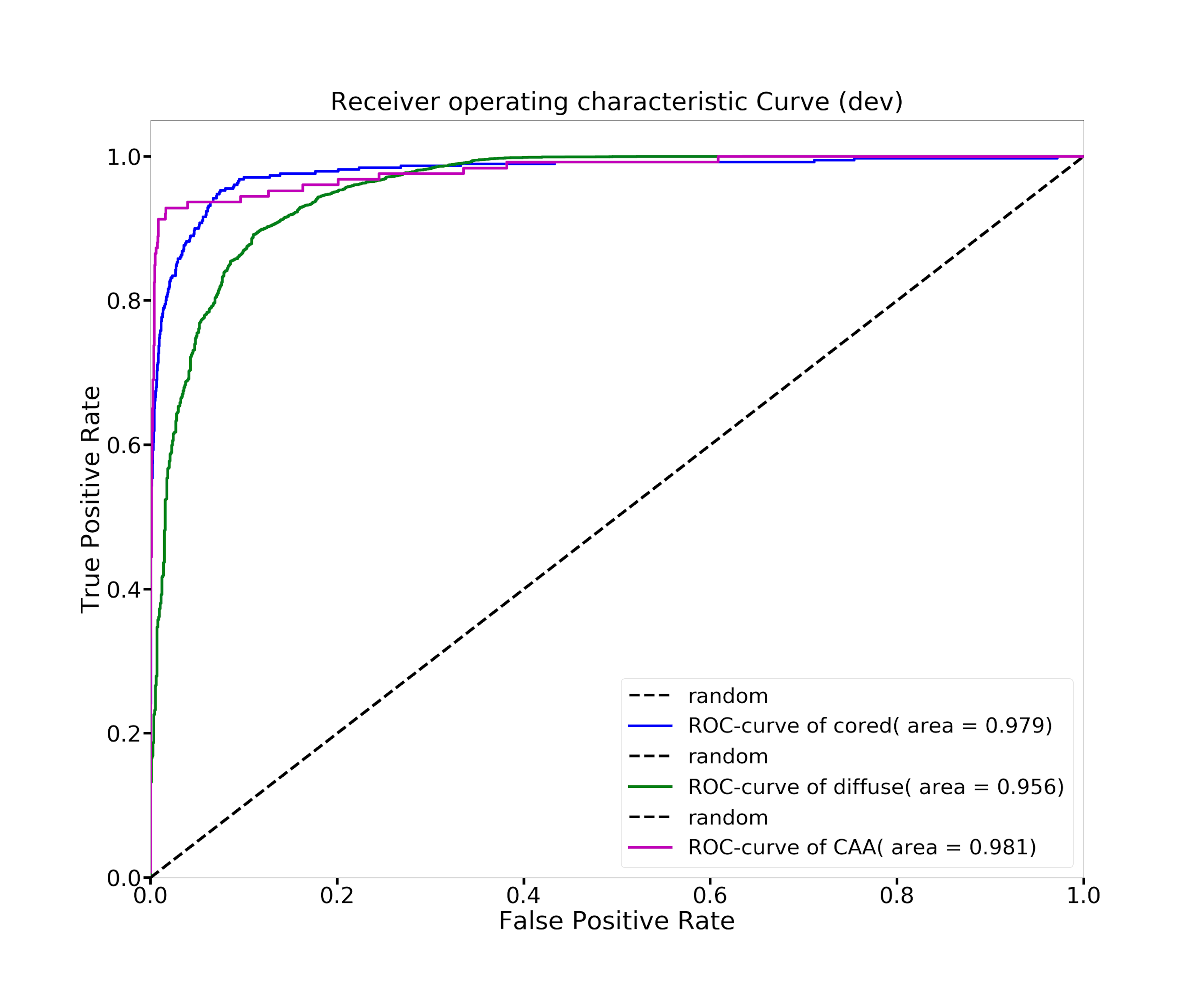

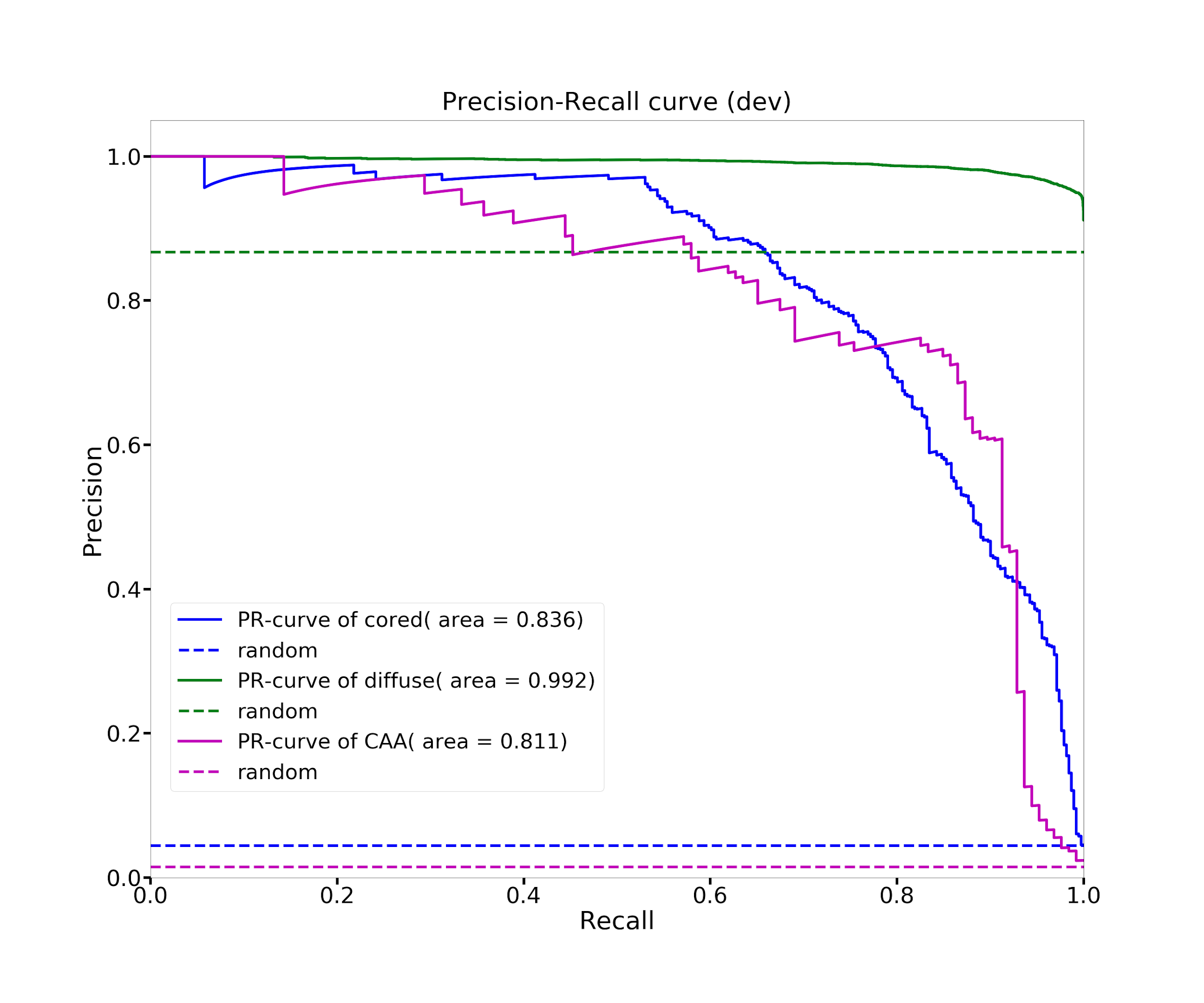


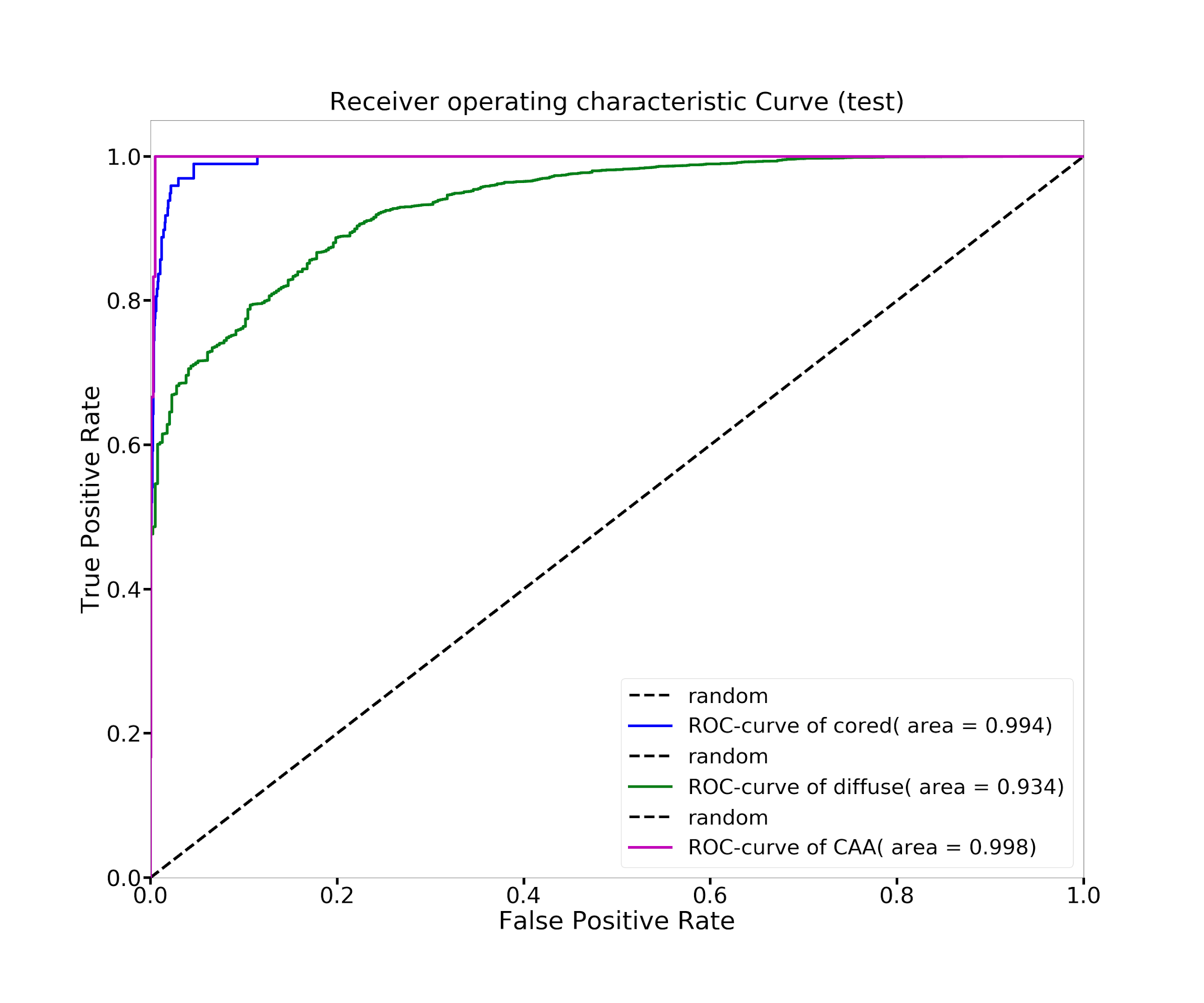

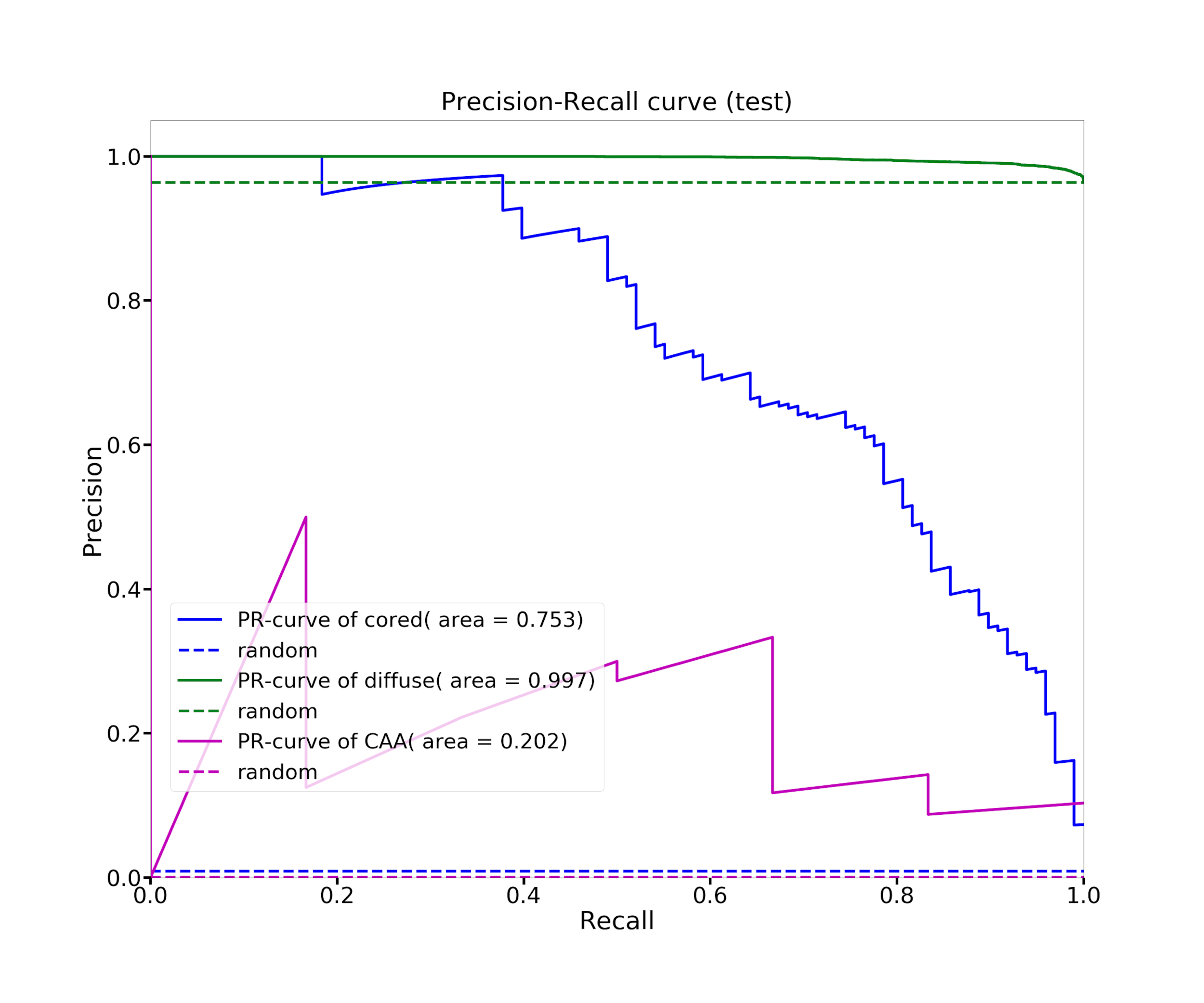


**Supplementary Fig. 1. Recreation of Receiver operating curves and precision recall curves for validation dataset and test dataset.** Top row is the plots for the validation dataset, bottom row for test dataset. Area under the curves for the plots are provided in the legends. Random chance lines are shown as dashed lines. These plots were generated by training a new model with the same code provided by Tang et al and the same training images, tested on the same testing images.

**
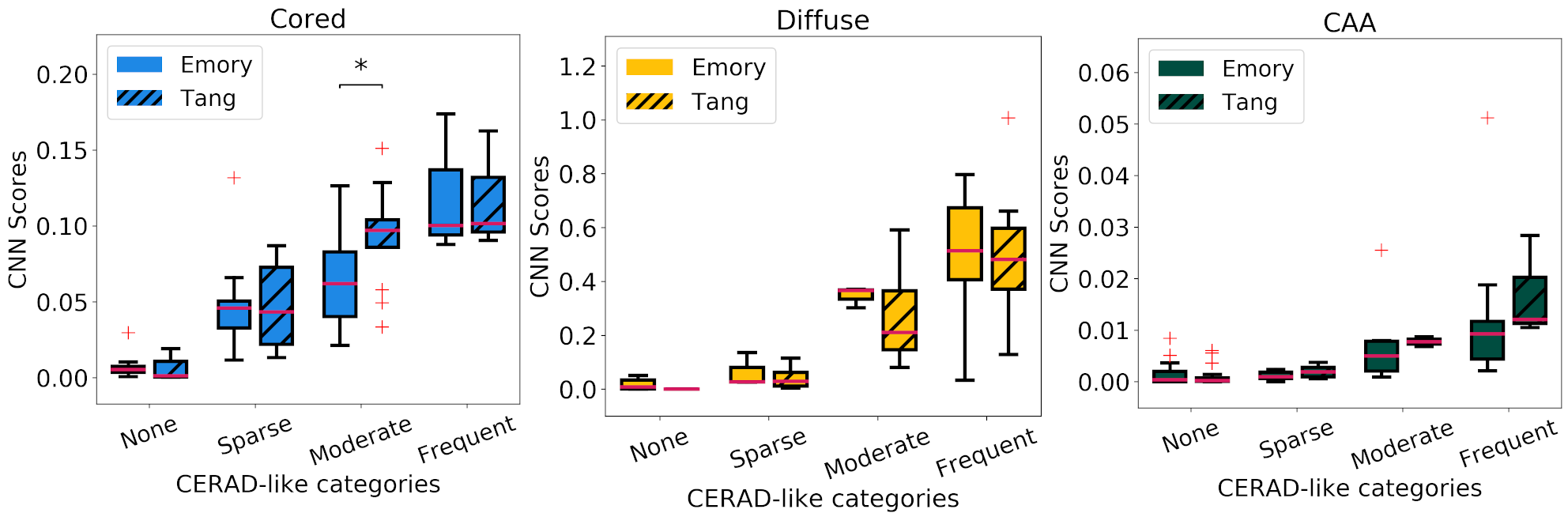
**

**Supplementary Fig. 2. Emory vs Tang dataset CNN score comparisons grouped by CERAD-like categories.** Cases grouped by their CERAD-like categories and CNN scores for both datasets (Tang n=30, Emory n=40) plotted next to each other for comparison. Side-by-side comparisons were assessed using a two-sided independent t-test for the null hypothesis that the means were equal between the datasets. Significant p-values are shown as asterisk with * representing significance with a p-value of 0.05. Whiskers show the interquartile range of +/- 1.5*IQR. Outliers are shown as red + and medians are shown as horizontal red lines in the boxplots. Hashed boxplots represent the Tang data and solid boxplots Emory.

**
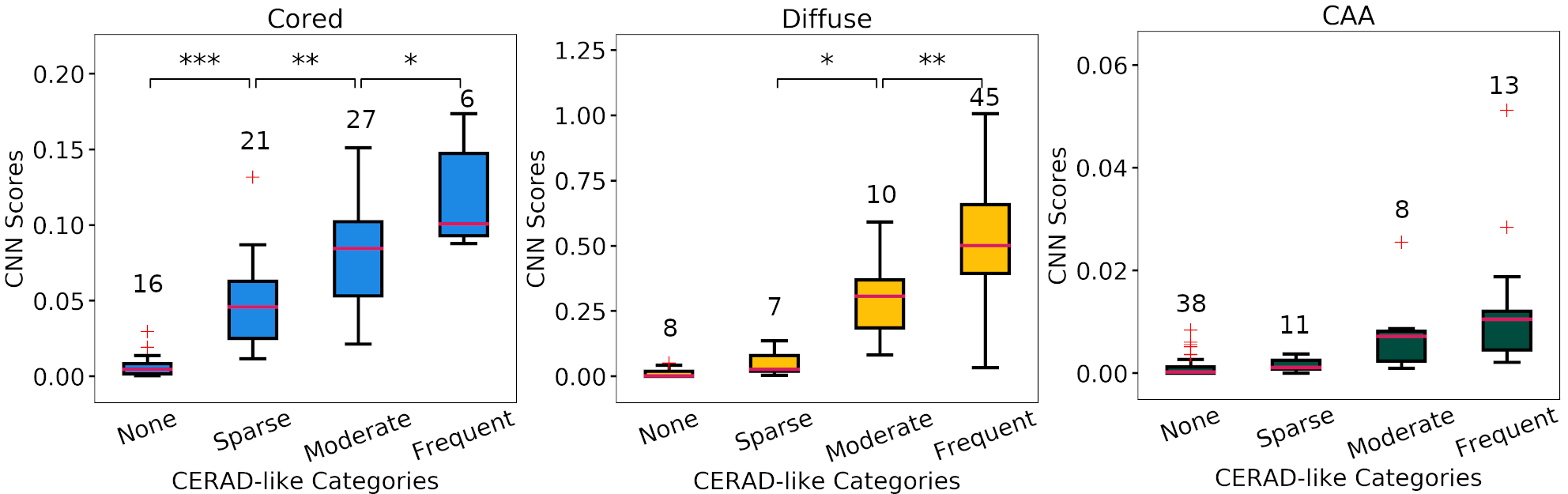
**

**Supplementary Fig. 3. Combined Emory and Tang dataset comparison of whole tissue CNN scores by CERAD-like categories.** CNN scores for the combined datasets (Emory n=40 and Tang n=30) generated from confidence heatmaps grouped by CERAD-like categories. Left boxplot shows comparison for cored, middle for diffuse, and right for CAA pathologies. An ANOVA with post-hoc analysis using Tukey’s test was used to assess significance with an alpha value of 0.05, significance is shown between groups with * for p-value less than 0.05, ** less than 0.01, *** less than 0.001, and **** less than 0.0001. Whiskers show the interquartile range of +/- 1.5*IQR. Outliers are shown as red + and medians are shown as horizontal red lines in the boxplots.

**
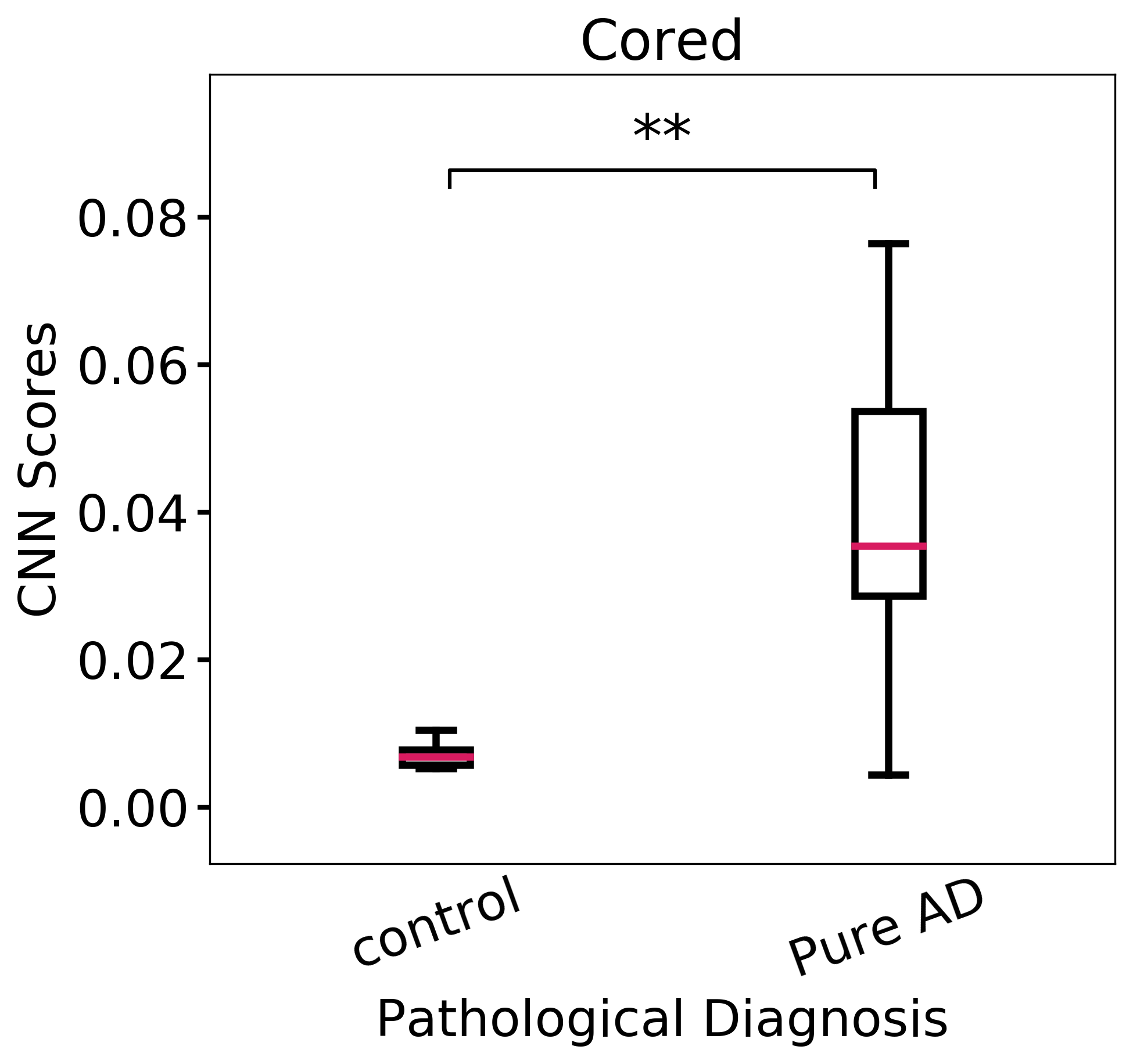

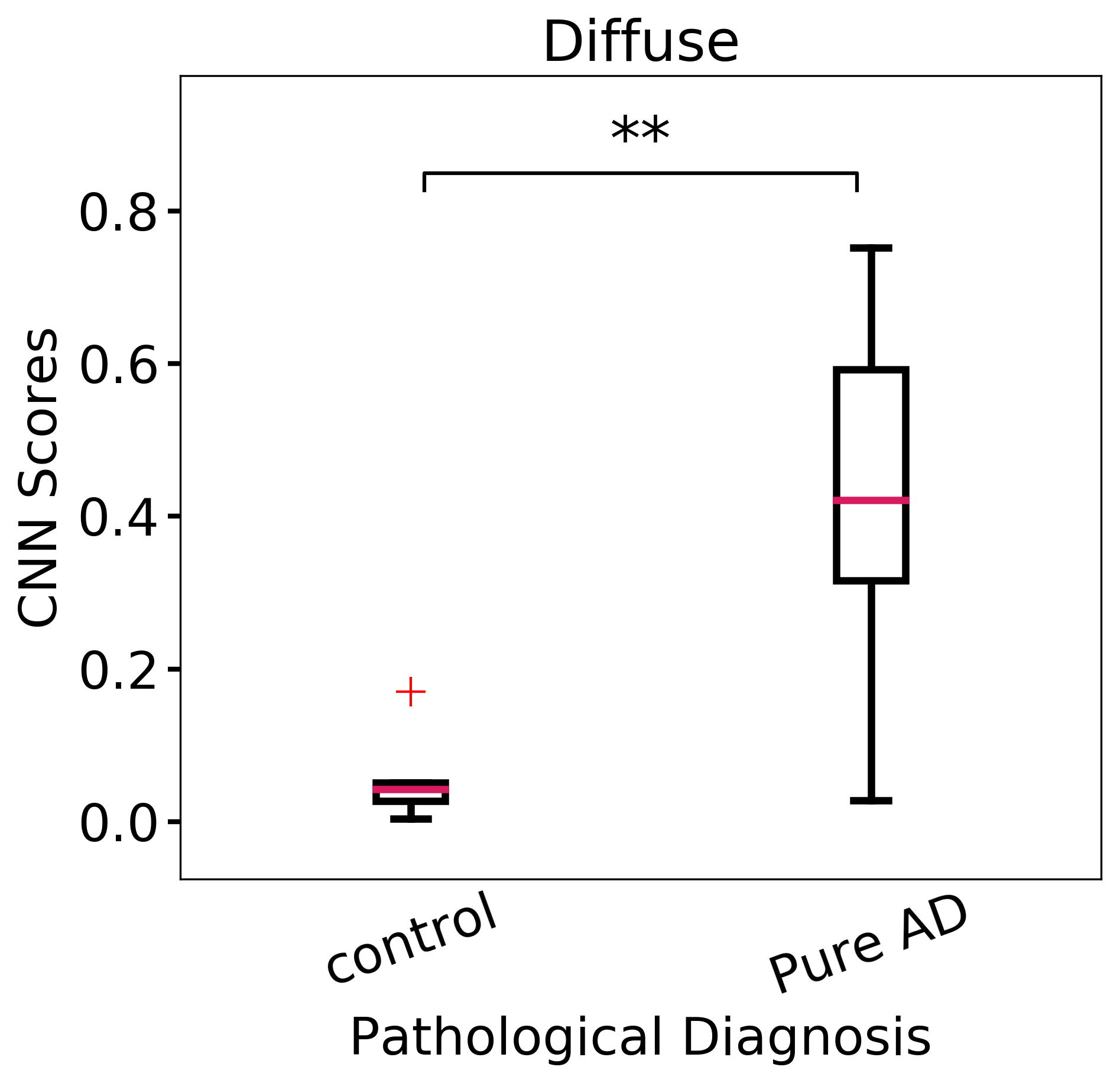

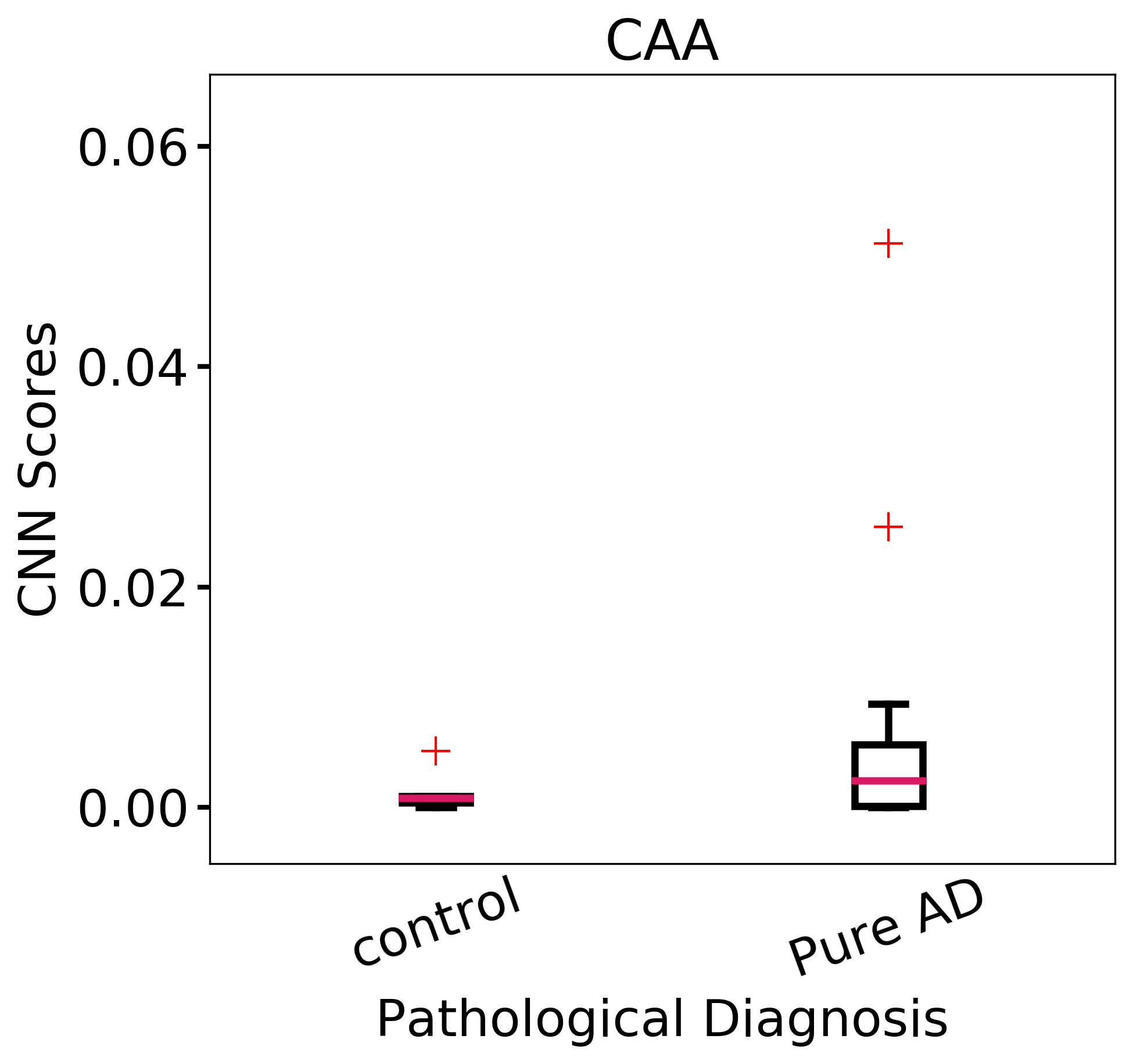
**

**
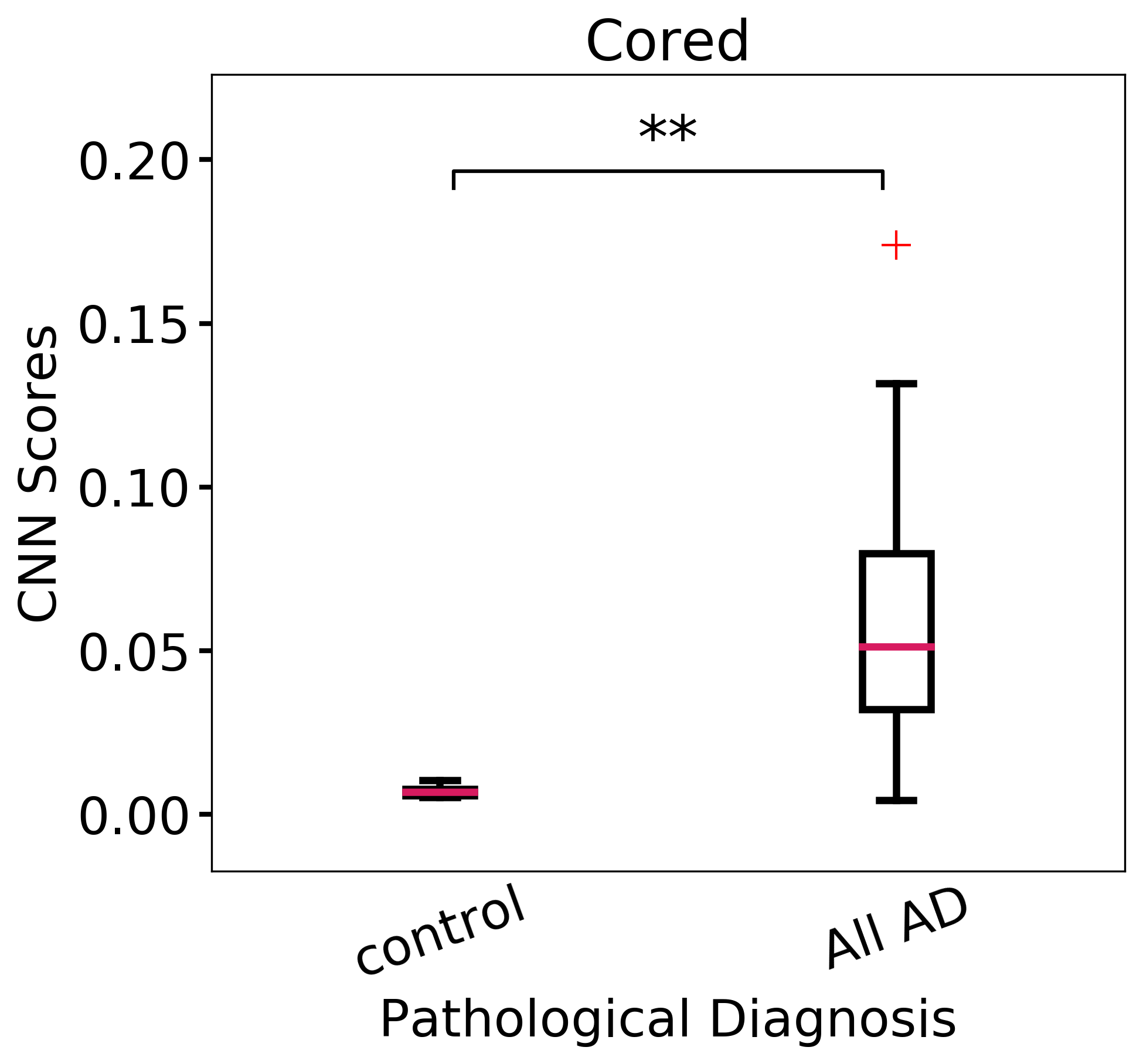

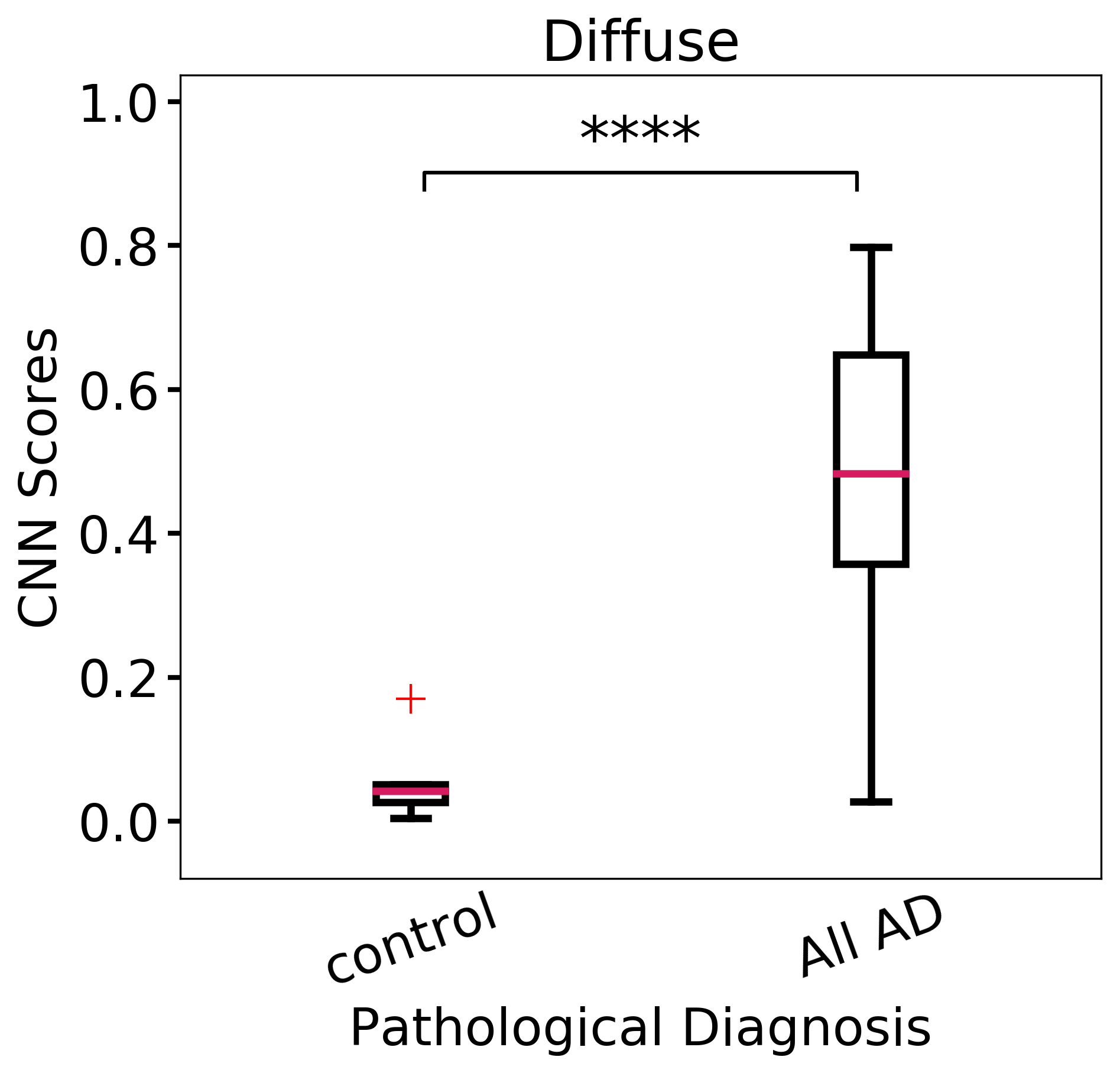

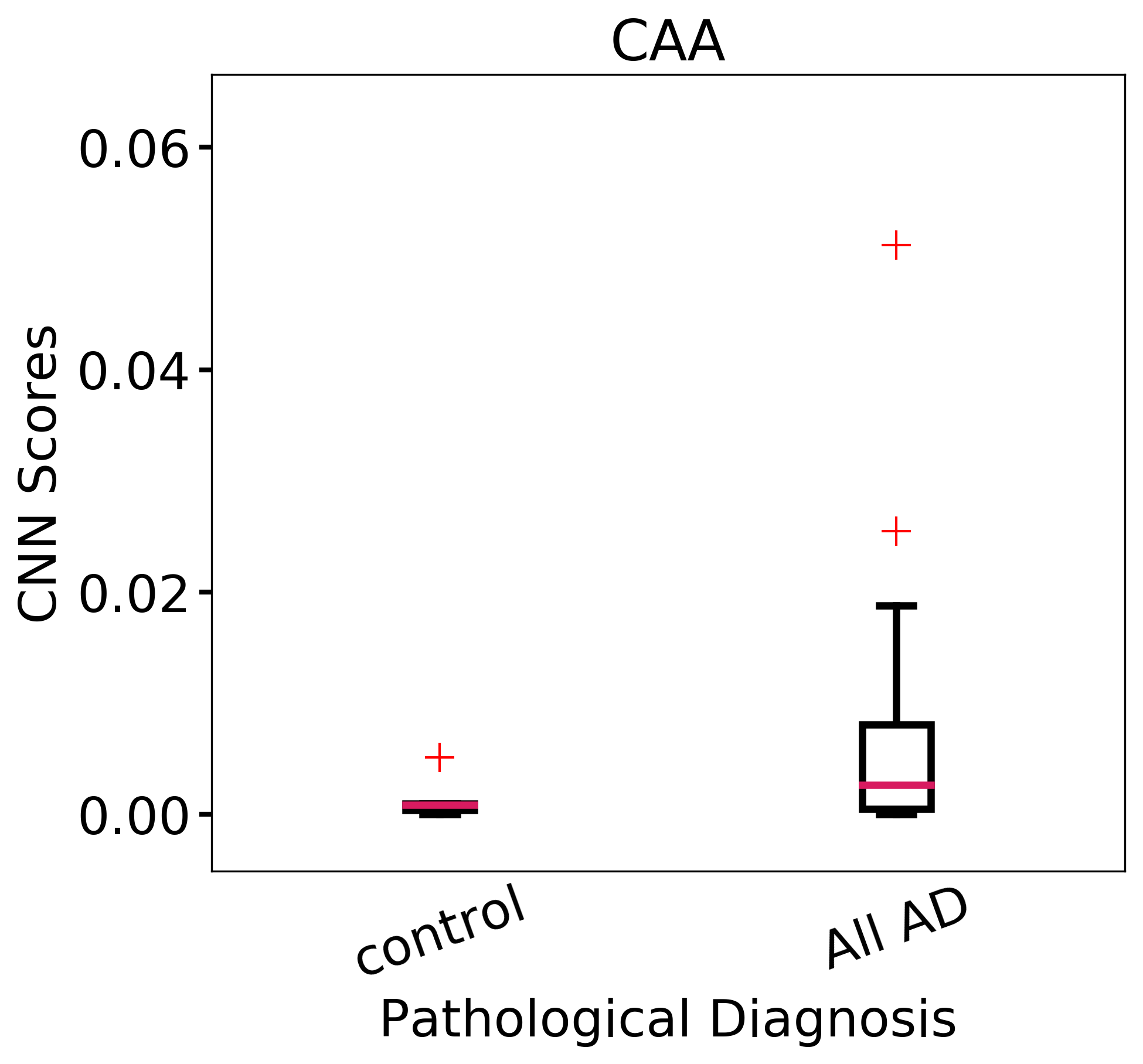
**

**
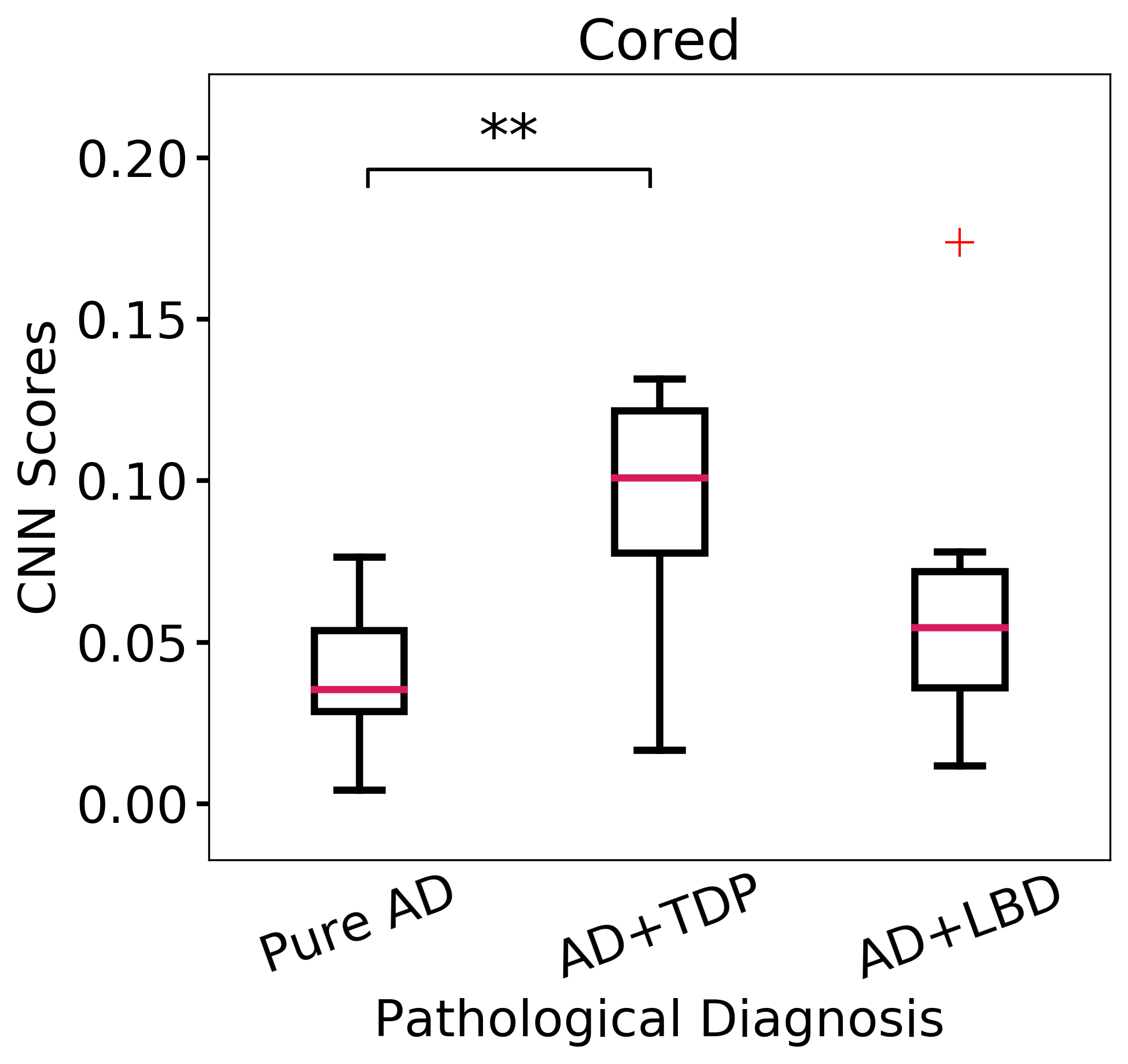

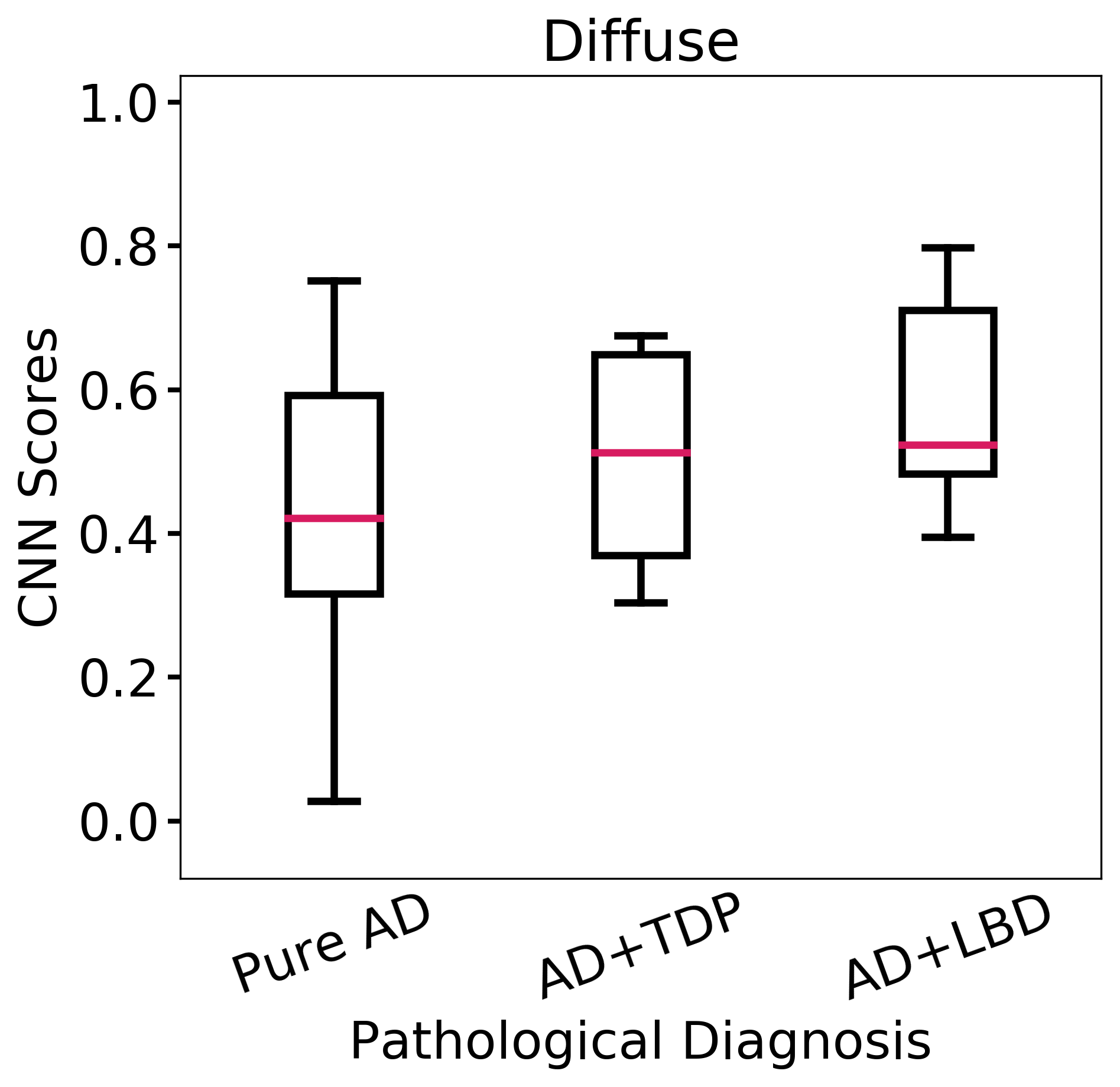

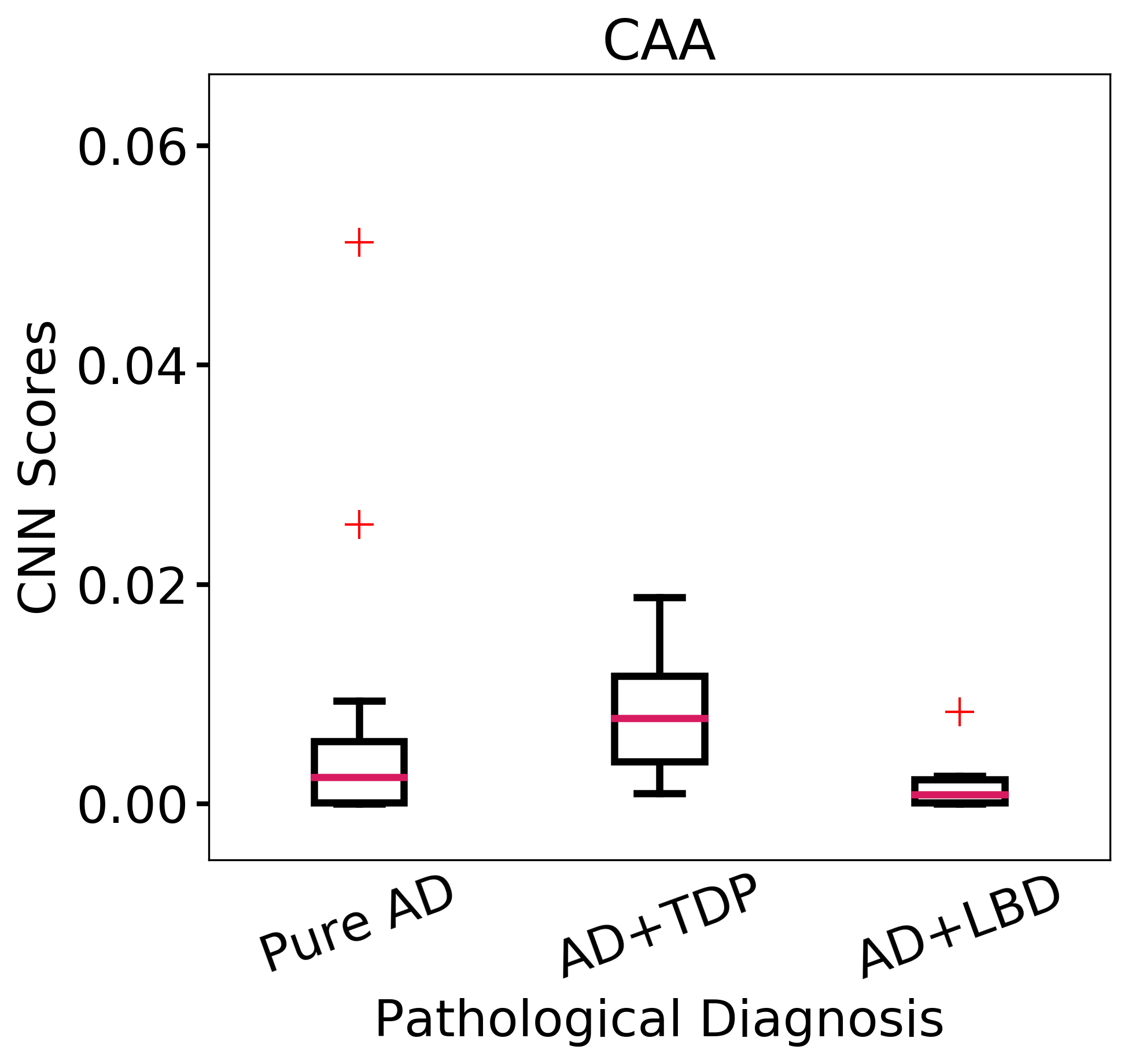
**

**Supplementary Fig. 4. Pathological diagnosis comparisons including the 2 cases with clinically cognitive normal diagnosis but AD pathology present.** Whole Tissue CNN scores grouped by pathological diagnosis (Emory cohort). CNN scores generated from confidence heatmap processing are grouped into three distinct groups: control cases, pure AD (no secondary diagnosis), and all AD (pure AD + cases with secondary diagnosis of TDP and/or LBD). The all AD group is further divided into pure AD, AD+LBD, and AD+TDP ((c) and (f)). Top row shows the comparison between control vs pure AD group. Middle row is the comparison between control and all AD groups. Bottom row is the multi-comparison between pure AD, AD+LBD, and AD+TDP. For the control vs pure AD and control vs all AD comparison a student’s 2-sided independent sample t-test was used to assess significance. For multi-group comparison (bottom row) an ANOVA with post-hoc analysis using Tukey’s test for multiple comparisons was used to assess significance. Alpha value of 0.05, significance is shown between groups with * for p-value less than 0.05, ** less than 0.01, *** less than 0.001, and **** less than 0.0001. Whiskers show the interquartile range of +/- 1.5*IQR. Outliers are shown as red + and medians are shown as horizontal red lines in the boxplots. Control group (n=5), pure AD (n=16), all AD (n=32), AD+TDP (n=8), and AD+LBD (n=7).


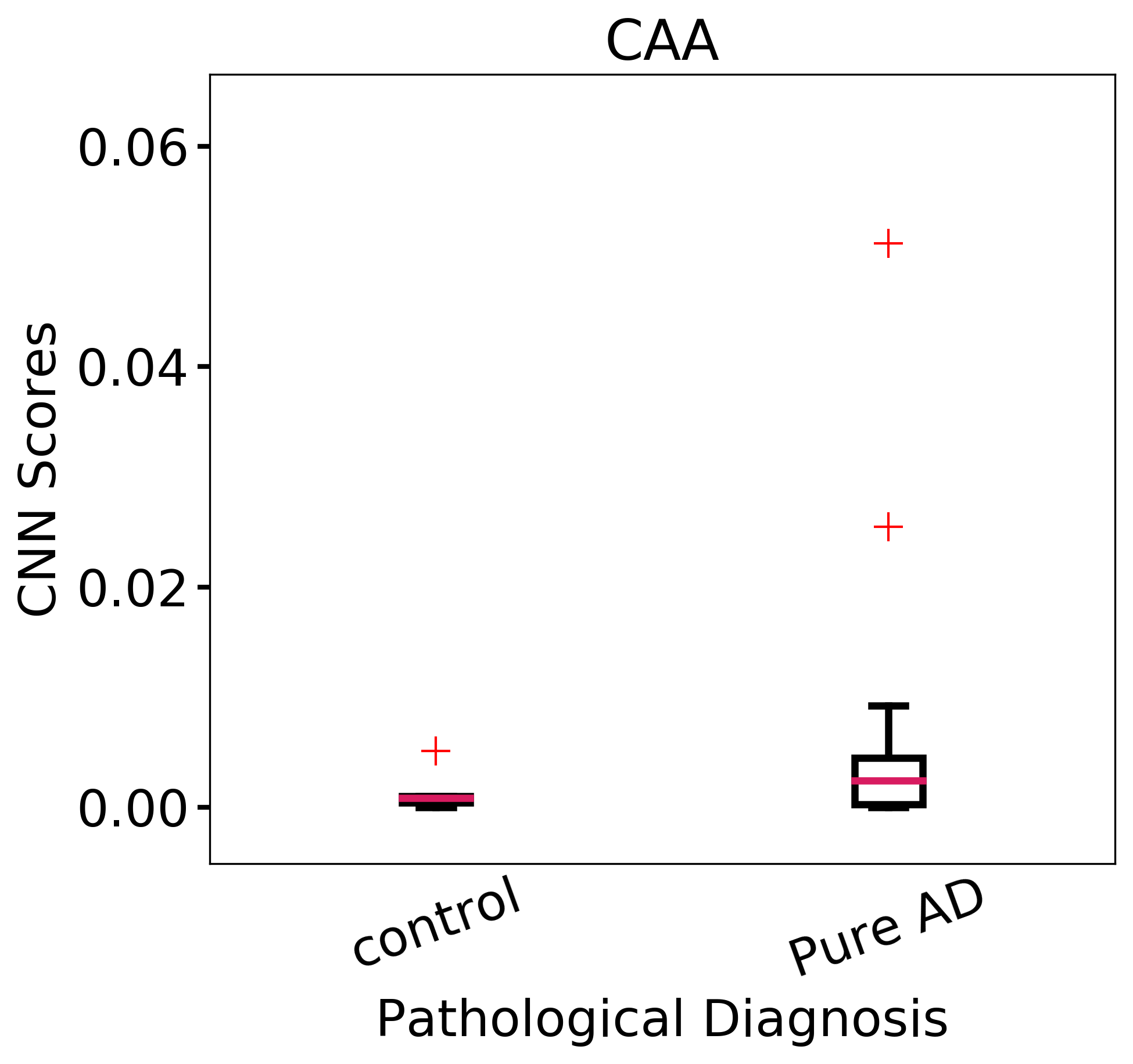

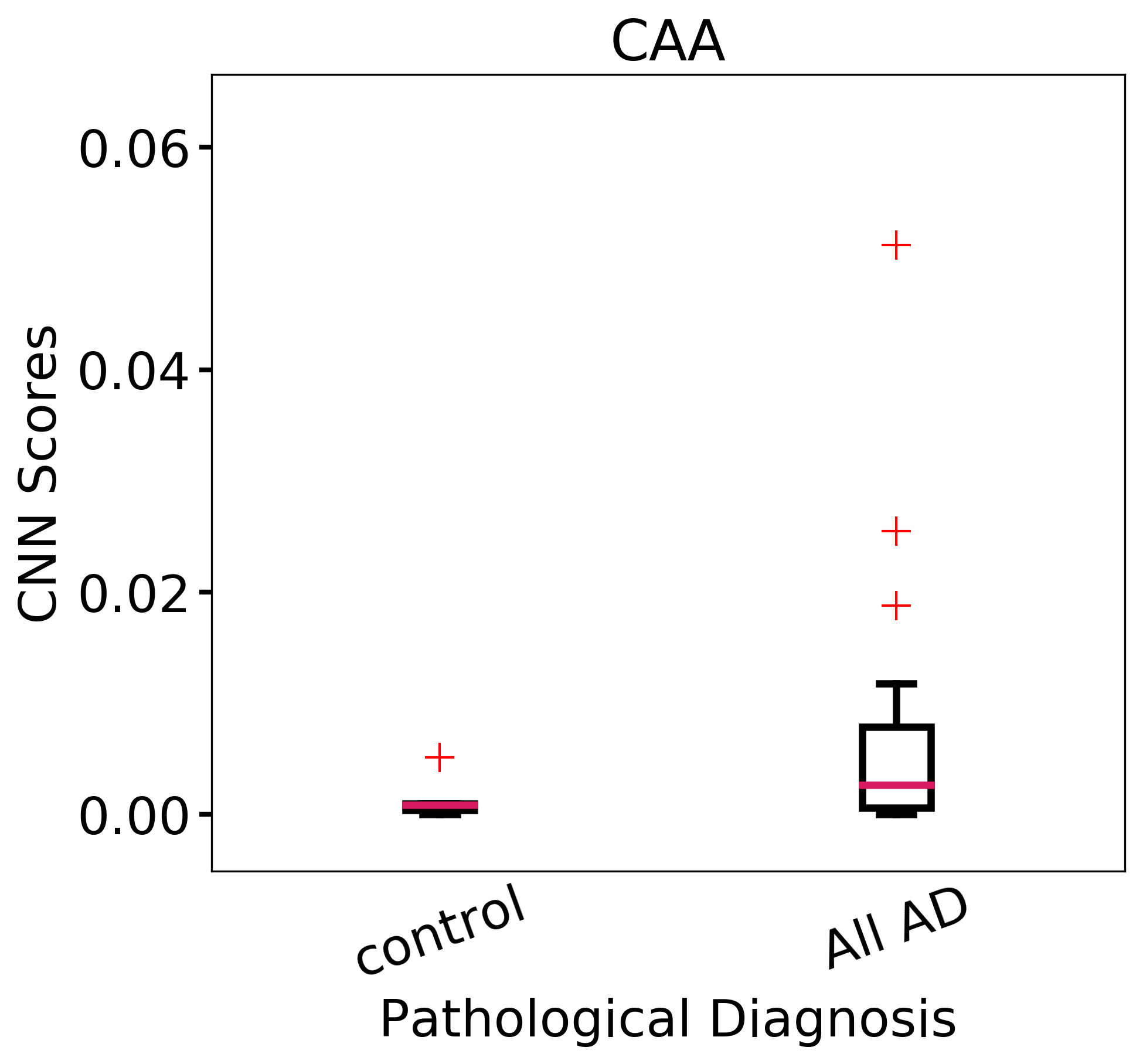

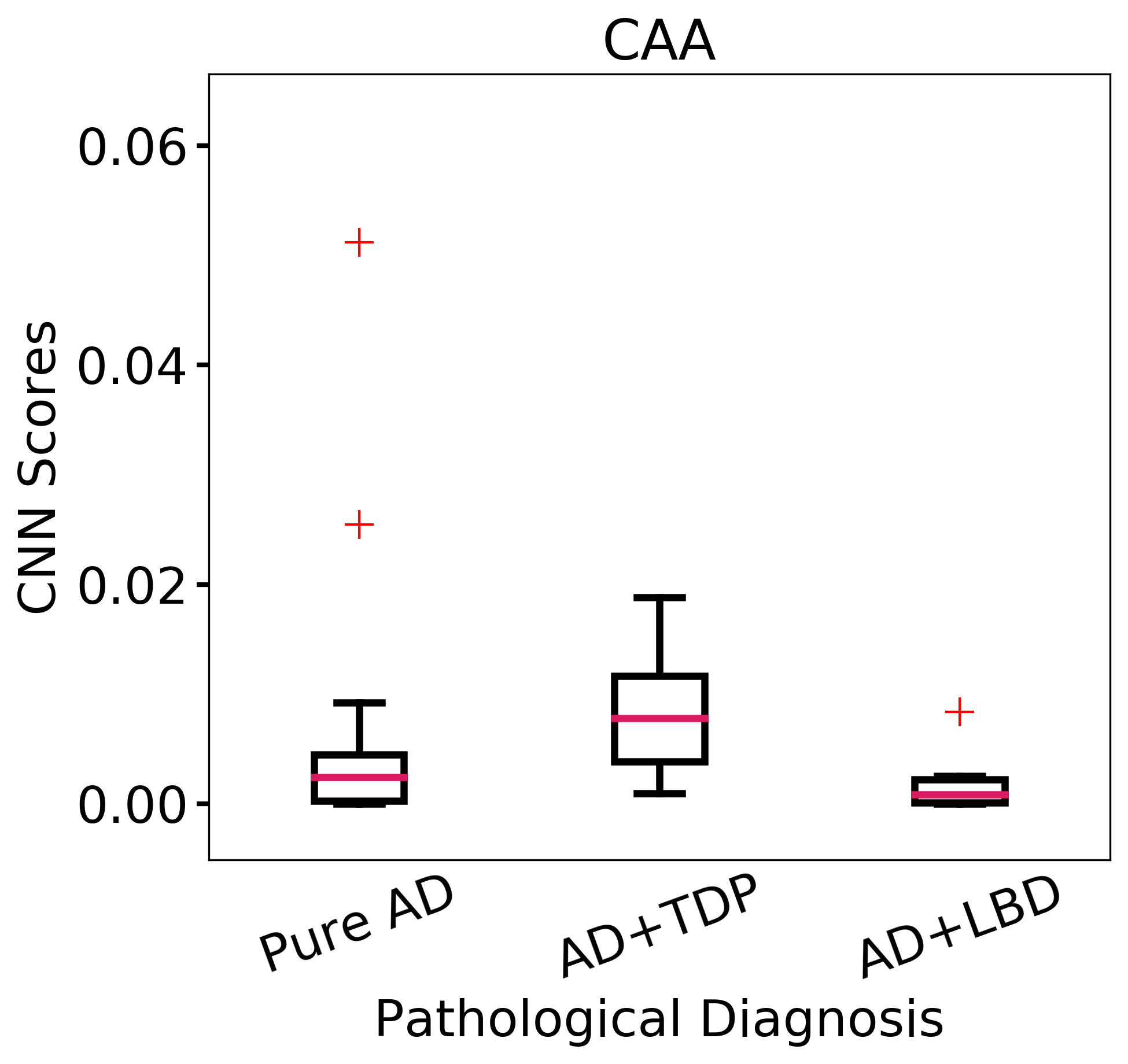


**Supplementary Fig. 5. Pathological diagnosis comparisons for CAA CNN scores.** Whole Tissue CAA CNN scores grouped by pathological diagnosis (Emory cohort). CNN scores generated from confidence heatmap processing are grouped into three distinct groups: control cases, pure AD (no secondary diagnosis), and all AD (pure AD + cases with secondary diagnosis of TDP and/or LBD). The all AD group is further divided into pure AD, AD+LBD, and AD+TDP ((c) and (f)). For the control vs pure AD and control vs all AD comparison a student’s 2-sided independent sample t-test was used to assess significance. For multi-group comparison (right image) an ANOVA with post-hoc analysis using Tukey’s test for multiple comparisons was used to assess significance. Alpha value of 0.05. Whiskers show the interquartile range of +/- 1.5*IQR. Outliers are shown as red + and medians are shown as horizontal red lines in the boxplots. Control group (n=5), pure AD (n=14), all AD (n=30), AD+TDP (n=8), and AD+LBD (n=7).

**
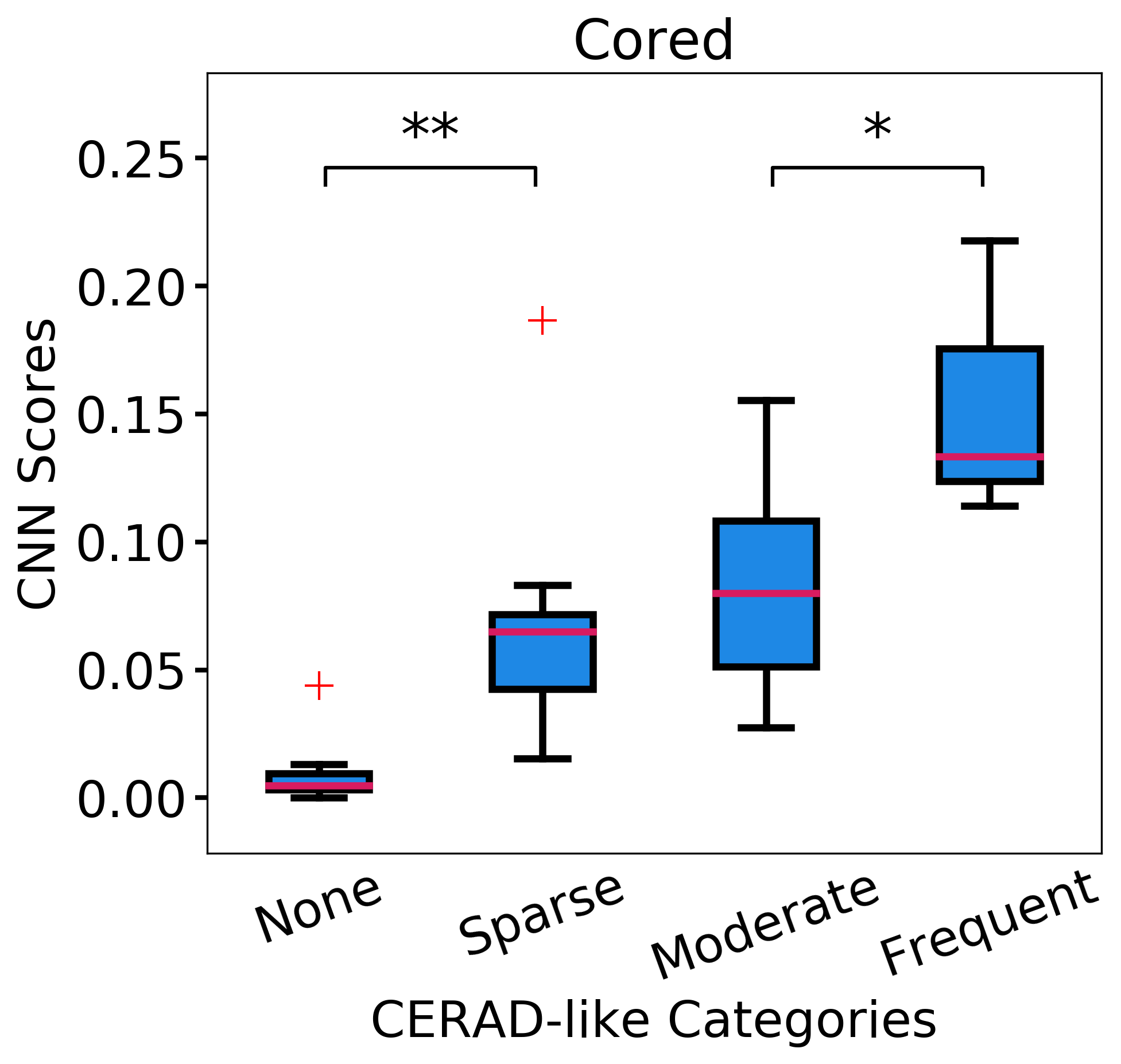

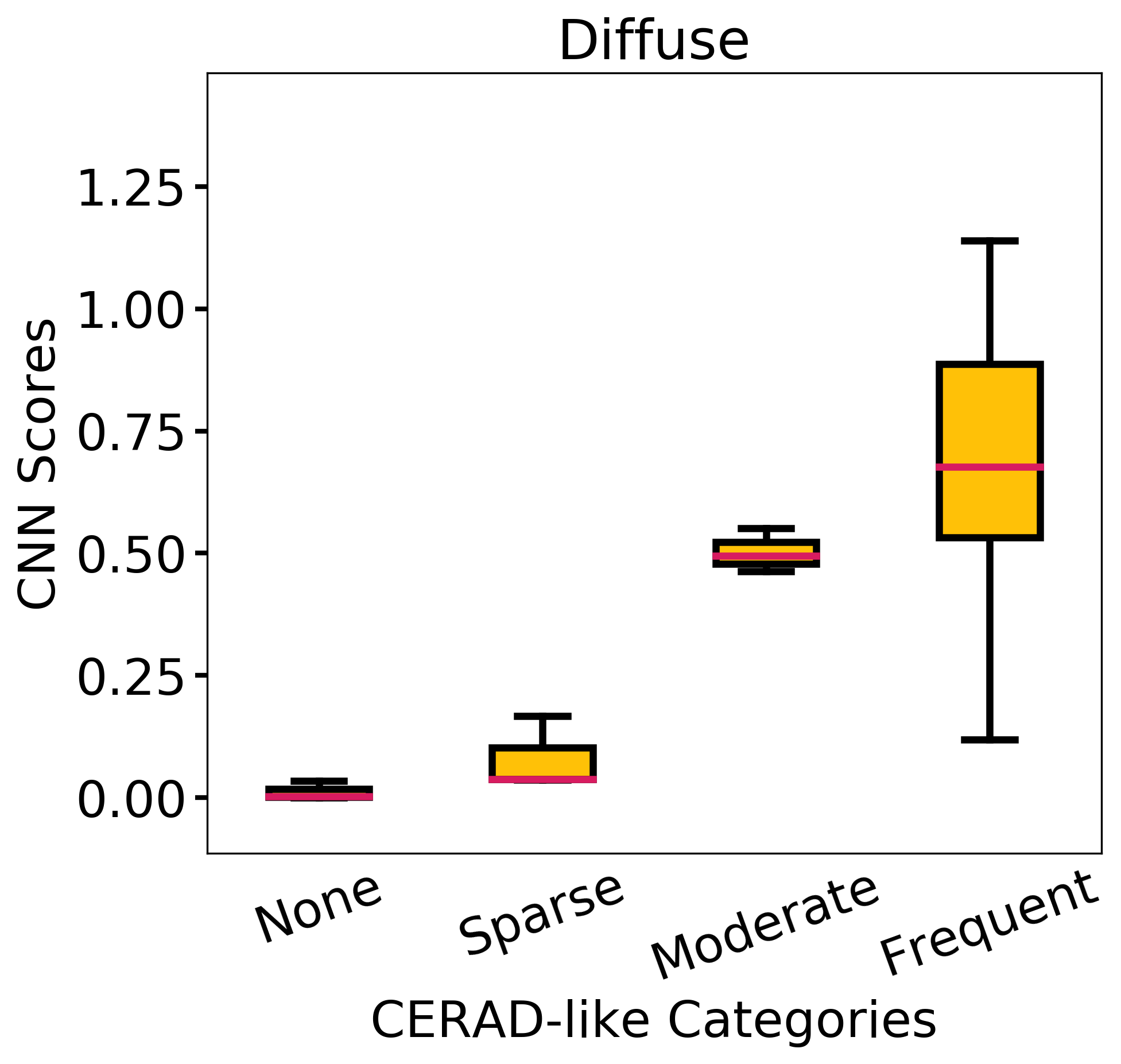

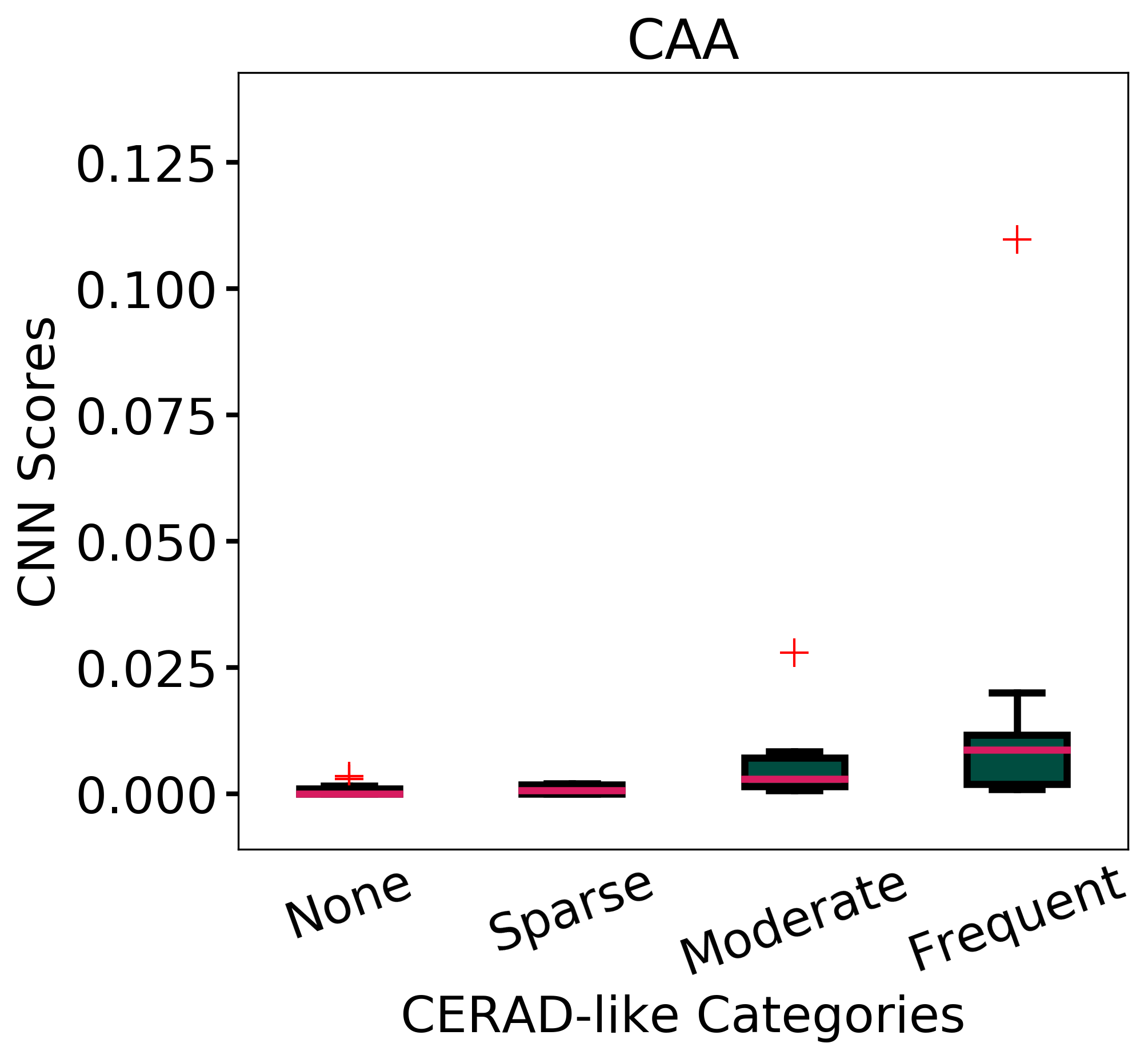
**

**Supplementary Fig. 6. Gray matter CNN scores grouped by CERAD-like scores for Emory dataset.** CNN scores generated from confidence heatmap processing are grouped together by CERAD-like scores for each plaque and statistically compared using an ANOVA with Tukey’s test for multiple comparisons for post-hoc testing.. Outliers are shown with red + and significance between groups is shown (* 0.05, ** 0.01, *** 0.001).


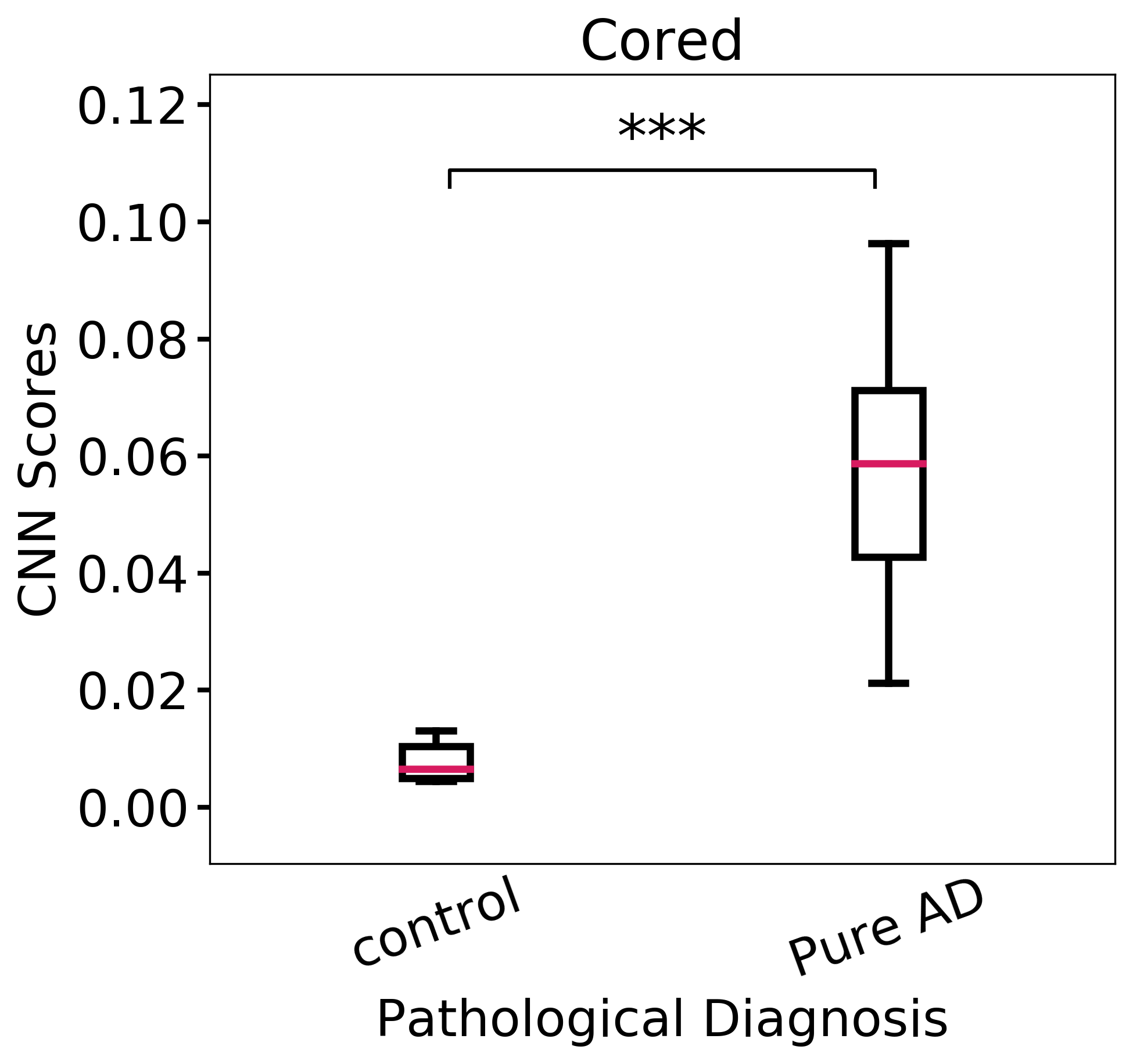

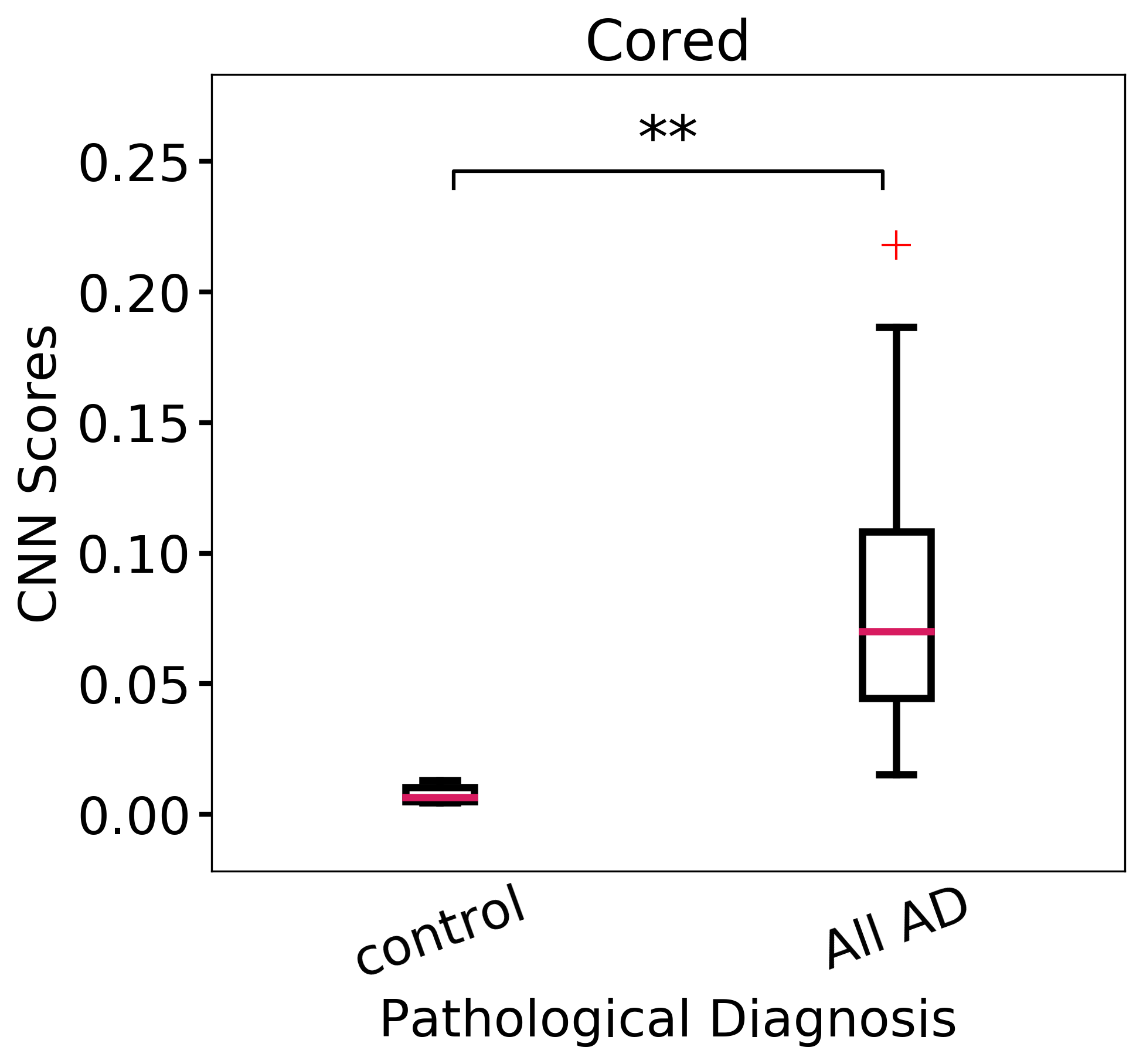

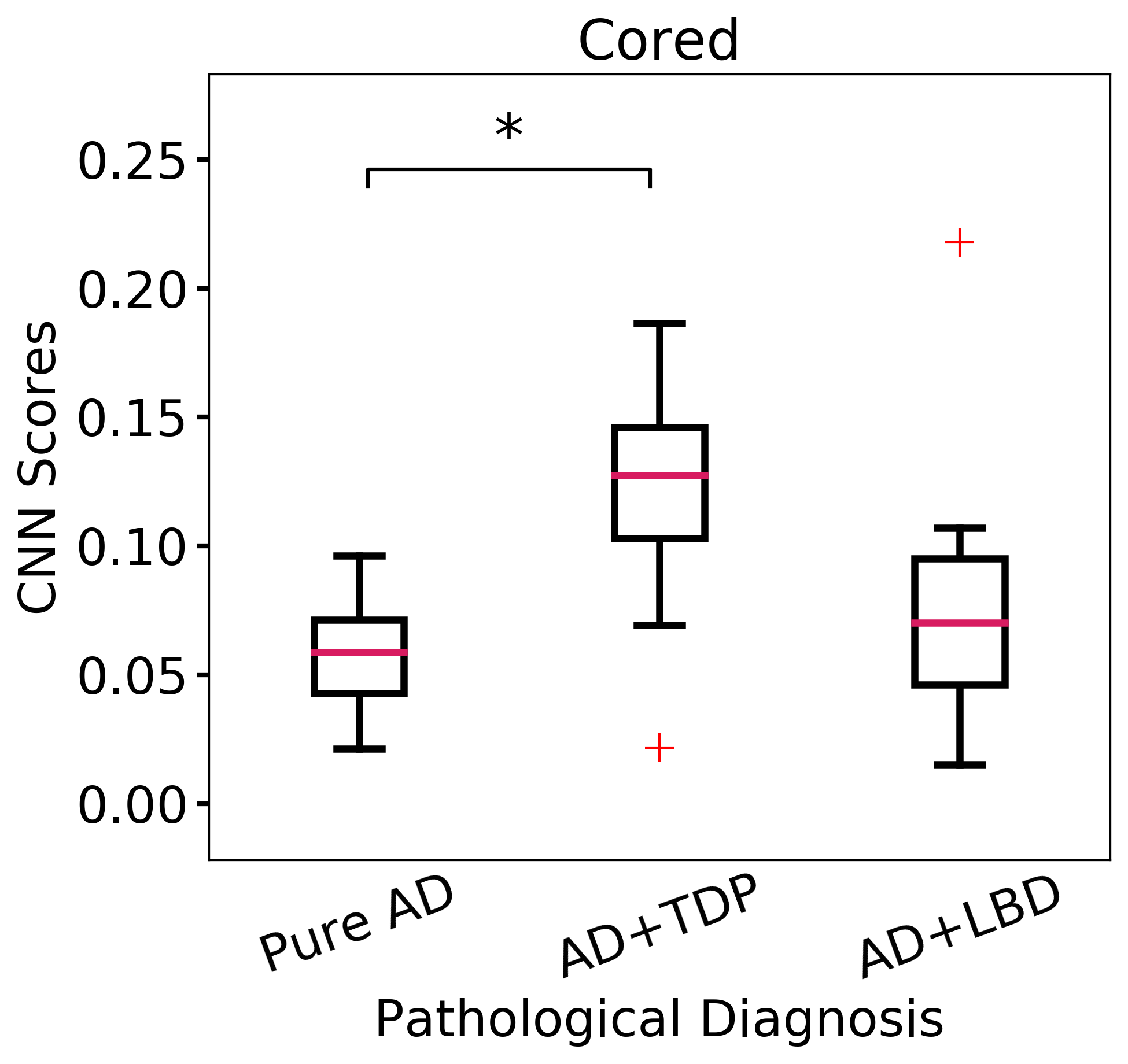


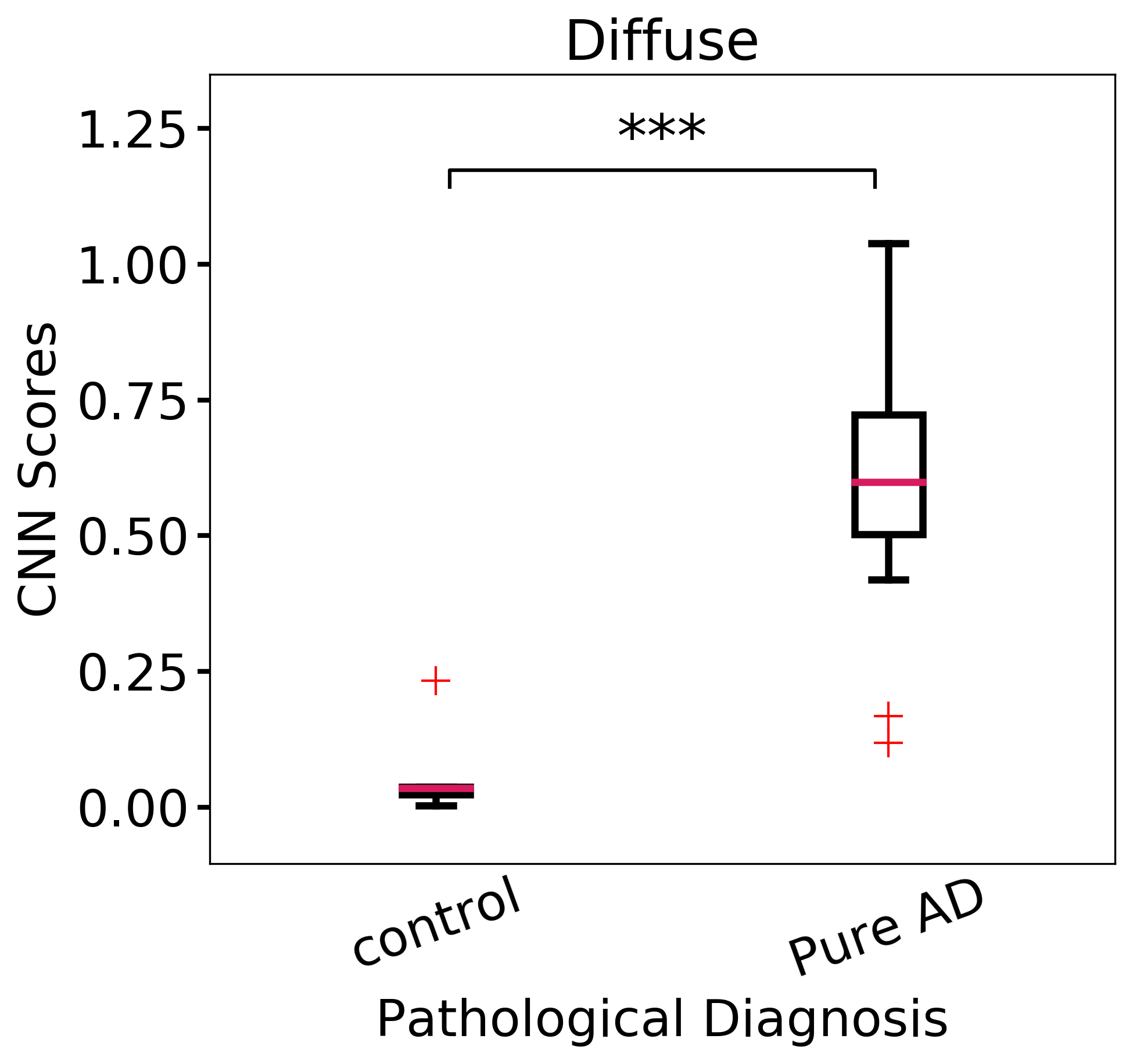

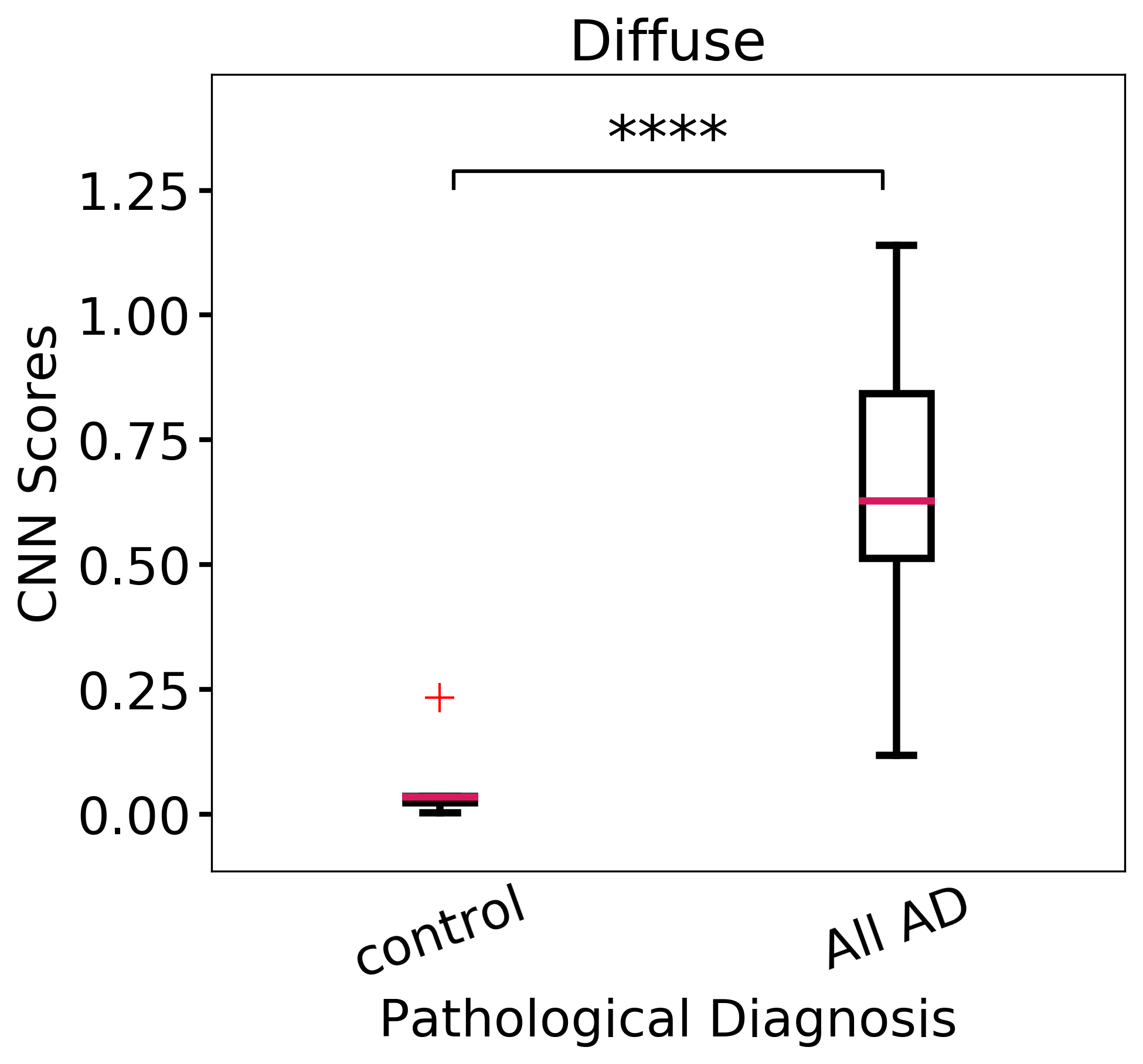

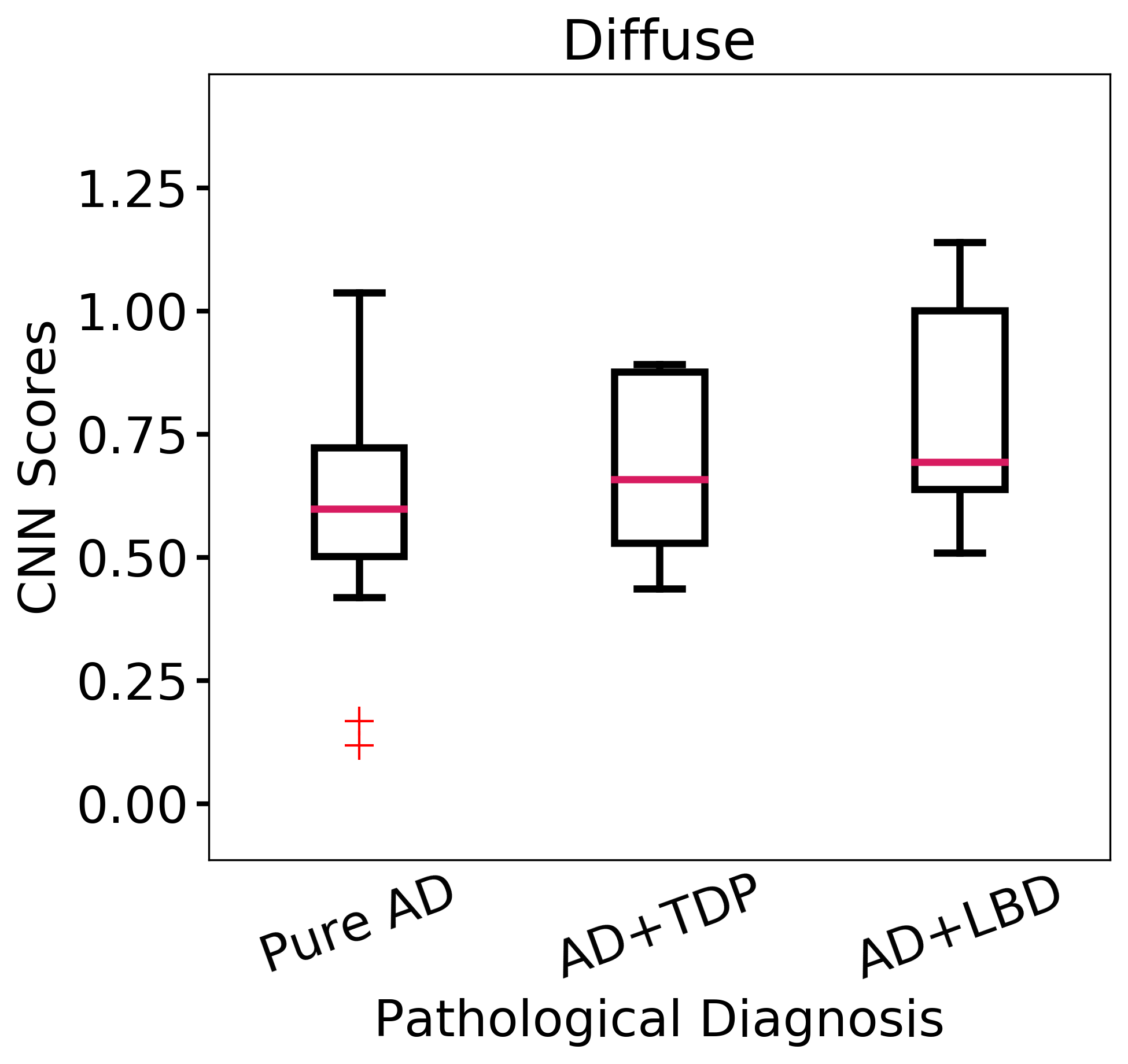


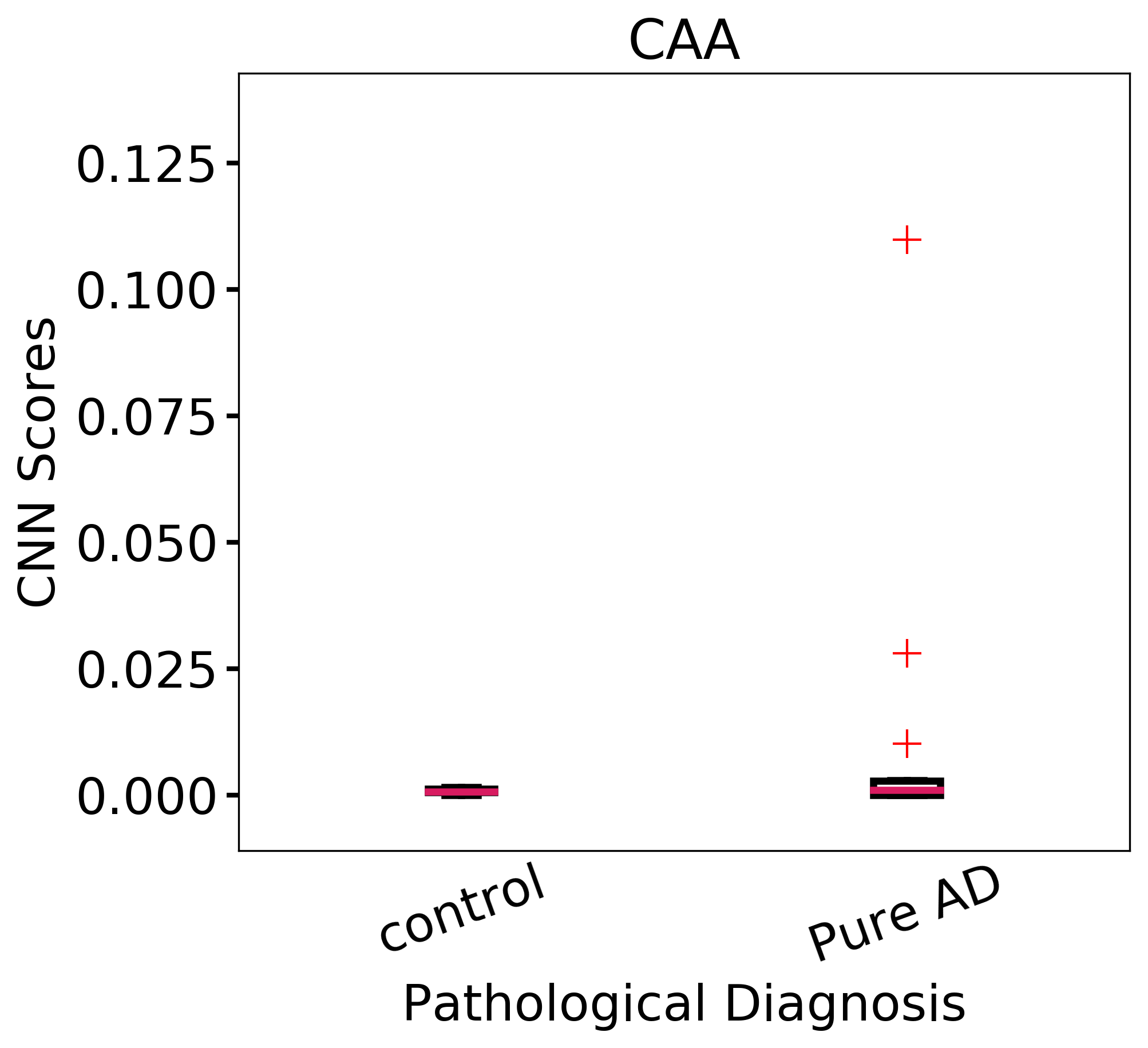

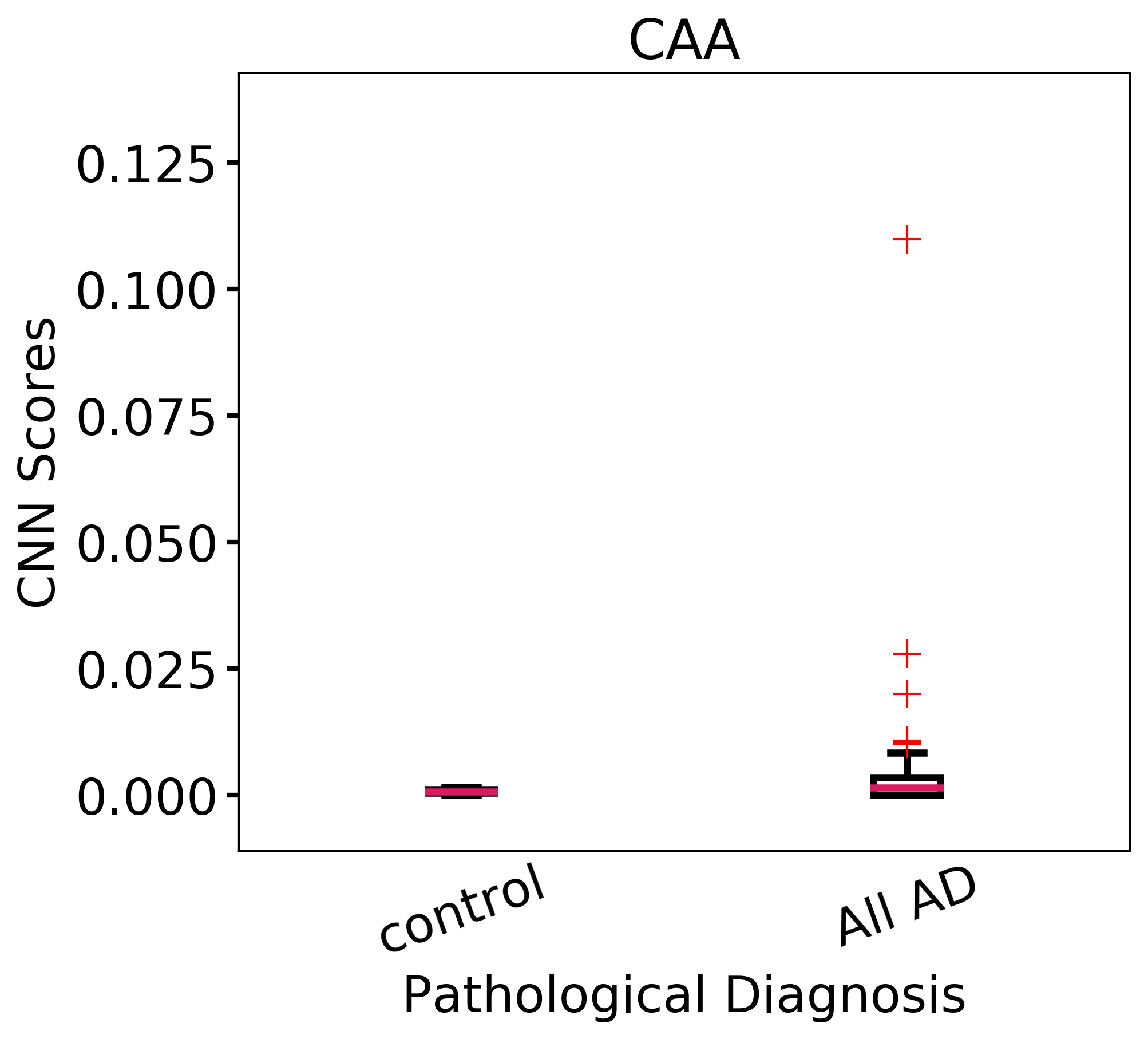

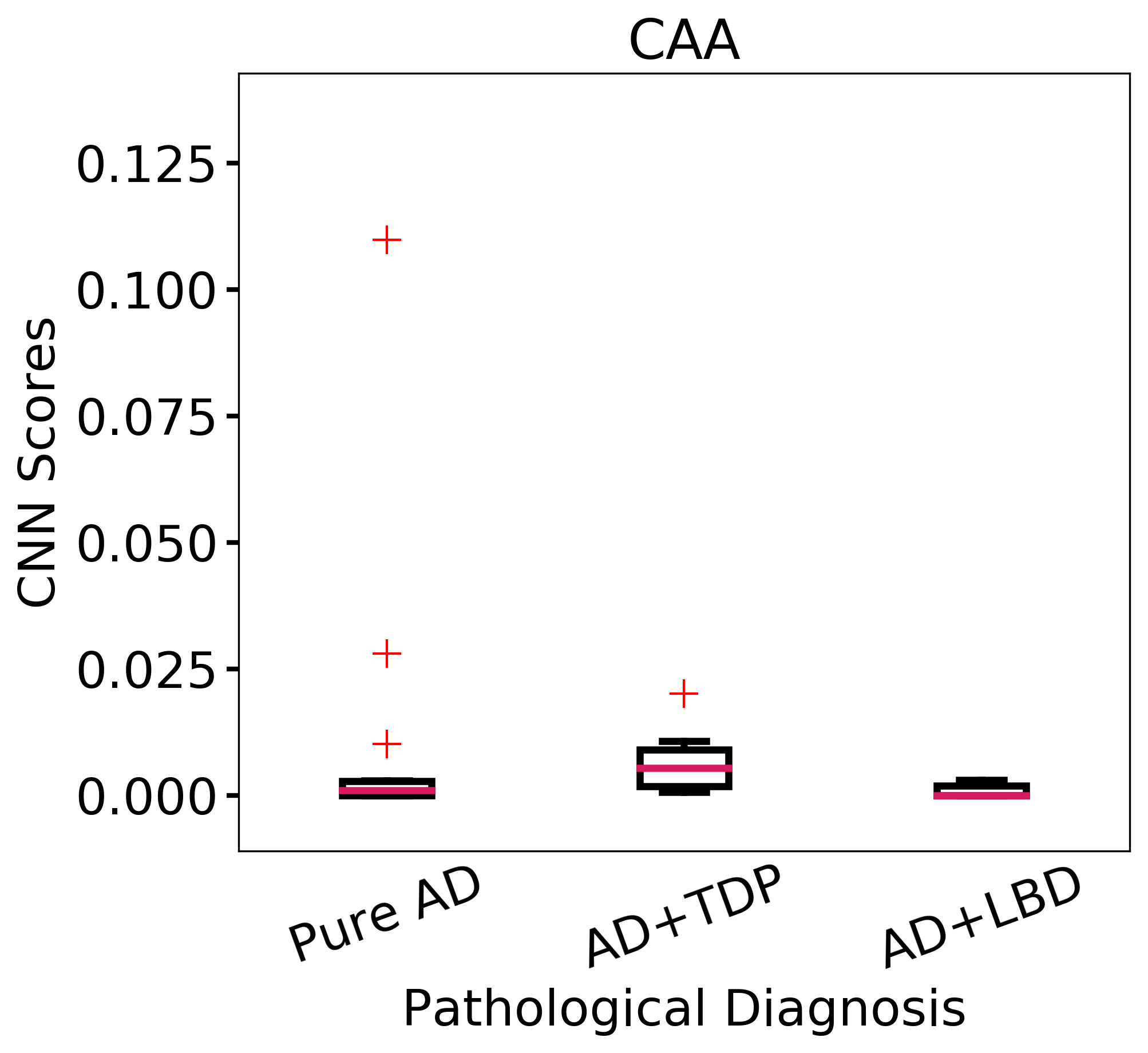


**Supplementary Fig. 7. Gray Matter CNN scores grouped by pathological diagnosis (Emory cohort).** CNN scores generated from confidence heatmap processing are grouped together by diagnosis of AD, concomitant AD, and control cases. AD: Alzhiemer’s disease, TDP: TDP-43 inclusions, LBD: Dementia with Lewy Bodies. Control vs pure AD and control vs all AD are statistically compared using a 2-sided independent sample t-test. An ANOVA with post-hoc Tukey’s test for multiple comparisons is used for pure AD vs AD+TDP vs AD+LBD comparison. Outliers are shown with red + and significance between groups is shown( * 0.05, ** 0.01, *** 0.001, **** 0.0001). Control (n=5), pure AD (n=14), all AD (n=30), AD+LBD (n=7), AD+TDP (n=8).

**
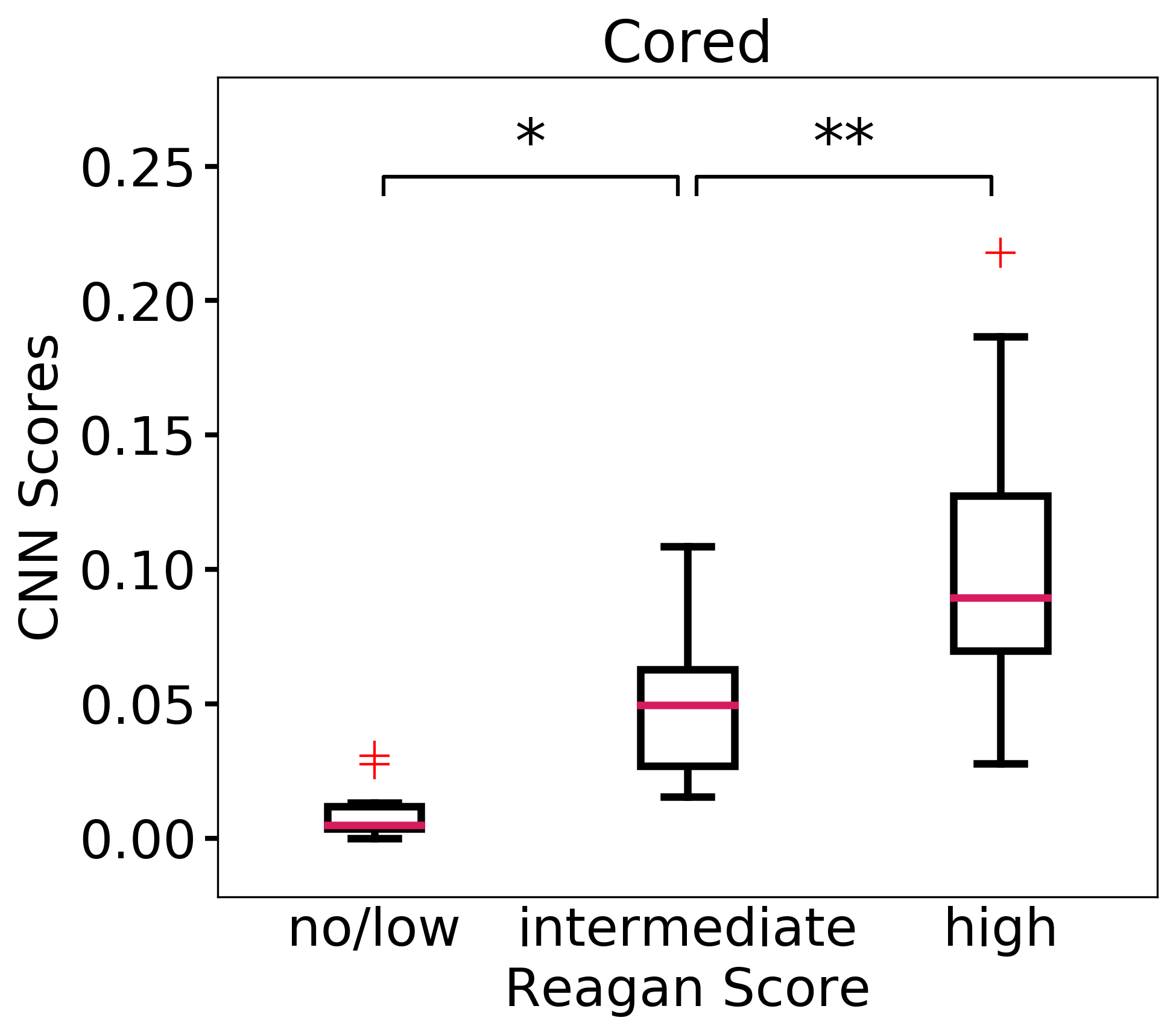

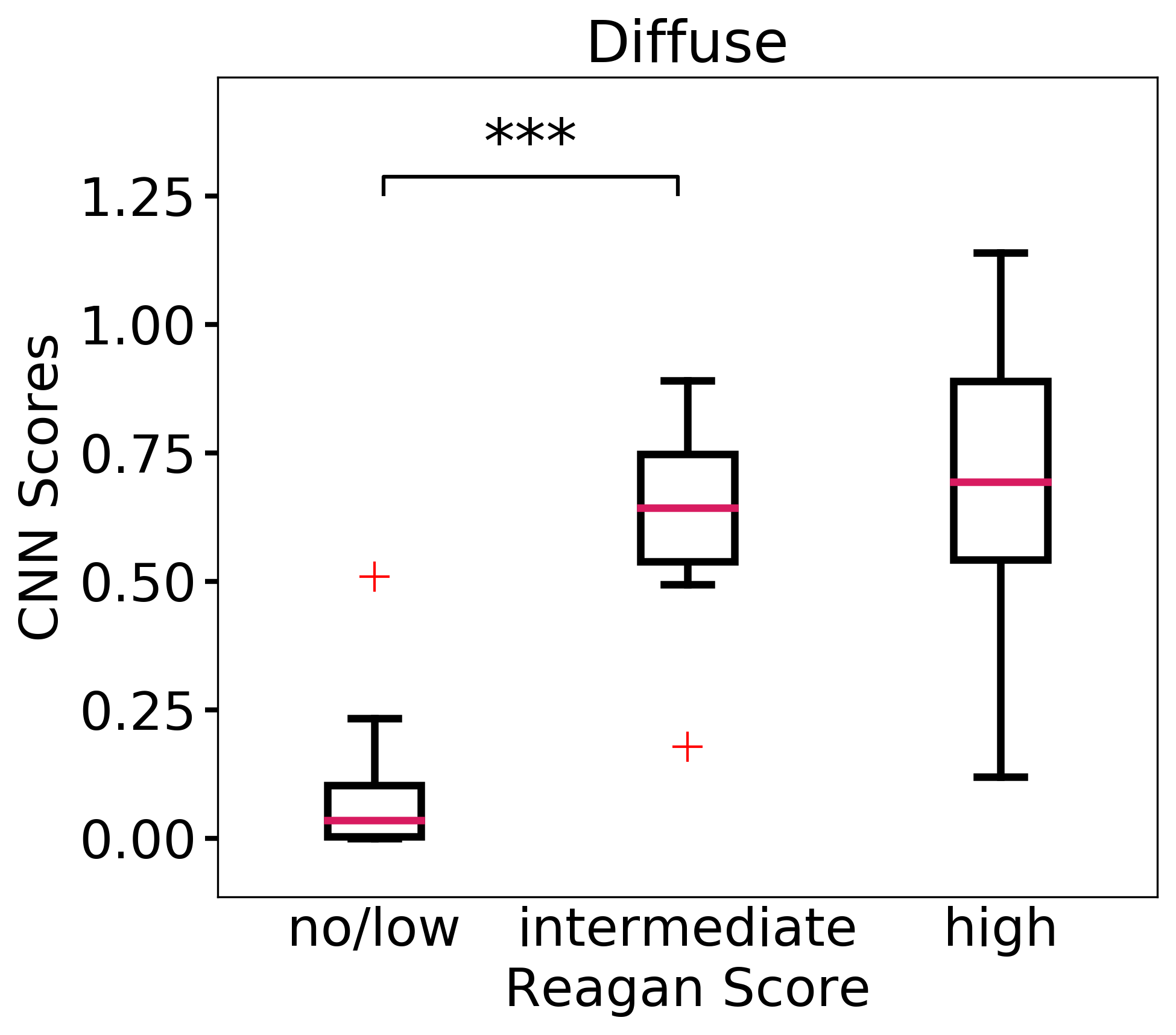

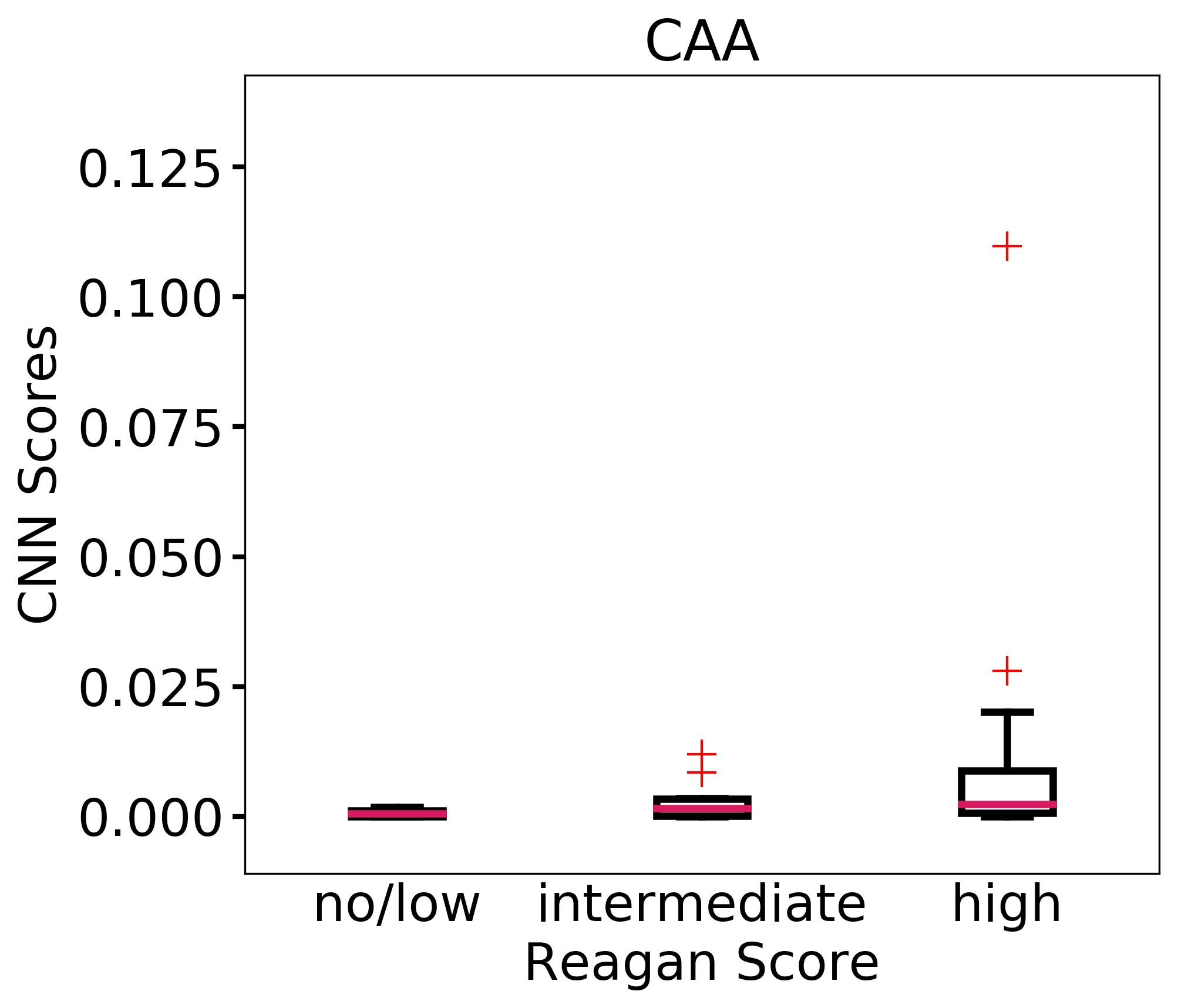
**

**Supplementary Fig. 8. Gray Matter CNN score grouped by Reagan criteria score for Emory data.** The whole tissue CNN scores for the Emory dataset are grouped together by their Reagan score, combining the no and low groups into one. Groups are compared using ANOVA with post-hoc analysis using Tukey’s test for multiple comparisons. Outliers are shown with red + and significance between groups is shown( * 0.05, ** 0.01, *** 0.001, **** 0.0001). no/low (n=11), intermediate (n=10), and high (n=19).


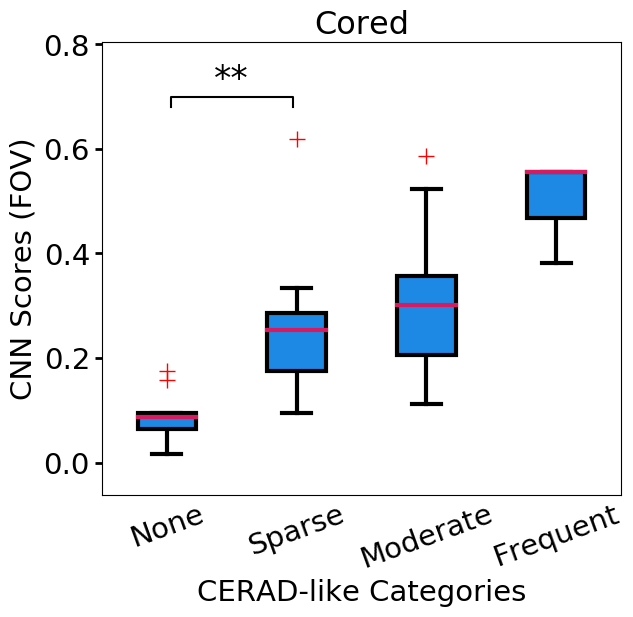

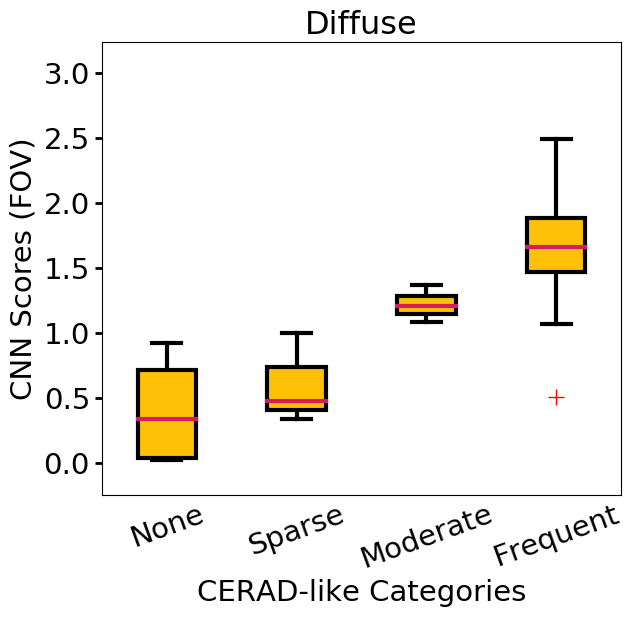

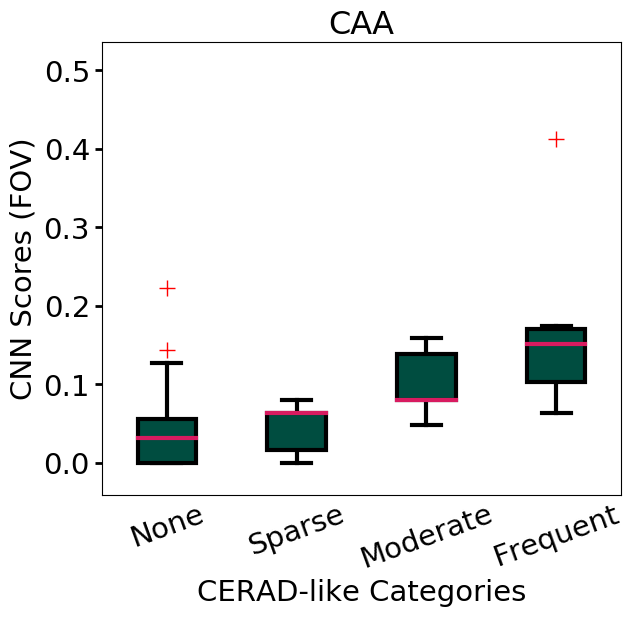


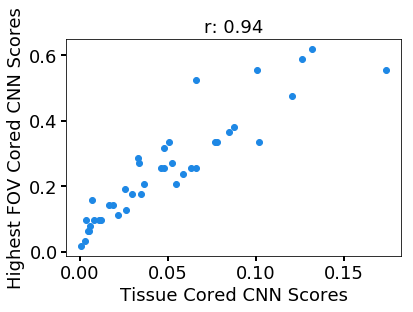

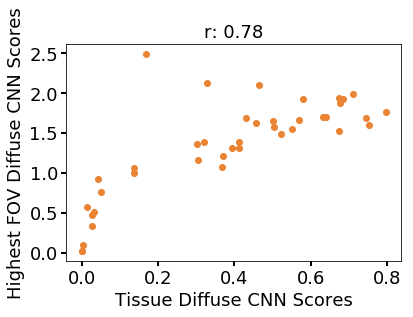

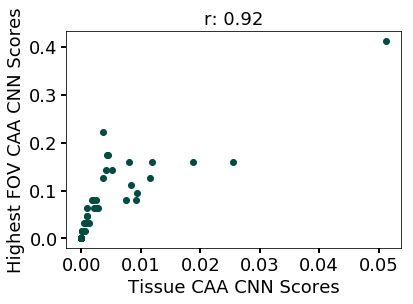


**Supplementary Fig. 9. Correlations between whole tissue CNN scores vs highest density FOV score.** Top row shows the highest FOV CNN-Score grouped by their CERAD-like scores. Results are similar to those seen when using the whole tissue scores. In cored plaques significance using Tukey’s post-hoc test (after ANOVA) is not seen between moderate and frequent groups as was seen for whole tissue scores. Bottom row shows the correlation of tissue CNN scores for all Emory data (n=40) vs the highest scored FOV score. Spearman correlation coefficient is shown on the top of each figure with left (cored), middle (diffuse), and right (CAA).

**
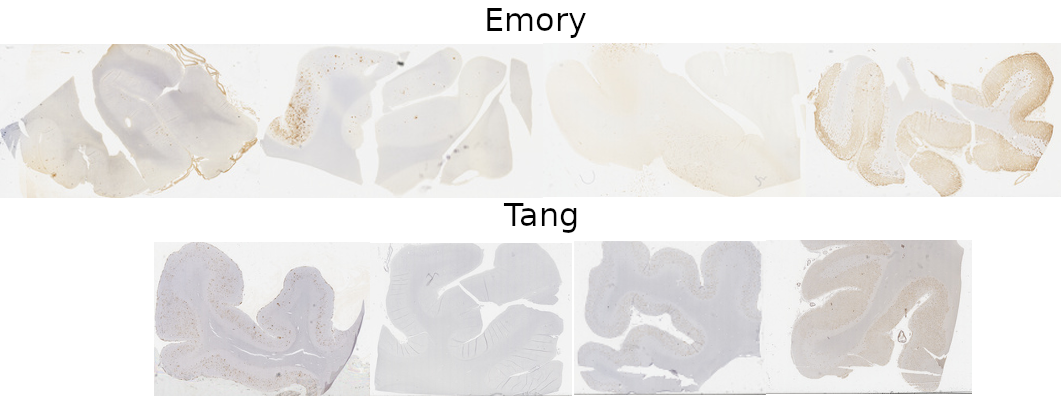
**

**Supplementary Fig. 10. Tinctorial differences between slides between the two institutions.** Sample low resolution images of the WSI used in this study. Bottom 4 images are from Tang *et al.* dataset which was used in the original work and the top 4 are from the Emory cohort.

**
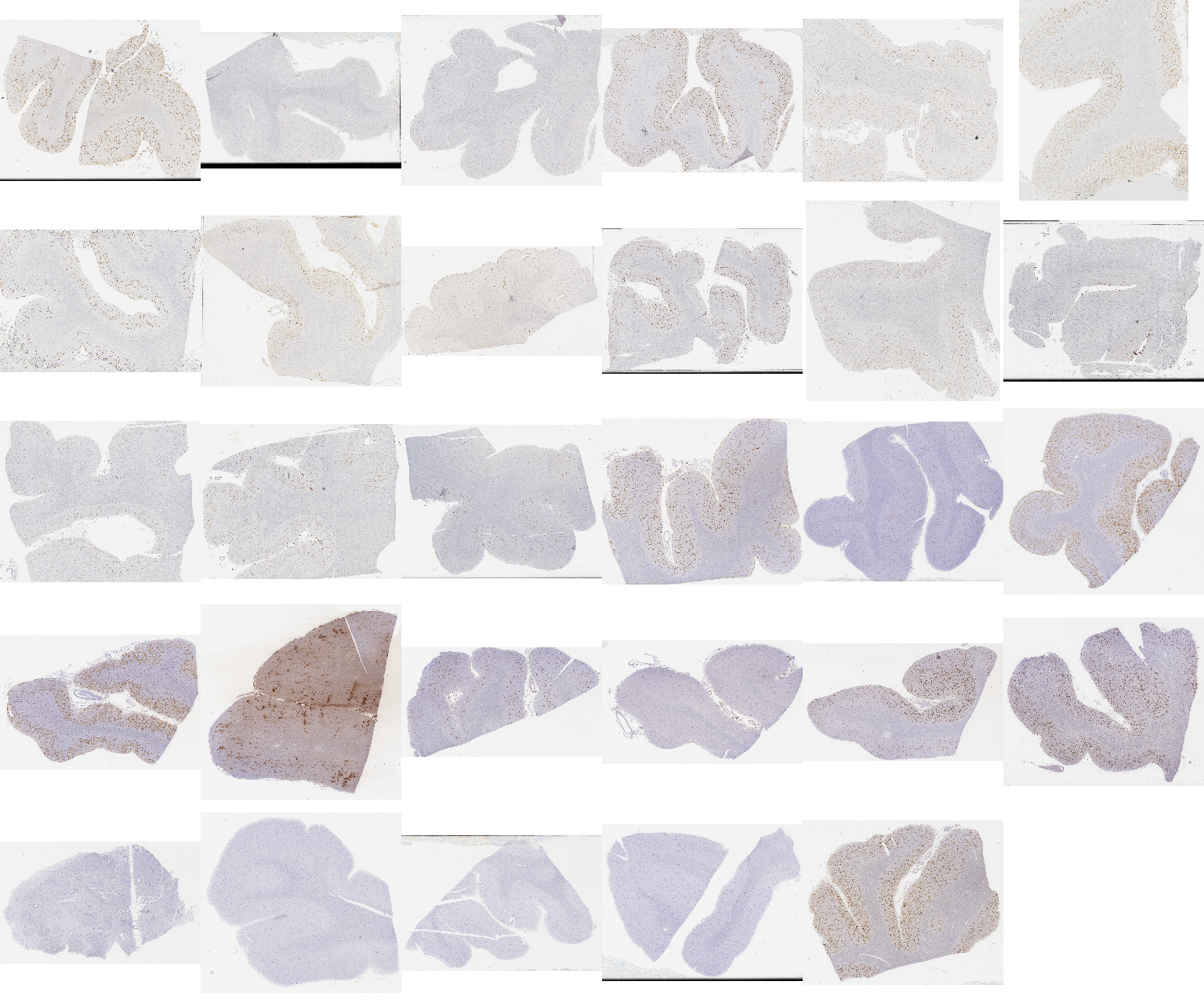
**

**Supplementary Fig. 11. Mosaic image showing all the WSI in Tang train dataset at low resolution.** These images were tiled and labeled for use in training the neural network.

**
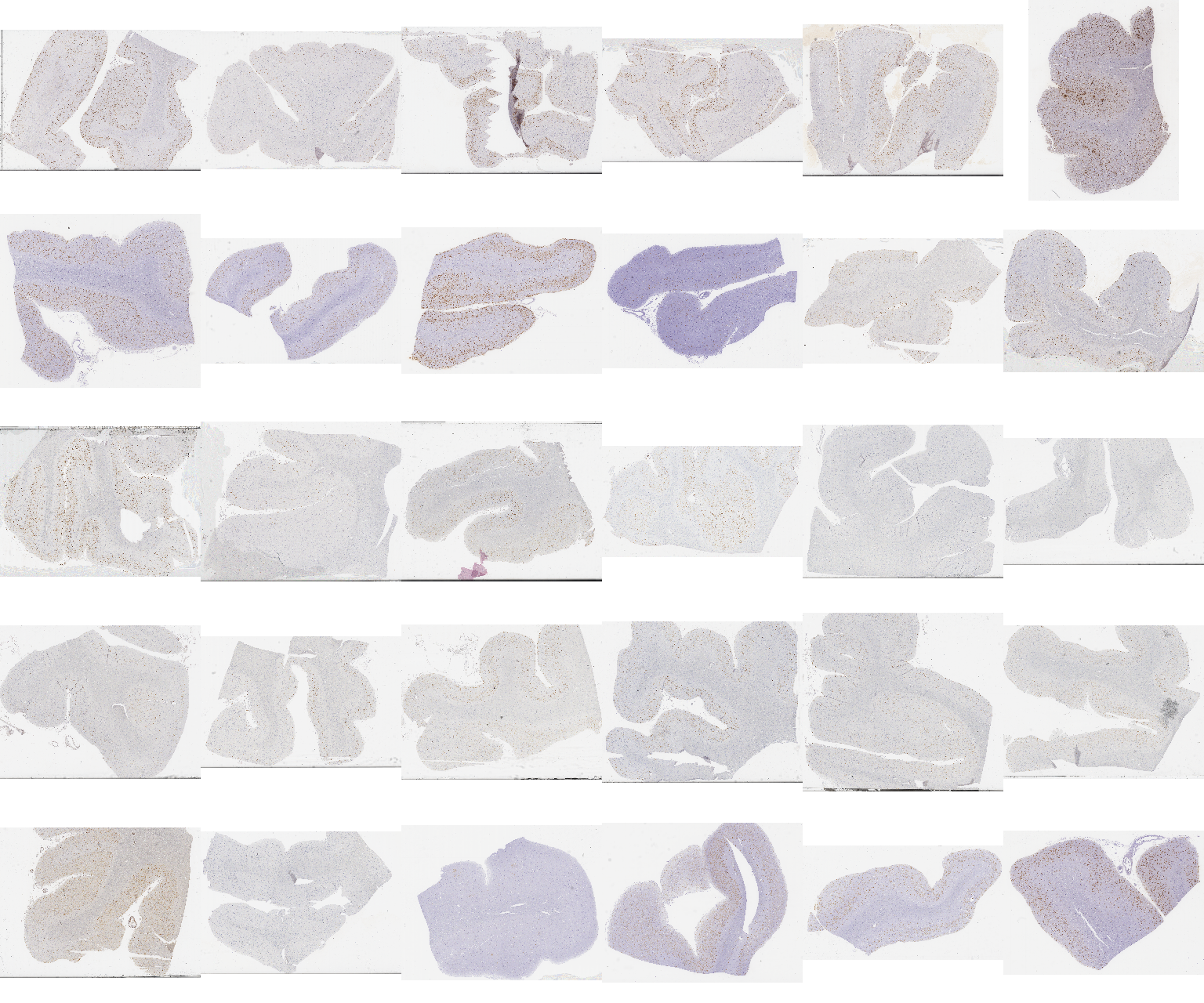
**

**Supplementary Fig. 12. Mosaic image showing all the WSI in Tang hold-out dataset.** Ten of these images were used in the CNN testing dataset (to produce the ROC and PRC). The 30 WSI were used to generate the heatmaps and CNN scores for the Tang dataset.

**
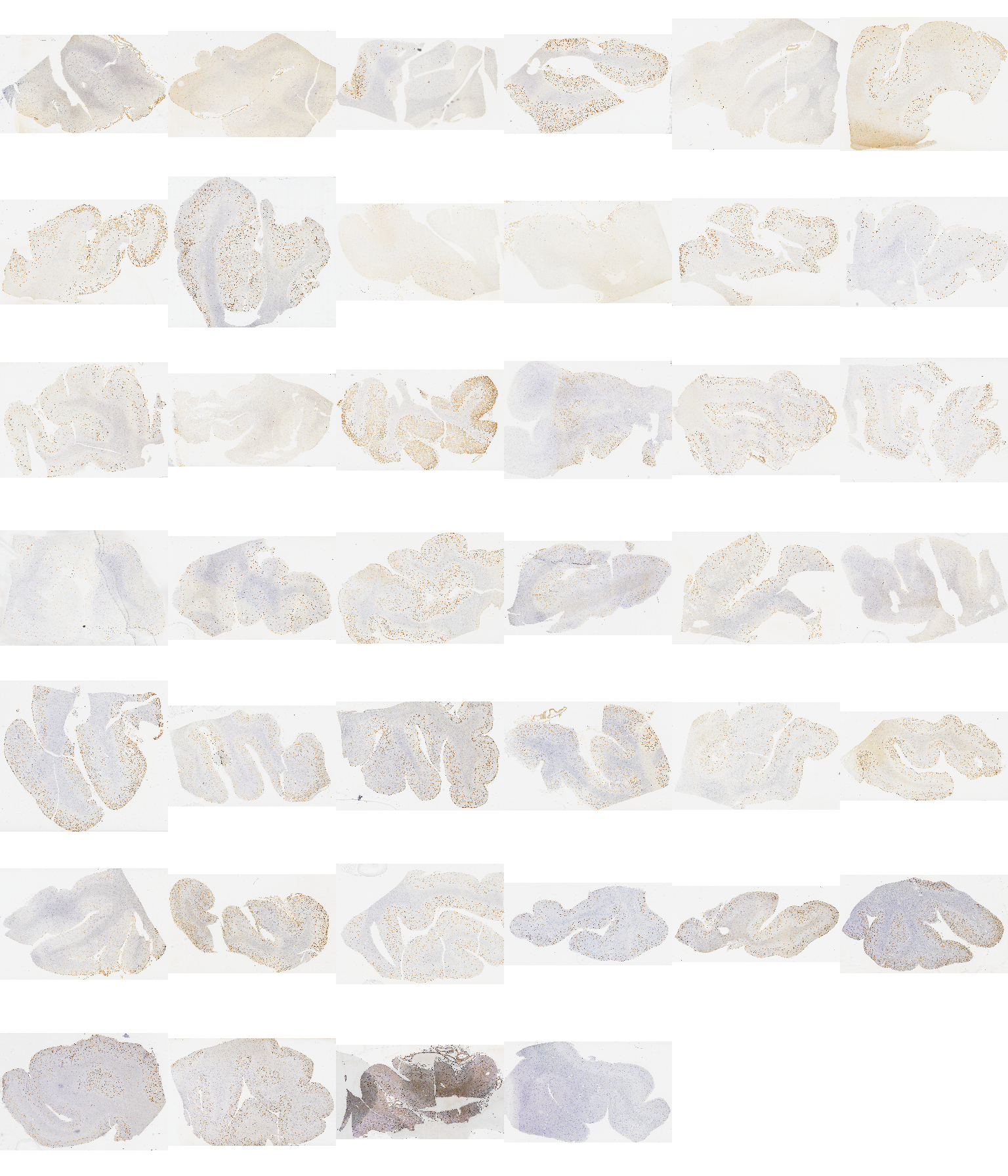
**

**Supplementary Fig. 13. Mosaic image showing all the WSI in the Emory dataset at low resolution.** These images were used solely for generating heatmaps and CNN scores using the CNN model trained on the Emory training dataset.

**(a) Train Dataset**


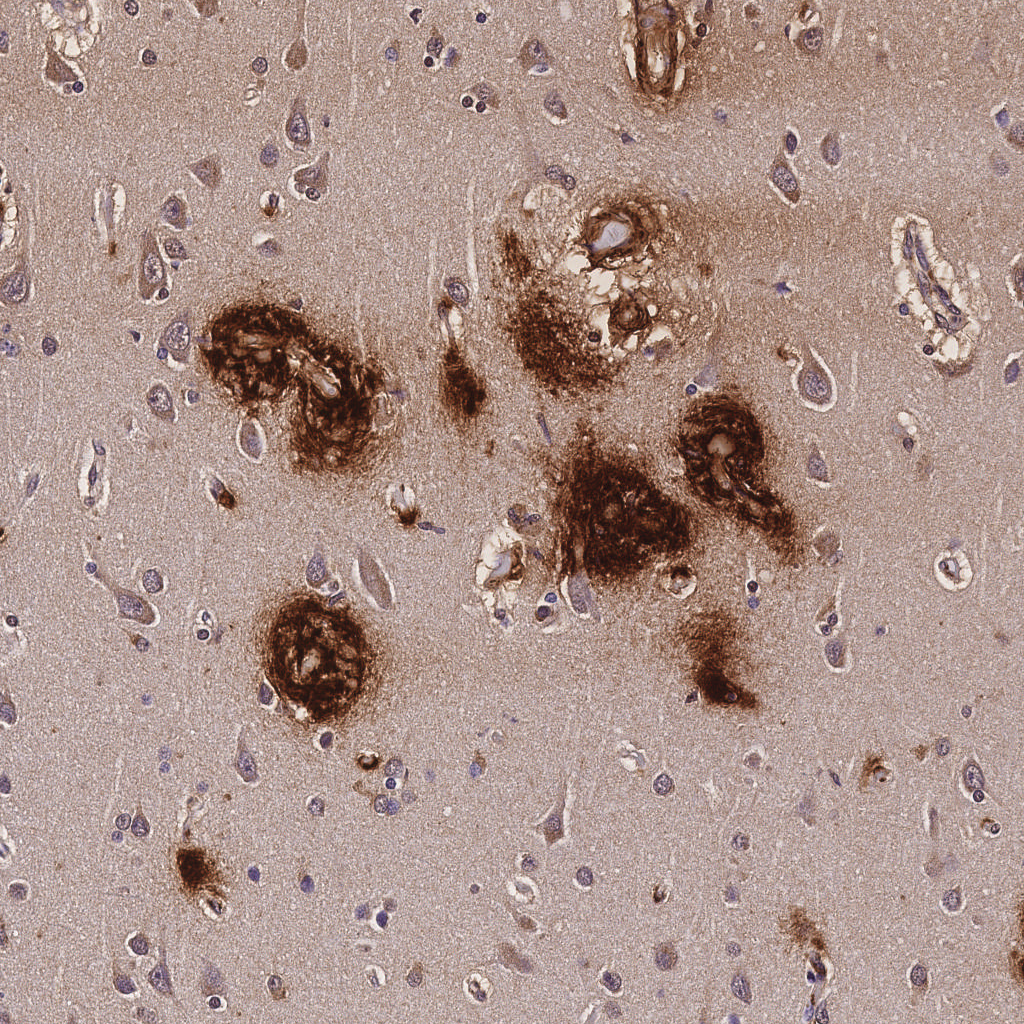

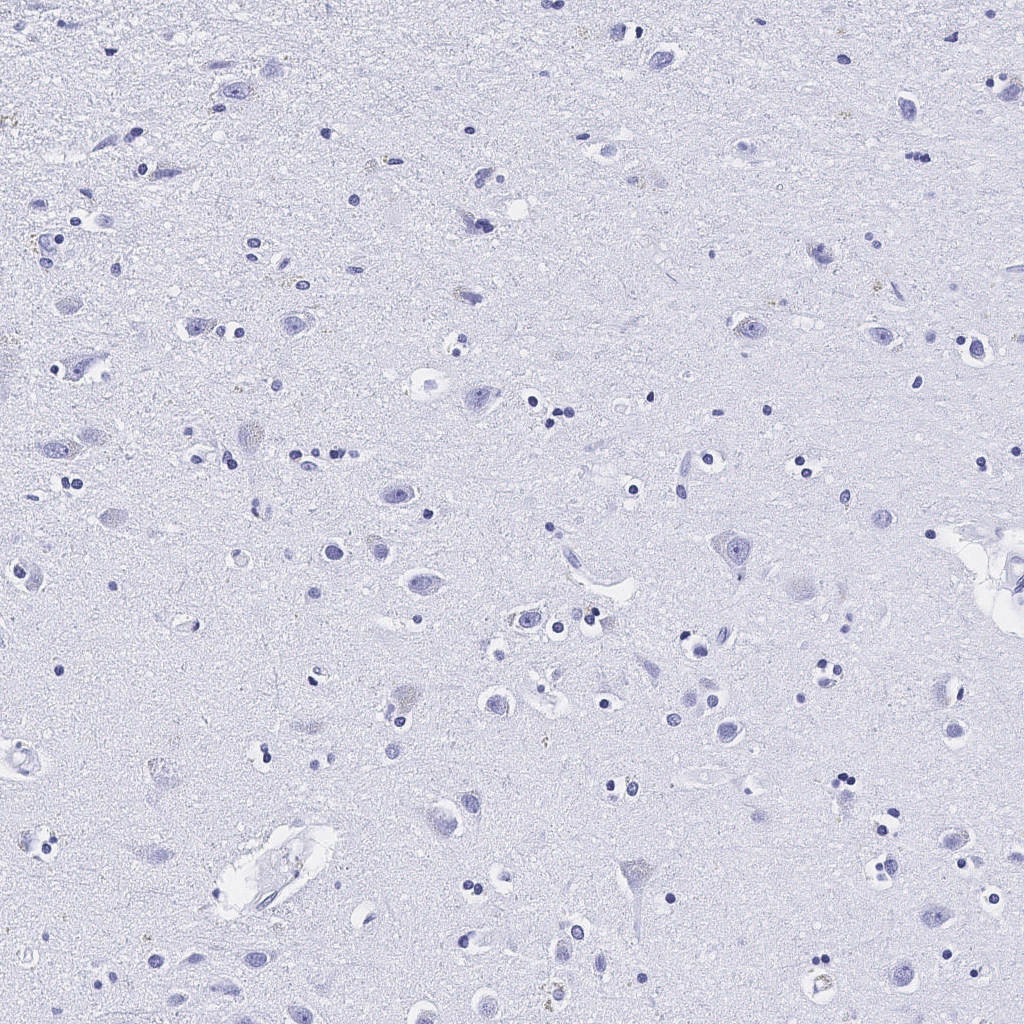

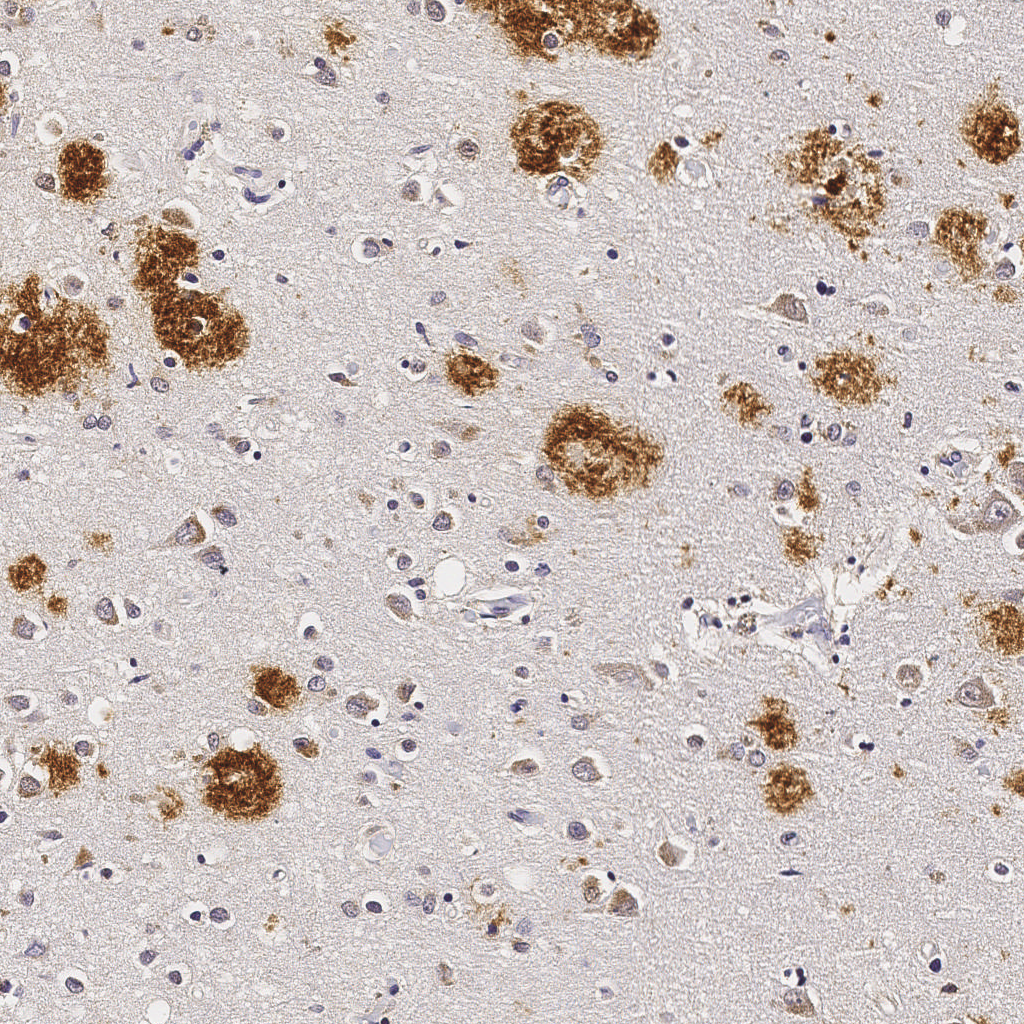

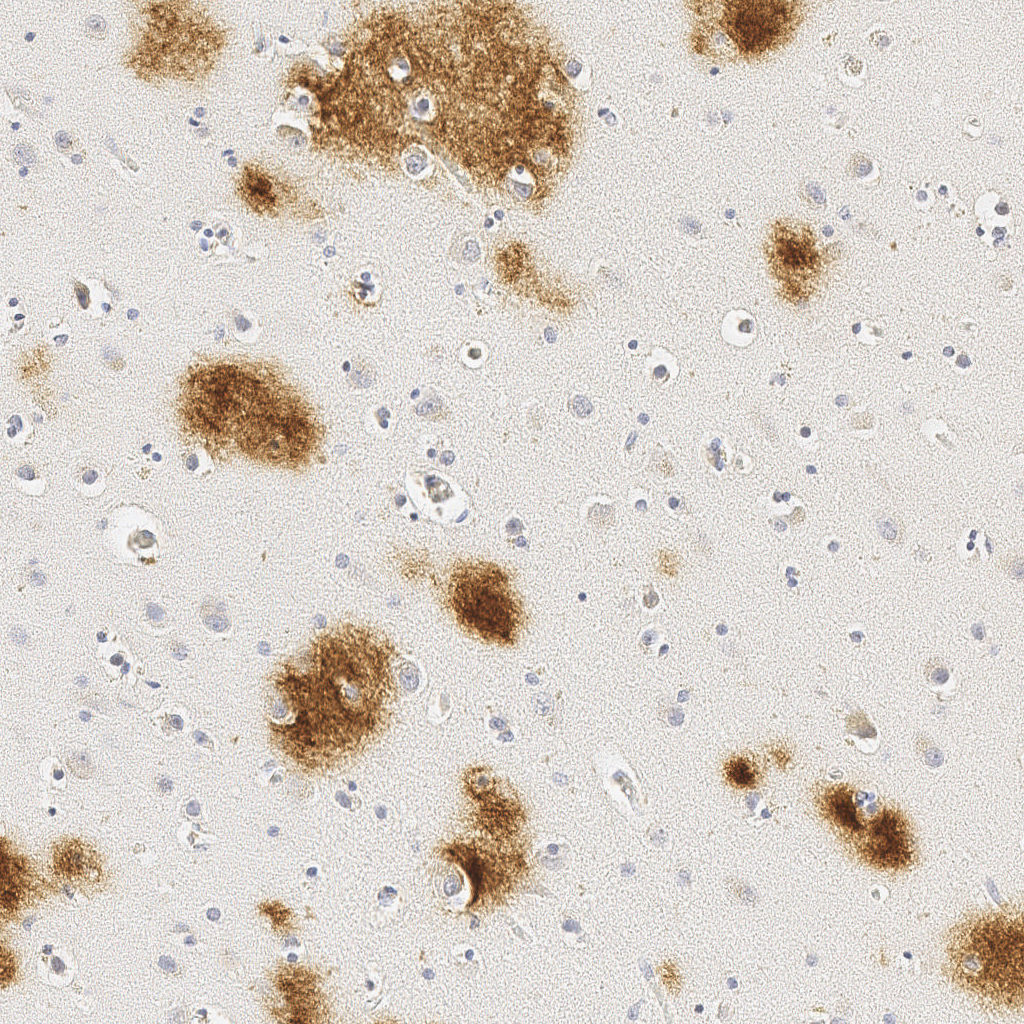


**(b) Tang Holdout Dataset**

**
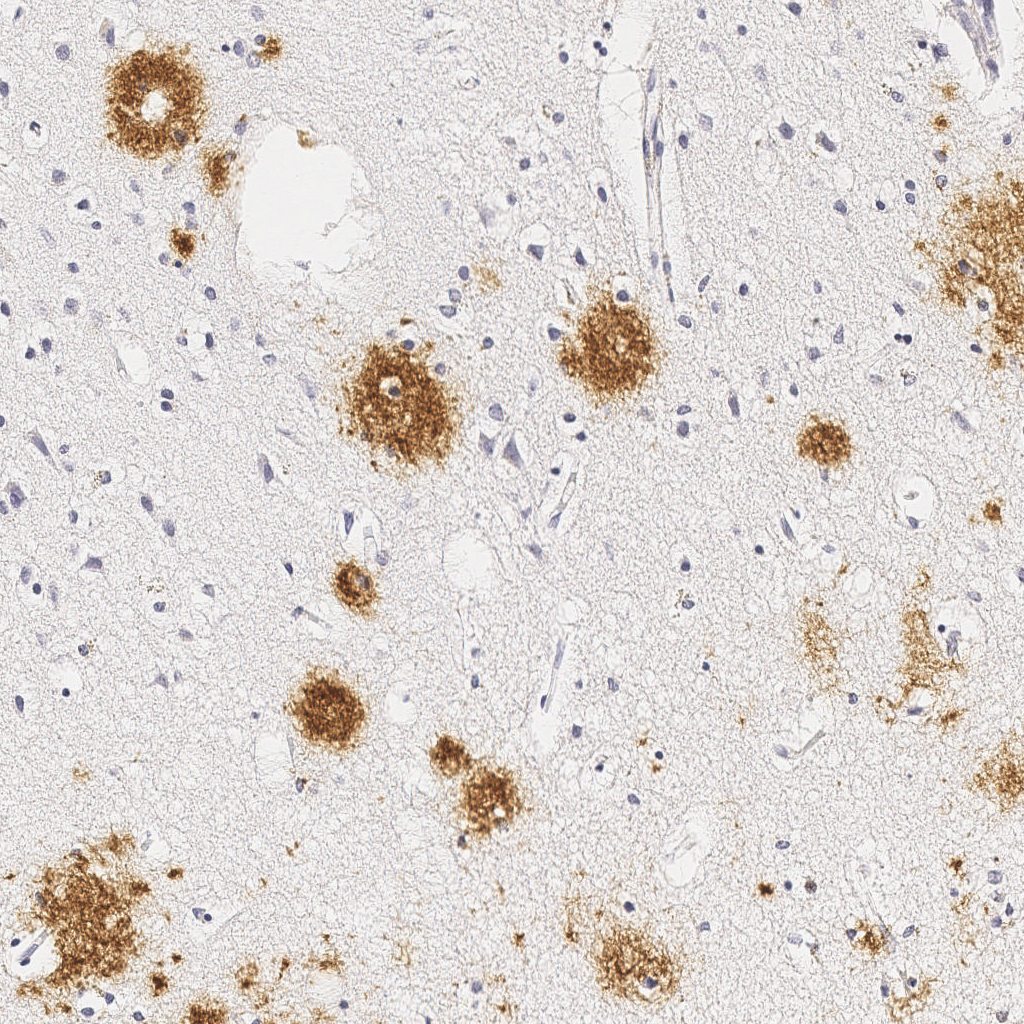

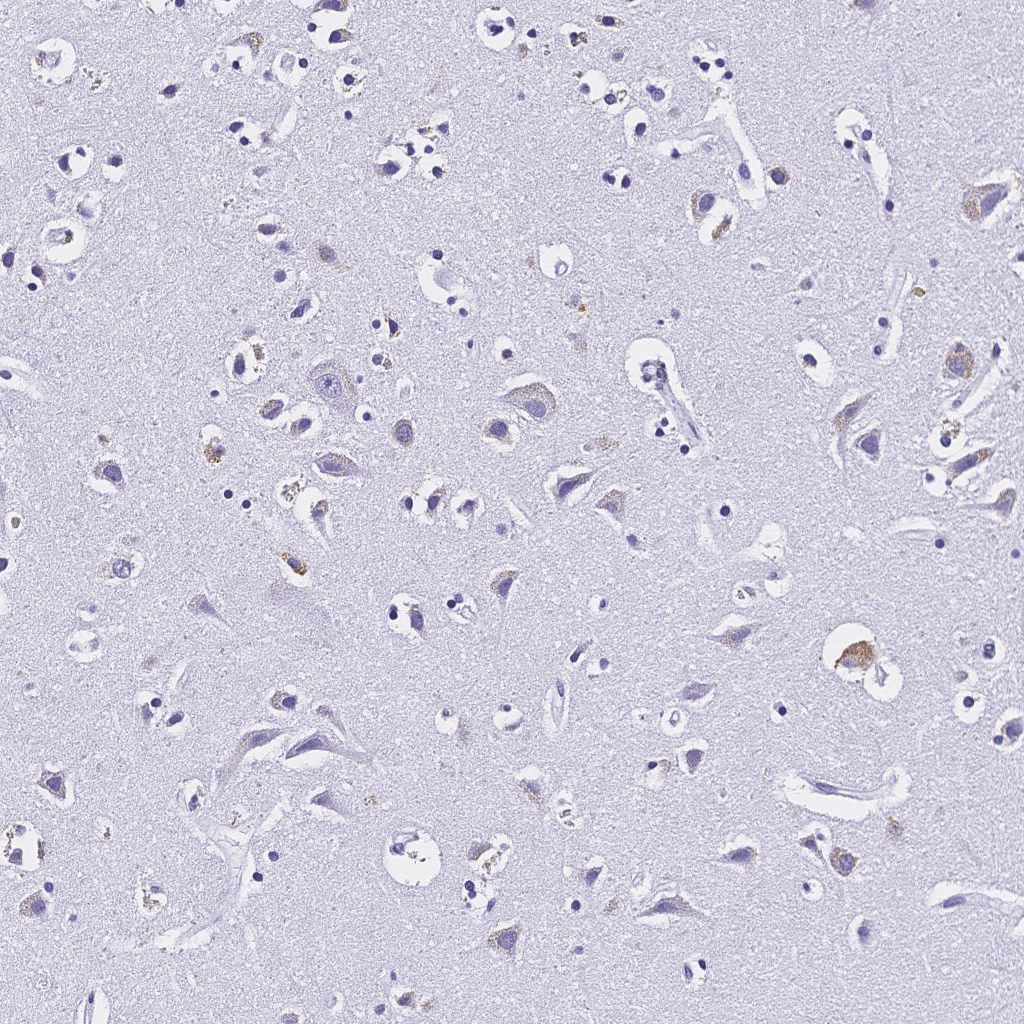

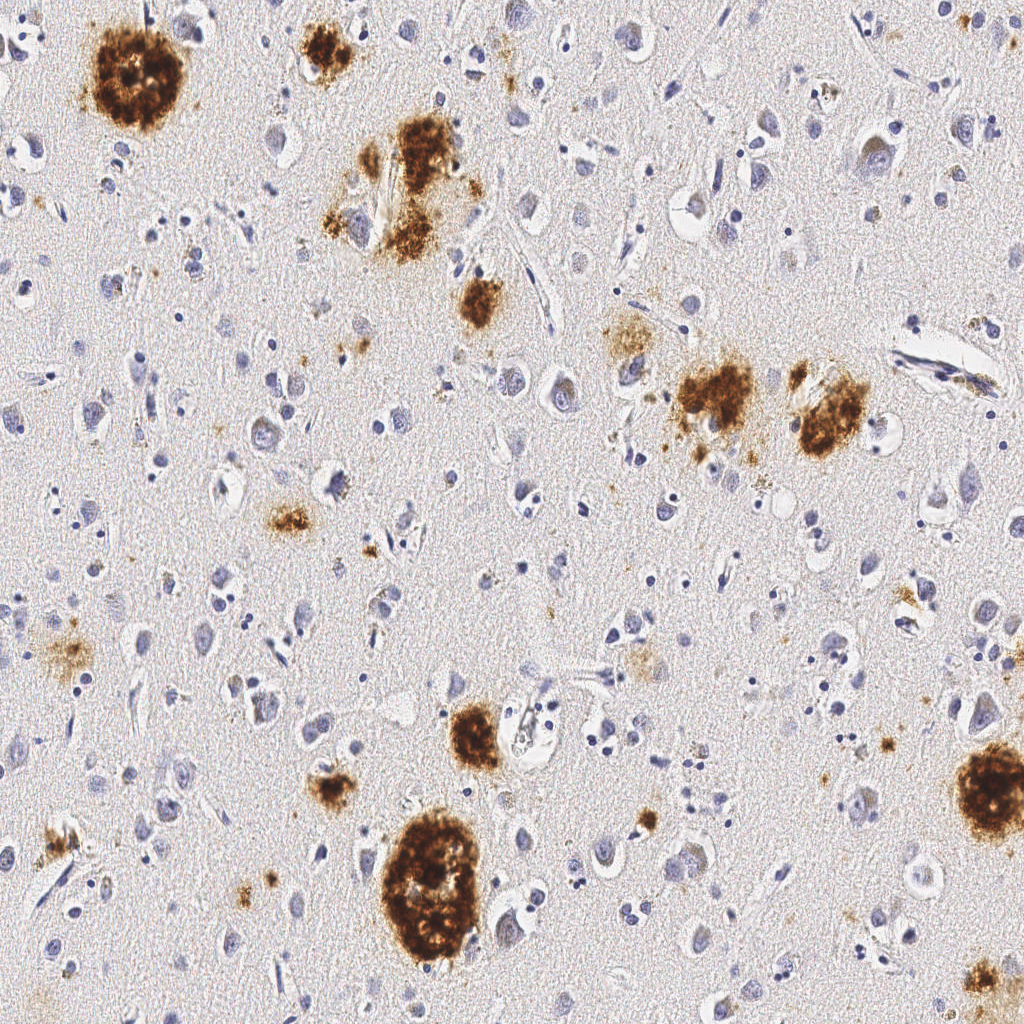

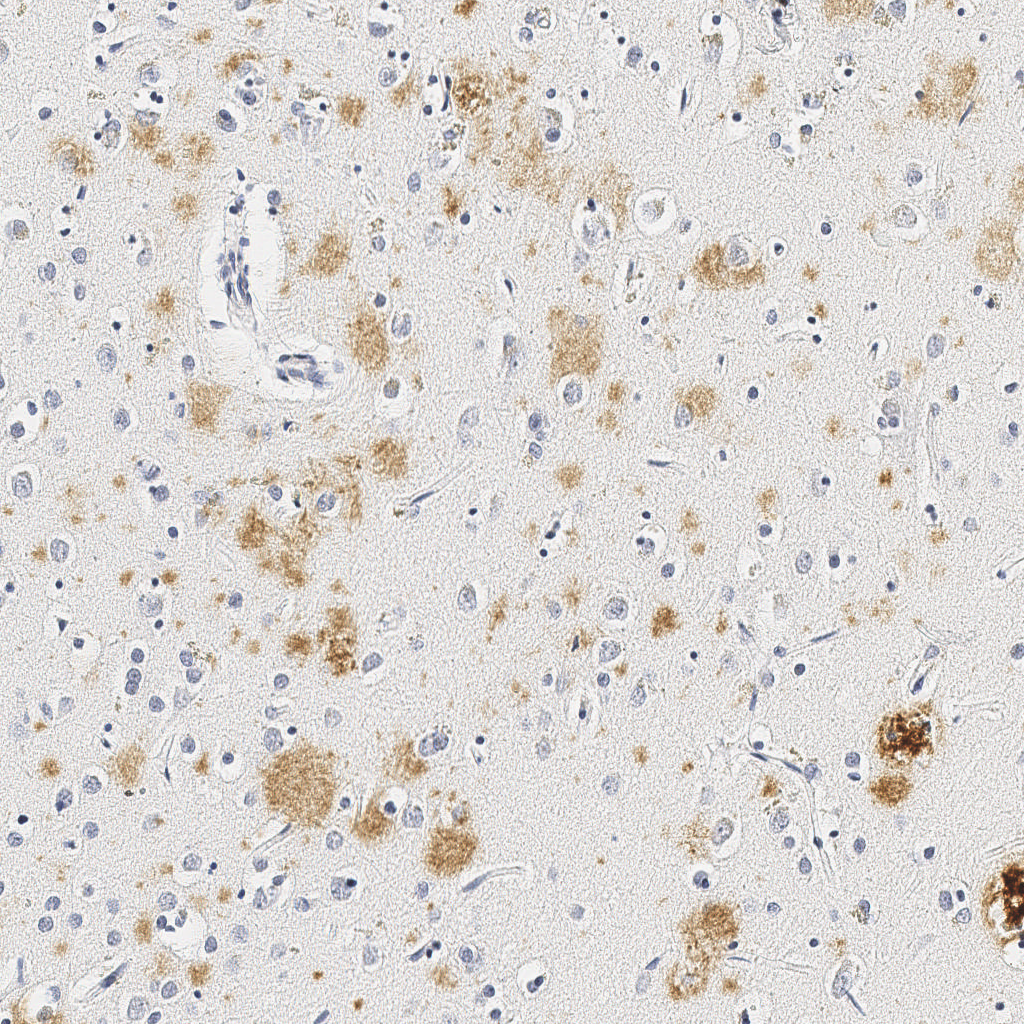
**

**(c) Emory Dataset**

**
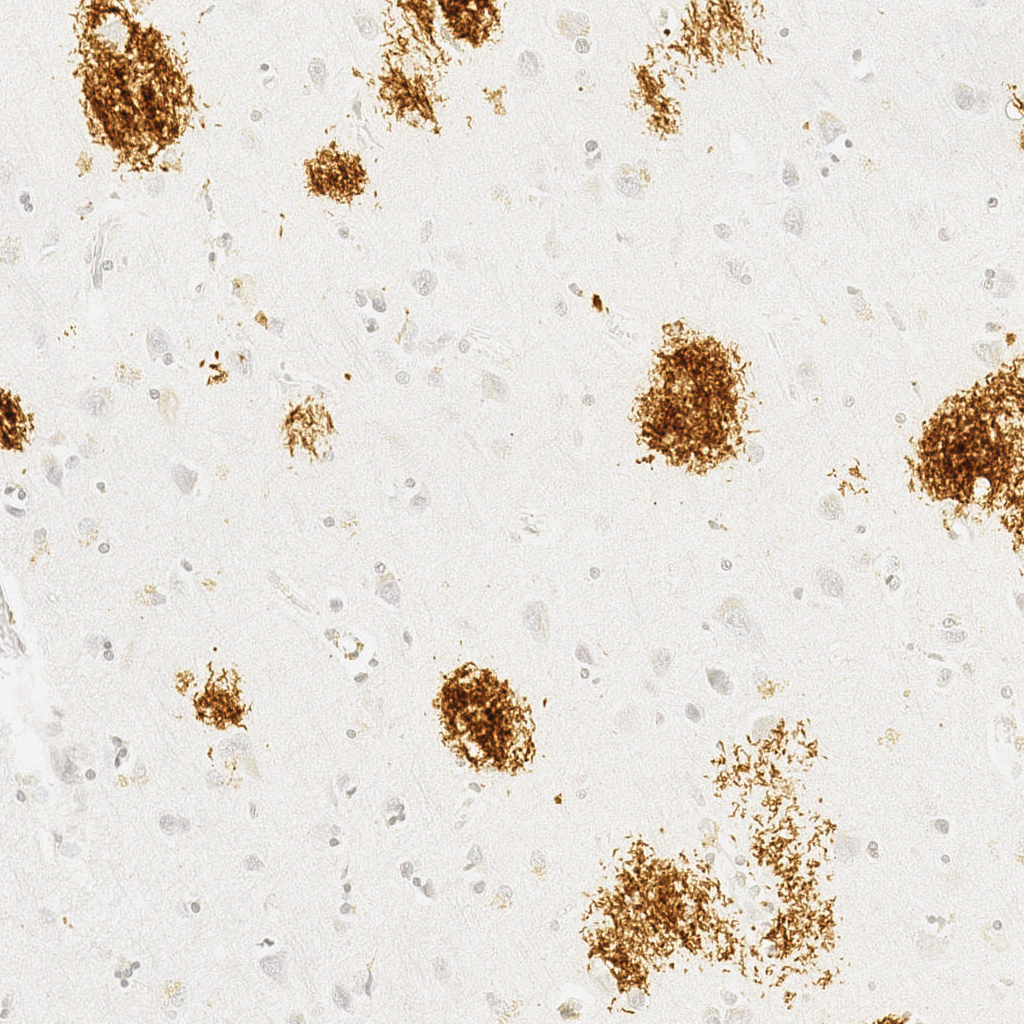

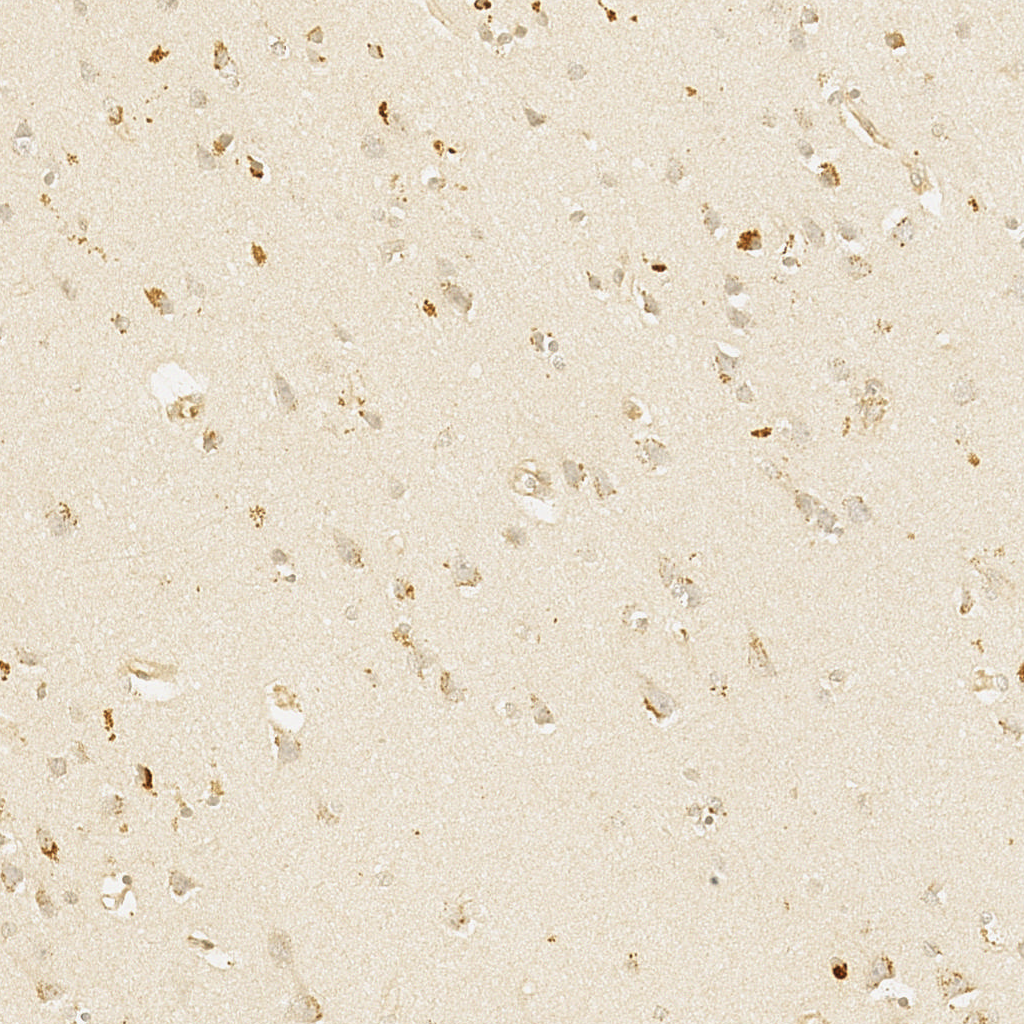

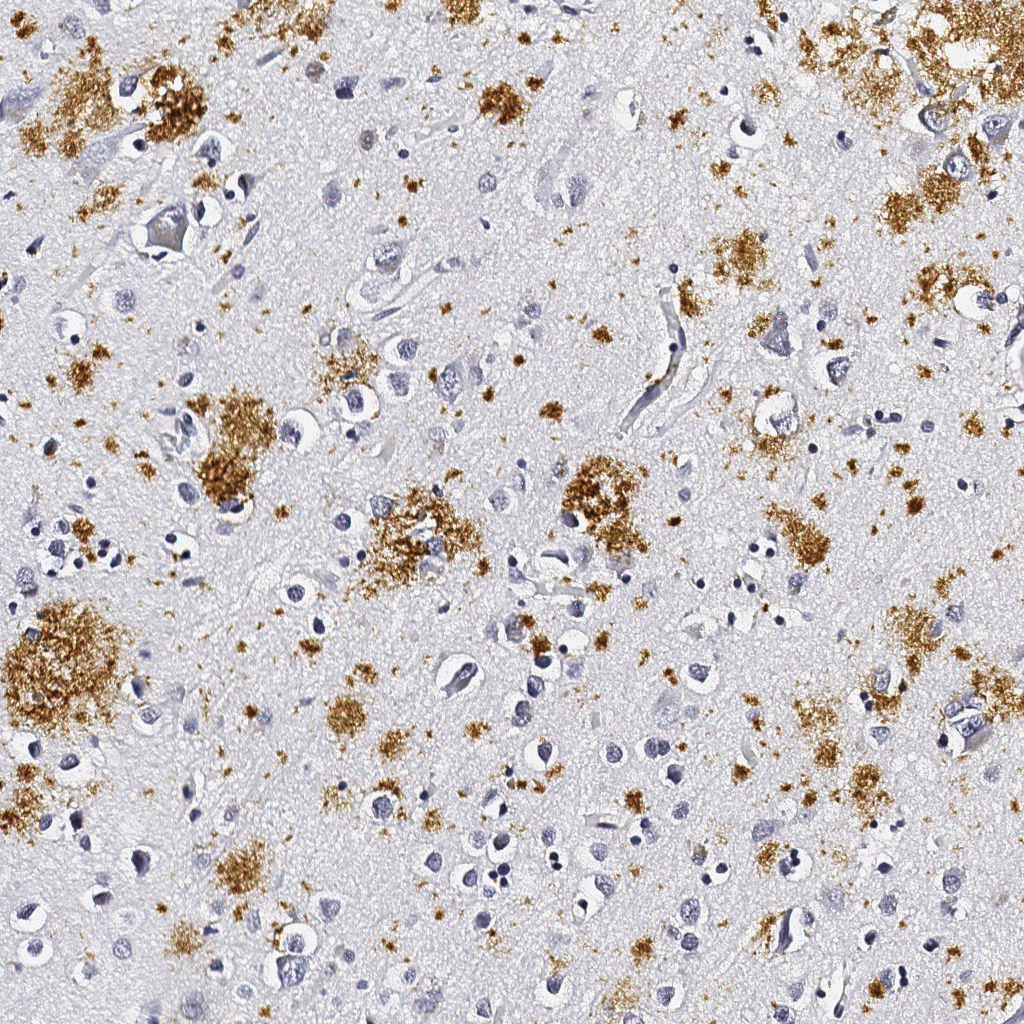

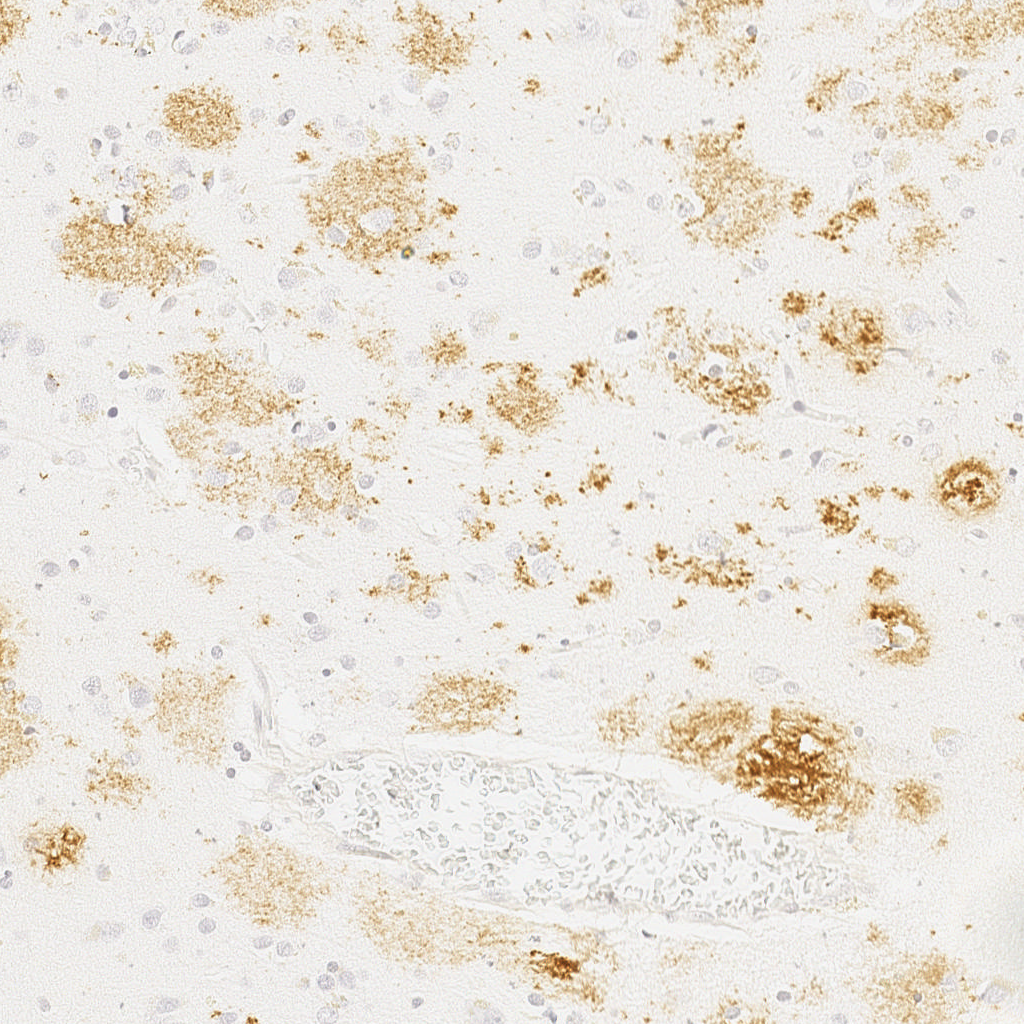
**

**Supplementary Fig. 14. High resolution image examples from WSI.** Examples were taken from the Tang training dataset (a), Tang hold-out dataset (b), and the Emory dataset (c). These examples were chosen to show the difference in tinctorial shift between datasets, fadeness between datasets, and counterstain variations between datasets (blue/purple nuclei staining).
